# A charge-dependent long-ranged force drives tailored assembly of matter in solution

**DOI:** 10.1038/s41565-024-01621-5

**Published:** 2024-03-01

**Authors:** Sida Wang, Rowan Walker-Gibbons, Bethany Watkins, Melissa Flynn, Madhavi Krishnan

**Affiliations:** 1 Physical and Theoretical Chemistry Laboratory, Department of Chemistry, University of Oxford, Oxford, UK; 2 The Kavli Institute for Nanoscience Discovery, Oxford, UK

**Keywords:** Surfaces, interfaces and thin films, Nanoparticles

## Abstract

The interaction between charged objects in solution is generally expected to recapitulate two central principles of electromagnetics: (1) like-charged objects repel, and (2) they do so regardless of the sign of their electrical charge. Here we demonstrate experimentally that the solvent plays a hitherto unforeseen but crucial role in interparticle interactions, and importantly, that interactions in the fluid phase can break charge-reversal symmetry. We show that in aqueous solution, negatively charged particles can attract at long range while positively charged particles repel. In solvents that exhibit an inversion of the net molecular dipole at an interface, such as alcohols, we find that the converse can be true: positively charged particles may attract whereas negatives repel. The observations hold across a wide variety of surface chemistries: from inorganic silica and polymeric particles to polyelectrolyte- and polypeptide-coated surfaces in aqueous solution. A theory of interparticle interactions that invokes solvent structuring at an interface captures the observations. Our study establishes a nanoscopic interfacial mechanism by which solvent molecules may give rise to a strong and long-ranged force in solution, with immediate ramifications for a range of particulate and molecular processes across length scales such as self-assembly, gelation and crystallization, biomolecular condensation, coacervation, and phase segregation.

## Abstract (web summary)

In contrast to textbook expectations, experimental findings show that, in certain situations, like-charged particles may either repel or attract each other depending on the sign of their charge and on the solvent.

## Main

The delicate interplay of interactions between objects in the fluid phase influences the behaviour, organization and properties of systems from nanometric to more macroscopic size and length scales and thus underpins a wealth of natural phenomena. Our understanding and intuition of the interaction between electrically charged particles in solution is grounded in a central principle from classical electromagnetics which dictates that the force between charges of the same sign is not only repulsive at all separations but is also symmetric with respect to the sign of the charge. For example, because like-charged objects in vacuum are expected to repel regardless of whether the sign of the charge they carry is positive or negative, the expectation is that like-charged particles in solution must also monotonically repel, particularly at long range where the van der Waals (vdW) attraction is too weak to substantially influence the overall interaction. This view is a hallmark of the Derjaguin–Landau–Verwey–Overbeek (DLVO) theory, a cornerstone of colloid science^1–3^.

Nonetheless, clear and well-founded exceptions to the rule exist in the presence of multivalent ions^4,5^ and in particular regimes involving high magnitudes of electrical charge and in low dielectric constant media^6^. But over the decades, consistent reports of attraction between like-charged particles from the nanometre to micrometre scales in size and range, for example, nucleic acids, liposomes, polymers and colloidal particles in aqueous media containing low concentrations of monovalent salt—where DLVO theory is expected to hold—have evaded explanation^7–17^. Not surprisingly, this persistent divergence between experiment and theory has received considerable attention in the theoretical literature^6,18–23^.

**Fig. 1:**
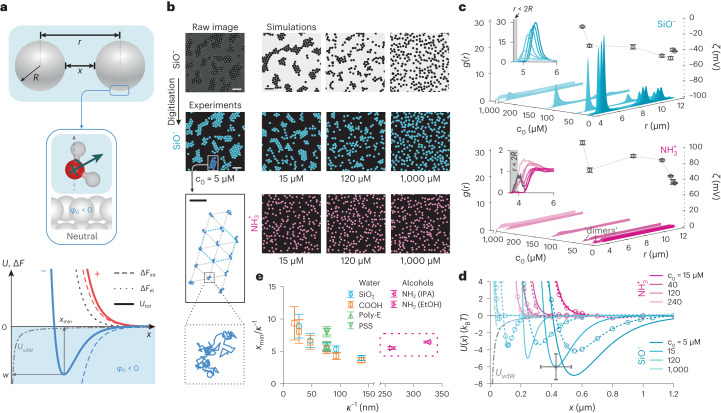
Interparticle interactions in solution can break charge-reversal symmetry. **a**, Schematic representations of two interacting spheres (top), and of the dominant average orientation of water molecules near an uncharged surface (middle). Bottom: Various contributions to the total interaction free energy for positively (red lines) and negatively charged (blue lines) particles: electrostatic repulsion, ∆*F*_el_ (black dotted line; applies to both signs of charge); interfacial solvation contribution, ∆*F*_int_, depends on the sign of charge (dashed lines); total interaction energy, ${U}_{{\rm{tot}}}={\Delta F}_{{\rm{el}}}+{\Delta F}_{\mathrm{int}}$ (solid lines); and the vdW contribution, *U*_vdW_ (dashed-dotted grey line; applies to both cases). For *φ*_0_ < 0 (for example, in water) negatively charged particles may display a minimum in *U*_tot_ of depth *w* at a separation *x*_min_ (solid blue line), and positively charged particles repel monotonically (red solid line) (Supplementary Information, section 3.1). **b**, Negatively charged silica particles (SiO^−^, blue circles, *R* = 4.82 μm) form hcp clusters in water depending on salt concentration, *c*_0_ (middle row). Scale bars, 20 μm. Inset, bottom left: trajectories of particles in a single cluster over a period of 30 s. Scale bar, 5 μm. Positively charged aminated silica particles (${\rm{N}}{{\rm{H}}}_{3}^{+}$, pink circles, *R* = 3.92 μm) in water do not form clusters regardless of *c*_0_ (bottom row). Images acquired using bright-field microscopy (Supplementary Figs. 3 and 25) and digitized to facilitate presentation. BD simulation snapshots for SiO^−^ particle interactions (top). **c**, Measured radial probability density functions, *g*(*r*), for SiO^−^ (top) and ${\rm{N}}{{\rm{H}}}_{3}^{+}$ particles (bottom), including measured zeta potentials (*ζ*; circular symbols) for varying *c*_0_. Error bars denote ±s.d for three measurements. Insets: the small peak around 2*R* reflects particle ‘dimers’ in the sample. **d**, Inferred pair-interaction potentials, *U*(*x*) (solid lines), for SiO^−^ (blue) and ${\rm{N}}{{\rm{H}}}_{3}^{+}$ particles (pink). Calculated *U*_tot_(*x*) curves as described in ref. ^27^ and Supplementary Information, sections 3.2 and 3.3 (dashed lines with symbols; see Supplementary Table 1 for parameter values). Error bars denote estimated uncertainties of ±100 nm on particle diameter and ±1.5*k*_B_*T* in *w* (Supplementary Fig. 18). **e**, Plot of *x*_min_ versus *κ*^−1^ for various negative particles in water and positive particles in alcohols shows that ${x}_{\min }\approx 5-10{\kappa }^{-1}$ (Fig. 5). Errors bars represent an uncertainty of ≈50 nm in *x*, averaged over all particle types.

Standing theories invoke a continuum description of the solvent which overlooks finer-grained detail such as the molecular nature of the solvent, and its structure and interactions, particularly at an interface with a molecule or a particle^1,2^. Despite the general success of classical mean-field, continuum theories^3^, it is becoming increasingly clear that the molecular nature of water ought to play a defining role in a host of interfacial phenomena in the aqueous phase^24–26^. We recently suggested that the behaviour of the molecular solvent at an interface can make a substantial contribution to the total interaction free energy of two approaching objects carrying electrical charge^27,28^ (Fig. 1a). In particular, for charged matter in water, our model suggested that this contribution could be large enough not only to counteract the interparticle Coulombic repulsion but could even overwhelm it, changing the sign of the interparticle force for negatively charged particles, turning it net attractive. The orientational behaviour of the interfacial solvent could therefore be viewed as a store of free energy that can be tapped by the interparticle interaction, even at large separations (Fig. 1a). We further suggested that this interfacial free energy contribution results in a force whose sign and magnitude may be given in terms of an interfacial electrical potential, *φ*_0_, that depends on the nature of the solvent, with *φ*_0_ < 0 for water at an interface (refs. ^27–29^) (Fig. 1a; see Supplementary Information, section 3.1 for details).

The main experimental trends suggested by the interfacial solvation model are: (1) the breaking of charge-reversal symmetry in interparticle interactions, for example, negatively charged particles may attract in water, whereas positive particles repel; (2) the use of a different solvent may reverse this trend, that is, positive particles may attract whereas negatives repel; and (3) the magnitude of the solvation contribution to the interparticle interaction should depend strongly on the pH in solution. Here we report on comprehensive experimental tests of the contribution of the interfacial solvent to long-range interactions in solution. We demonstrate that the solvent can make a large contribution to the interparticle interaction and can counterintuitively drive the spontaneous formation of ordered assemblies of like-charged particles in solution. Furthermore, both the sign and magnitude of this free-energy contribution can have a decisive impact on the ability of a system of particles to self-assemble, and crucially we show that they are both amenable to careful tuning using a variety of particle- and solution-dependent parameters.

## Measuring interparticle interactions in a two-dimensional suspension

**Fig. 2:**
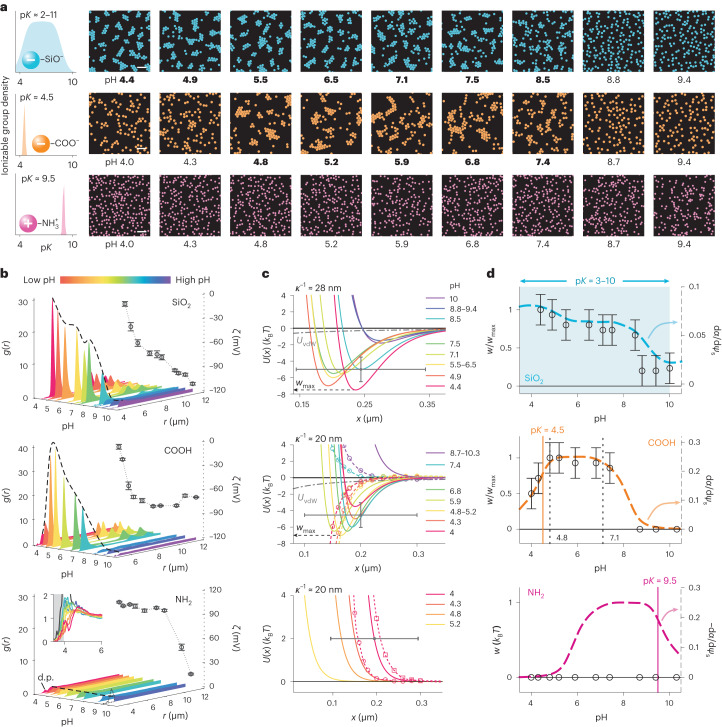
Tuning the interparticle interaction using the pH in solution. **a**, Snapshots of colloidal suspension structure as a function of pH for silica particles (top), carboxylic acid particles (middle) and aminated silica particles (bottom), holding salt concentration constant in each data series: *c*_0_ = 0.12 mM (SiO_2_), 0.25 mM (COOH) and 0.25 mM (NH_2_). Scale bars, 20 μm. Bold values indicate the pH at which hcp cluster formation is observed. **b**, Radial probability density functions *g*(*r*) (curves) and measured zeta (*ζ*) potentials (symbols) as a function of pH for all three particle types. Error bars represent mean ± s.d. from three measurements. The increasing height of the ‘dimer-peak’ (d.p.) with increasing pH for positive particles is indicative of increased sticking of particles due to discharging of the basic ionizable groups (bottom panel). **c**, *U*(*x*) profiles inferred from measured *g*(*r*) profiles (solid lines; Supplementary Figs. 20–22 and Supplementary Tables 4–8), theoretical *U*_tot_(*x*) curves (dashed lines with symbols) calculated as described in ref. ^27^ and Supplementary Information, sections 3.2 and 3.3 (see Supplementary Table 1 for parameter values), and *U*_vdW_ contribution for comparison (dashed-dotted grey line). *w*_max_ denotes the maximum inferred depth of the experimentally observed minimum in each case. Error bars denote estimated uncertainties of ±100 nm in particle diameter and ±1.5*k*_B_*T* in *w* (Supplementary Figs. 20–22). **d**, Plots comparing trends as a function of pH in the experimentally inferred quantity *w/w*_max_ (symbols, left axis) with that of $\frac{{\rm{d}}\alpha }{{\rm{d}}{\psi }_{{\rm{s}}}}$ determined using Supplementary Information equation (9) (dashed curves, right axis). See Supplementary Fig. 25 for all raw image data.

We performed a range of experiments on two-dimensional (2D) suspensions of colloidal particles in solution and examined the dependence of the interparticle interaction on system properties such as pH and ionic strength of the electrolyte, particle charge and chemistry, and on the solvent molecular structure. We observed the spatial structure of the suspension using bright-field optical microscopy (Supplementary Information, section 1.5 and Supplementary Fig. 3) and constructed radial probability density distributions, *g*(*r*), from the spatial coordinates of particles in a series of images. To infer the form of the underlying interparticle interaction, we performed Brownian dynamics (BD) simulations of interparticle interactions with various input potentials, *U*(*x*), assuming pairwise additivity of the interactions, and generated simulated *g*(*r*) functions (Supplementary Information, sections 2.1 and 3.4). Here, *r* denotes the interparticle separation between spheres of radius *R*, at an intersurface separation *x* (Fig. 1a). An iterative process permitted us to identify pair-potentials that were qualitatively consistent with the experimental data (Fig. 1b, top row; Supplementary Figs. 18–24 and Supplementary Tables 2–12). We also compared these experimentally inferred pair-potentials with a total interaction free energy, *U*_tot_(*x*), calculated for a pair of identical particles using the interfacial solvation model^27^ (dashed lines with symbols in Figs. 1, 2 and 5, see Supplementary Information, sections 3.2 and 3.3). As implied by Supplementary Information equations (5)–(9), varying the value of *p* = p*K* − pH in the theoretical model may result in a calculated *U*_tot_(*x*) that is purely repulsive, purely attractive, or a non-monotonic function displaying a minimum of depth *w* at an intersurface separation *x*_min_. Parameter values for all calculated curves in this study are given in Supplementary Table 1.

## Negative particles attract and positives repel in water

We first examined a system of colloidal silica particles of nominal diameter 4.82 μm dispersed in deionized water with an estimated background ionic strength of *c*_0_ = 5 μM. The measured electrical potential (zeta potential) in the vicinity of the particle surface, *ζ* ≈ −40 mV, is indicative of a strongly negative surface charge arising from a high density of ionized surface silanol groups. DLVO theory predicts strong interparticle repulsions under these conditions, implying the observation of a randomly dispersed 2D distribution of particles. In marked departure from this intuitive expectation, we noted that the particles spontaneously self-assembled into stable, slowly reorganizing, hexagonally close packed (hcp) clusters characterized by an intersurface separation of the order of *x* ≈ 0.5 μm ≈ 5*κ*^−1^, sharing similarities with previous reports^12,30,31^ (Fig. 1b, middle panels, and Supplementary Video 1). Here *κ*^−1^ is the Debye length which is a measure of the spatial decay rate of the magnitude of electrostatic interactions, and in a solution of monovalent ions can be given in nanometers by $\kappa^{-1} \approx 0.304 /\sqrt{c_{\rm{0}}}$, with *c*_0_ in M. The spatial structure of the clusters, and the presence of large voids between clusters, implies the presence of a strong, long-ranged attractive interaction between particles of like charge. This attraction is counteracted by a repulsion at shorter range, giving rise to a stable pair-potential minimum at comparatively large separations^13^. The measured *g*(*r*) profiles present periodic peaks of diminishing height, reflecting the ordered internal structure of finite-sized clusters—a characteristic signature of attractive interparticle interactions in solution (Fig. 1c, top panel). Simulations of interparticle interactions (snapshots in Fig. 1b, top row) and the corresponding inferred *U*(*x*) agree with calculated *U*_tot_(*x*) curves which capture both the experimentally inferred location of the minima, ${x}_{\min }\approx 2-10{\kappa }^{-1}$, and their depth, $\left|w\right|\approx 4{k}_{{\rm{B}}}T$ (Fig. 1d)^27,28^ (Supplementary Information, section 3.2). Here *κ*_Β_ is Boltzmann’s constant and *T* is the absolute temperature. Experiments performed for *c*_0_ = 5 μM–1 mM on negatively charged silica and carboxylated particles displayed similar trends.

**Extended Data Fig. 1:**
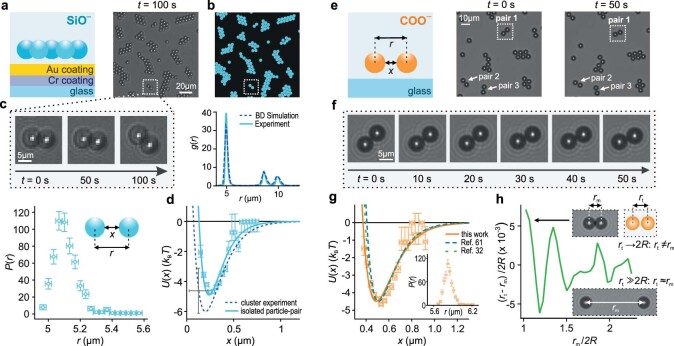
Direct measurement of pair-interaction potentials on isolated pairs of negatively charged particles in water. (**a**) Schematic representation of the experimental system displaying a cluster of SiO_2_ particles of diameter 2*R* = 4.82 μm in suspension above the underlying coverglass. The glass surface is coated with a 5 nm thick chrome (Cr) film followed by a 10 nm thick gold (Au) film in order to suppress any electrostatic contribution from the glass, similar to ref. ^32^ (left). Microscopy image of clusters of silica particles formed above the Au-coated surface, indicating an isolated pair of attracting particles (right). (**b**) Digitised image of clusters (top) accompanied by plot of measured and simulated *g*(*r*) distributions (solid and dashed lines respectively) (bottom). Experiments that display particle clustering often contain isolated, strongly interacting ‘bound pairs’ of particles located several micrometres from other neighbouring particles (white box). (**c**) Time series of snapshots of an isolated pair of interacting particles acquired over a typical period of 100 s (top) and corresponding measured probability density distribution, *P*(*r*), of the interparticle separation, *r* (bottom; see Supplementary Section 4.2). Error bars reflect a spatial localisation accuracy of ≈20 nm in *r* and a shot-noise error estimate on *P*(*r*) (described in Supplementary Sections 1.6 and 4.2). (**d**) *U*(*x*) profiles inferred from both types of measurement: isolated pair *P*(*r*) measurement (symbols and solid fit line) and cluster-based *g*(*r*) (dashed line) measurements (see Supplementary Section 4.2). The *U*(*x*) data corresponding to the *P*(*r*) measurements (blue symbols) are fit to Eq. S6 (Supplementary Fig. 14). The directly measured *U*(*x*) profile for the isolated pair is in good agreement with that inferred from the cluster-formation experiment. (see Supplementary Fig. 14). Grey error bars denote an estimated uncertainty of ±1.5 *k*_B_*T* in the overall process. The horizontal error bar captures the overall uncertainty in measured interparticle separation (arising from the particle size dispersion and localisation error, see Supplementary Section 1.6). (**e**) Schematic of a pair of COOH particles with centre-to-centre distance and intersurface separation denoted by *r* and *x* respectively (left). Experiments on a dilute suspension of particles (approximately factor 2 lower than for the cluster experiments) display a large number of isolated, strongly interacting ‘bound pairs’ of particles located several micrometres from other neighbouring particles as indicated (right). (**f**) Digital video microscopy snapshots of a single pair of interacting COOH particles of diameter 2*R* = 5.29 μm over a period of 50 s. The particle images are characterised by a bright intense region at the centre, surrounded by a dark ring and associated diffraction pattern. (**g**) *P*(*r*) plot of an interacting pair of particles (inset) determined using the TrackNTrace particle tracking code employing the radial symmetry method^52^, that is the particle tracking method used in all experiments in this work. *U*(*x*) data corresponding to measured *P*(*r*) (symbols) accompanied by a fit curve (solid orange line). Error bars on *U*(*x*) values for each of the measured points are derived from a shot-noise estimate (see Supplementary Section 4.2). The solid orange line may be compared with fits to *U*(*x*) data obtained by applying two different corrections to the measured interparticle distances, *r*, according to ref. ^61^ (blue dashed line) and ref. ^32^ (green dashed line). All fits of *U*(*x*) data are to the form of Eq. S6 and details are outlined in Supplementary Fig. 13. Good agreement between the various curves shows that particle-tracking correction methods have a negligible effect on the inferred well depth *w* and *x*_min_ values of the underlying pair interaction potential. (**h**) Plot of the expected difference *r*_t_-*r*_m_ between the true and measured interparticle separation as a function of *r*_m_, for our experimental system, using the method of ref. ^32^ (see Supplementary Section 4.3). The maximum error in the inferred interparticle distance *r*_t_-*r*_m_ = 37 nm is comparable with the localisation accuracy and occurs at an interparticle separation corresponding to the distance of nearest approach *r*_t_ = 2*R*.

Interaction potentials measured for isolated pairs of interacting particles display good agreement with the *w* and *x*_min_ values inferred from the ensemble experiments (Extended Data Fig. 1a–d, Supplementary Fig. 12 and Supplementary Information, section 4.2). This confirms that the observed cluster formation stems from a pair-potential that indeed carries a substantial attractive component, rather than from collective interactions in a system of purely repulsive particles^20^. Furthermore, in contrast to previous suggestions concerning like-charge attraction, we find that the long-ranged attraction is unaffected by the surface properties of the underlying coverglass^15,32,33^ (Extended Data Fig. 1a–d and Supplementary Information, section 4.4) which agrees with previous measurements of attractive pair interactions in bulk solution and with theoretical expectations from the interfacial solvation model^17^. Note that optical image processing artefacts play no role in our measurements of pair-interaction minima^34^ (Extended Data Fig. 1e–h, and Supplementary Information, section 4.3).

Turning to experiments on positively charged aminated silica particles under the same conditions, we found that particles remained randomly dispersed in solution as expected for a purely repulsive interparticle interaction (Fig. 1b, lowest row and Supplementary Video 1). The measured *g*(*r*) profiles were relatively featureless, and categorically devoid of the periodic structure characteristic of ordered clusters (Fig. 1c, lower panel). The corresponding *U*(*x*) profiles imply monotonically decaying repulsions in good agreement with the profiles calculated from the interfacial solvation model (Fig. 1d). The conspicuous absence of a long-ranged minimum in *U*(*x*) for positive particles contrasts with the strong attraction observed for negative particles, and suggests that the solvent is responsible for this striking qualitative departure from the expectation of charge-reversal symmetry in interparticle interactions.

## pH controls the magnitude of the long-ranged force

Next we explored the influence of pH on the interaction between silica particles. Silica surfaces carry a variety of ionizable silanol groups that are characterized by different number densities and highly disparate p*K* values between 2 and 11 which implies a wide range of pH over which the surface ionizable groups may undergo a change in *α*, their extent of ionization^35–37^. We examined cluster formation in silica at a fixed ionic strength with pH varying from 4 to 10 and inferred the value of *w* in each case. While *w* < 0 denotes the depth of the minimum in an attractive pair-potential, we take *w* = 0 for a purely repulsive pair-potential. For silica, we found that although the magnitude of *w* decreased markedly with increasing pH, it remained substantial (|*w*| ≈ 2*k*_B_*T*) even at high pH > 9 (Fig. 2d and Supplementary Video 2). This occurs despite the fact that the electrostatic interaction, ∆*F*_el_, is expected to grow in magnitude with increasing pH, progressively counteracting an attractive interfacial contribution that we expect to simultaneously weaken as $\frac{{\rm{d}}\alpha }{{\rm{d}}{\psi }_{{\rm{s}}}}\to 0$. Here *ψ*_s_ denotes the electrical surface potential of the particle (see Supplementary Information, section 3.1 for details). Importantly, we found that the observations can be qualitatively explained within the interfacial solvation model (Fig. 2a,d, top panels, Supplementary Information, section 3.5 and Supplementary Video 2).

To quantitatively explore the effect of pH, we examined the behaviour of carboxylated melamine resin (COOH) particles where the charge arises from carboxyl groups characterized by a nominal pK ≈ 4.5. We observed a clear maximum |*w*| in the range of p*K* < pH < p*K* +3 , in qualitative agreement with the trend expected for $\frac{{\rm{d}}\alpha }{{\rm{d}}{\psi }_{{\rm{s}}}}$, as given by Supplementary Information equation (9) and discussed further in the Supplementary Information (Fig. 2a,d, middle panels, Supplementary Information, section 3.5 and Supplementary Video 3).

Next, we examined the behaviour of positively charged aminated silica particles carrying a high density of amino groups (NH_2_) of nominal p*K* ≈ 9.5. We did not observe cluster formation in positive particles over the entire range of pH (Fig. 2a, bottom panel and Supplementary Video 4). In fact the *g*(*r*) curves reflected robust interparticle repulsions at large distances that were strongest at low values of pH ≈ 4 (Fig. 2b, bottom panel). Under more alkaline conditions given by 7 < pH < p*K* ≈ 9, the net charge on the particles decreases due to gradual deprotonation of the amino groups. This discharging of the amino groups correlates with a reduction in measured zeta potential and a consequent weakening of the interparticle repulsion (Fig. 2b, bottom panel), but the formation of stable, ordered clusters was never observed (Fig. 2a–c). This range of pH values (7 < pH < 9) may be contrasted with the equivalent pH range of 5–7 for carboxyl particles where we in fact observed the most stable clusters characterized by the deepest minima in interaction energy (largest |*w*|) generated by the long-ranged attractive force. We obtained similar results for silica particles coated with positively charged polyelectrolytes such as polyethyleneimine (PEI) (Supplementary Fig. 16). Thus we find that the measured pH dependence of interparticle interactions—for both signs of particle charge and a range of surface chemistries—carries key signatures of the solvation free-energy contribution, furnishing crucial validation of an interaction mechanism invoking charge-dependent solvent structuring at the solid–liquid interface.

## Sequential switching between attraction and repulsion

**Fig. 3:**
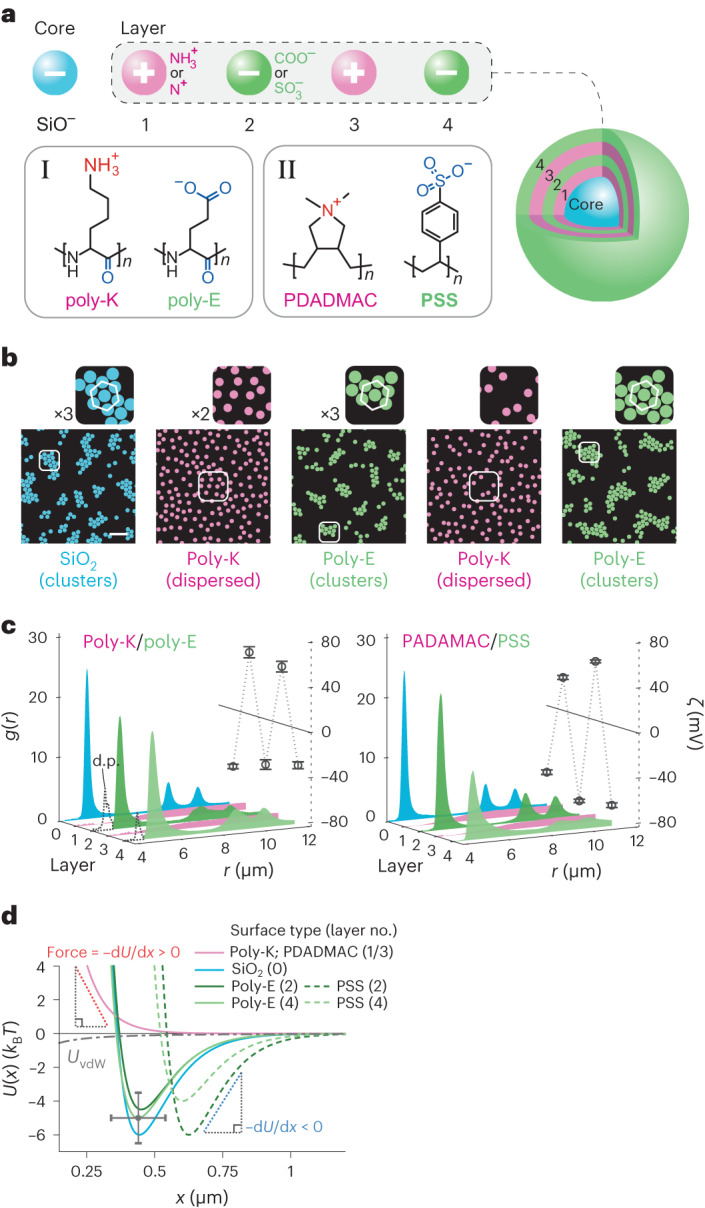
The sign of the long-ranged force depends on the sign of charge of the interacting particles. **a**, Top: schematic depiction of a sequentially coated silica particle displaying four serial layers with alternating sign of coating charge. Bottom: chemical structures of charged polypeptides (poly-K and poly-E) and polyelectrolytes (PDADMAC and PSS) used for layer-by-layer coating of silica particles. **b**, Images of particle suspension structure following each coating procedure using charged polypeptides. Scale bar, 20 μm. See Supplementary Figs. 23 and 25 for polyelectrolyte data, including PEI/PSS coatings, and raw images. **c**, Radial probability density functions *g*(*r*) and measured zeta potentials as a function of coating or layer number for polypeptide (left) and polyelectrolyte coatings (right). Error bars represent mean ± s.d. from three measurements. The alternating sign of zeta-potential values for each new coating layer confirms the change in sign of the particle charge. **d**, *U*(*x*) profiles inferred from measured *g*(*r*) profiles for particles with positively charged (pink curve) and negatively charged coatings (blue and green curves) with layer numbers in parentheses (Supplementary Fig. 23 and Supplementary Tables 9–11). At long range ($x\gtrsim 5{\kappa }^{-1}$), negatively charged particles (charge density *σ* < 0) display an attraction which implies that the interparticle force is attractive, that is, $-\frac{{\rm{d}}U\left(x\right)}{{\rm{d}}x} < 0$, whereas the converse is true for positively charged particles (*σ* > 0) where we infer $-\frac{{\rm{d}}U\left(x\right)}{{\rm{d}}x} > 0$ for all separations, which agrees qualitatively with DLVO theory. All experiments were performed at *c*_0_ = 15 μM. Error bars denote estimated uncertainties of ±100 nm in particle diameter and ±1.5*k*_B_*T* in *w* (Supplementary Fig. 23).

To further probe the influence of surface chemistry on the interparticle interaction we examined the behaviour of negatively charged polystyrene sulfonate (PSS) surfaces. Isolated styrene sulfonic acid groups are highly acidic in free solution (p*K* < −0.5) but are expected to be characterized by p*K* ≈ 3 in the context of a polymer backbone^38^. We produced PSS-coated silica particles using the layer-by-layer polyelectrolyte film deposition method^39^. We first coated silica particles with a layer of positively charged poly(diallyldimethylammonium chloride) (PDADMAC) polymer, which switched the sign of the zeta potential from *ζ* ≈ −50 mV to *ζ* ≈ +40 mV. PDADMAC-coated silica particles in solution no longer formed clusters (Fig. 3). We then further coated PDADMAC-coated silica particles with a layer of PSS, which altered the zeta potential from approximately +40 mV to −70 mV. We found that the system of PSS-coated particles formed stable clusters in solution at pH 5–6 (≈ p*K* +2.5), indicating the reappearance of long-ranged interparticle attractions, similar to uncoated silica particles. We then proceeded to sequentially coat these PSS particles with alternating layers of PDADMAC and PSS, confirming the sign of the particle charge after each coating procedure using zeta potential measurements. We consistently found that the long-ranged interparticle attraction could be switched on and off depending on the sign of charge of the most recent surface coating. Data from four layers of sequential coating are presented in Fig. 3 (Supplementary Video 5). Similar results were obtained with alternating coatings of positively charged PEI and negatively charged PSS (Supplementary Figs. 23 and 25). All polyelectrolyte coating experiments were performed at *c*_0_ = 15 μM (Fig. 3 and Supplementary Fig. 23).

To continue exploring the generality of the ‘like-charge self-assembly’ phenomenon we turned our attention to the examination of the interactions of polypeptide surface coatings on silica particles. Poly-l-lysine hydrobromide (poly-K) and poly-l-glutamic acid sodium salt (poly-E) polypeptides are routinely used as surface coatings, generating positively and negatively charged surfaces on account of their ionized amino (NH_3_^+^; p*K* ≈ 10) and carboxyl (COO^−^; p*K* ≈ 4.5) side-chain groups, respectively. Here again we found that whereas positively charged lysine surfaces repelled as expected (similar to PEI- and PDADMAC-coated particles), poly-E surfaces displayed long-ranged attractions similar to our carboxyl particles (Fig. 3d). Once again we were able to repeatedly alternate between attractions and repulsions in the particle suspension by sequentially coating the particles with poly-K and poly-E layers, a finding that carries strong implications for interactions in proteins.

The above experiments show that negatively charged silica surfaces (p*K* ≈ 2–11), carboxylated melamine resin particles (p*K* ≈ 4.5), polystyrene sulfonate (p*K* ≈ 3) and polyglutamate (p*K* ≈ 4.5) coated polypeptide surfaces suspended in water support long-ranged interparticle attractions when the pH of the solution lies within a range of about 3 units above the p*K* of the ionizable groups. In contrast, positively charged particles display robust repulsions regardless of surface chemistry, and no evidence of a long-ranged attraction over the entire range of pH tested.

## ‘Chemical logic’ in cluster formation

**Fig. 4:**
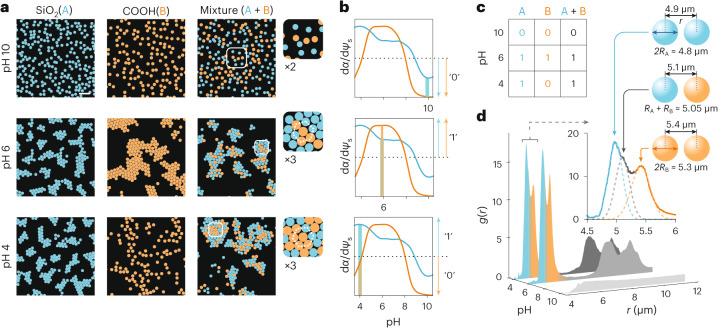
Cluster formation in chemically dissimilar particles. **a**, Structure of suspensions of pure SiO_2_ particles, COOH particles and equimolar mixtures of the two species in solution at pH values 4, 6 and 10, corresponding to salt concentrations *c*_0_ = 0.29, 0.01 and 0.13 mM, respectively. hcp clusters containing both particle species form even under conditions where one of the particle species does not display substantial self-attraction (pH 4 for this system, bottom panels). Scale bar, 20 μm. **b**, Plots of normalized $\frac{{\rm{d}}\alpha }{{\rm{d}}{\psi }_{{\rm{s}}}}$ versus pH for SiO_2_ (blue curve) and COOH (orange curve) from Fig. 2, with a dotted black line indicating a threshold level above which we regard $\frac{{\rm{d}}\alpha }{{\rm{d}}{\psi }_{{\rm{s}}}}$ to be high, taking a binarized value of 1, which is accompanied by the formation of strong and stable same-species clusters as shown in Fig. 2. For low values of $\frac{{\rm{d}}\alpha }{{\rm{d}}{\psi }_{{\rm{s}}}}$ we expect no cluster formation and assign this state and cluster formation outcome a value of 0. **c**, Truth table for the outcome of cluster formation in mixtures (column 3) depending on the binarized values of $\frac{{\rm{d}}\alpha }{{\rm{d}}{\psi }_{{\rm{s}}}}$ for the individual pure species (columns 1 and 2). A value of 1 indicates the formation of mixed clusters containing both particle species, and 0 reflects no cluster formation. **d**, Radial probability distributions *g*(*r*) for the three mixtures in **a**, displaying clear peak signatures of all three expected nearest-neighbour distances in the cross-species hcp clusters, that is, ${r}_{{\rm{AA}}} > {2R}_{{\rm{A}}}$, ${r}_{{\rm{AB}}} > {R}_{{\rm{A}}}+{R}_{{\rm{B}}},{r}_{{\rm{BB}}} > {2R}_{{\rm{B}}}$ (inset).

Next, we performed experiments on mixtures of particles of different surface chemistry and observed the nature of the crystallites formed. Suspensions containing both COOH particles and SiO_2_ particles displayed no cluster formation at high pH values, similar to each of the pure species, as expected (Fig. 4a, top). At pH values where each species is known to form clusters in the pure state (Fig. 2), we found that the mixture also formed hcp clusters composed of both species of particle with signatures in the *g*(*r*) reflecting both particle sizes (Fig. 4a, middle and Fig. 4d). We then performed experiments at pH ≈ 4 where pure COOH particles do not form clusters, but SiO_2_ particles do cluster. Interestingly, we found that rather than exclude the non-cluster-forming COOH species, the system displayed stable hcp clusters containing both types of particles (Fig. 4a, bottom and Supplementary Video 6). All these observations can be explained within the proposed interfacial solvation model of interparticle interactions (Supplementary Information, section 3.6). Specifically, it appears sufficient for one species of particle to be in the charge-regulating regime $\left(\frac{{\rm{d}}\alpha }{{\rm{d}}{\psi }_{{\rm{s}}}}\ne 0\right)$ for an attraction between chemically dissimilar like-charged particles to manifest (Fig. 4b,c).

Thus we show that similar or identical chemistry is not a requirement for interparticle attraction, but that the electrical charge-state of the groups underpins a force with a general and tunable character that holds in a broad sense. Simply put, chemical properties of the particle may enter the physics of the interaction via the p*K* of the ionizable groups. These observations could carry implications for the biological phase separation problem, for example, where strongly acidic RNA molecules participate in cluster formation in mixtures involving other charged molecular species such as proteins^40,41^. To emphasize the connection with the biomolecular condensation problem, the silica particles in the clusters (Fig. 4a, bottom) may be seen to act as the ‘scaffold’, while the carboxyl particles act as ‘clients’^42^.

## Negative self-attraction in water changes sign in alcohols

**Fig. 5:**
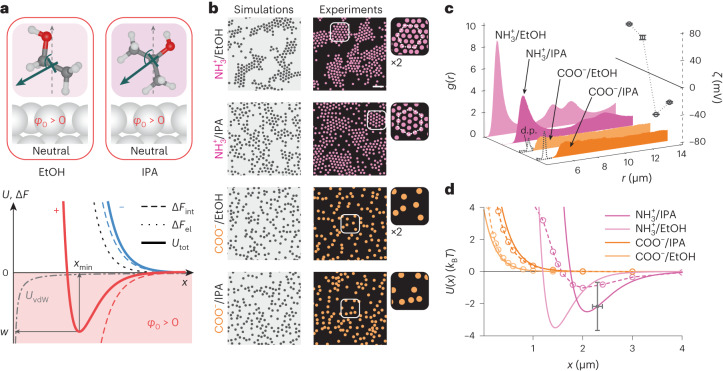
Negative self-attraction in water switches to positive self-attraction in alcohols. **a**, Top: MD simulations of ethanol and IPA at an uncharged surface show that these molecules orient on average with their methyl groups pointing slightly towards the interface and their electronegative oxygen atoms pointing towards the bulk, giving an interfacial potential *φ*_0_ > 0. Unlike water, the average interfacial molecular dipole moment (green arrow) points towards the surface. Bottom: various contributions to the total interaction free energy for positive (red lines) and negative (blue lines) particles: electrostatic repulsion, ∆*F*_el_ (black dotted line; applies to both signs of charge); interfacial solvation contribution, ∆*F*_int_ (dashed line, different for positive and negative); total interaction energy, ${U}_{{\rm{tot}}}={\Delta F}_{{\rm{el}}}+{\Delta F}_{\mathrm{int}}$ (solid lines); and the vdW contribution, *U*_vdW_ (dashed-dotted grey line; applies to both cases). When *φ*_0_ > 0, positively charged particles may display a minimum in *U*_tot_ at an interparticle separation *x*_min_ (solid red line), whereas negatively charged particles repel monotonically, displaying no minimum (solid blue line) (Supplementary Information, section 3.1). **b**, Right: experiments show that positively charged aminated silica particles (pink) form hcp clusters, whereas negatively charged carboxylated particles (orange) do not form clusters in alcohols. Scale bar, 20 μm. See Supplementary Fig. 25 for raw images. Left: BD simulations with appropriate input pair-interaction potentials recover the experimentally observed suspension structure. **c**, Measured radial probability density functions, *g*(*r*), for positively charged particles (shades of pink) and negatively charged particles (shades of orange) in alcohols, including measured zeta potentials (circular symbols) for each case. Error bars denote ±s.d for three measurements. Experiments correspond to measured values of *c*_0_ ≈ 0.4 μM (see Supplementary Table 12 for details). **d**, Pair interaction potentials inferred for the experimental data from BD simulations (solid lines) for negative particles (orange) and positive particles (pink) (see Supplementary Fig. 24 and Supplementary Table 12 for details). Calculated *U*_tot_(*x*) curves as described in ref. ^27^ and Supplementary Information, sections 3.2 and 3.3 (dashed lines with symbols; see Supplementary Table 1 for parameter values). Error bars denote estimated uncertainties of ±100 nm in particle diameter and ±1.5*k*_B_*T* in *w* (Supplementary Fig. 24).

The question then naturally arises as to whether the type of charge asymmetry observed for the interparticle interaction in water could be altered in a different solvent on account of differences in molecular orientational behaviour at an interface. We first confirmed that positively charged amine-derivatized silica particles suspended in ethanol and isopropanol (IPA) displayed positive zeta potentials (Fig. 5c). We recall that these particles remained well dispersed when suspended in water (Figs. 1 and 2). In ethanol and IPA however, we found that positively charged particles formed stable crystalline clusters, displaying *g*(*r*) distributions reminiscent of attractive interactions generally encountered for negatively charged silica particles in water (Fig. 5b,c and Supplementary Video 7). Negatively charged carboxylated particles, on the other hand, remained well dispersed in both alcohols, did not form clusters and qualitatively echoed observations on positively charged particles in water.

Molecular simulations of ethanol and IPA indeed show that the normal component of the average molecular dipole can be inverted with respect to that of molecular water at a neutral surface; that is, interfacial molecules orient such that their comparatively hydrophobic methyl (–CH_3_) groups point towards a neutral surface (Fig. 5a and Supplementary Fig. 8b; Supplementary Information, section 2.2)^43^. This molecular orientation gives an interfacial solvation potential at zero surface charge that has a positive sign, that is, *φ*_0_ > 0, which would suggest attraction between positive particles in our model. Attempts to experimentally probe the interfacial electrical potential have indeed suggested similar orientational behaviour and an inversion in sign of *φ*_0_ for alcohols at interfaces compared to water^44–46^. Furthermore, the experimentally inferred *U*(*x*) curves are qualitatively captured by calculations of *U*_tot_(*x*), as shown in Fig. 5d, yielding physically plausible model parameters (Supplementary Video 7, Supplementary Information, section 3.3 and Supplementary Table 1).

## Interfacial solvation and biomolecular condensation

**Fig. 6:**
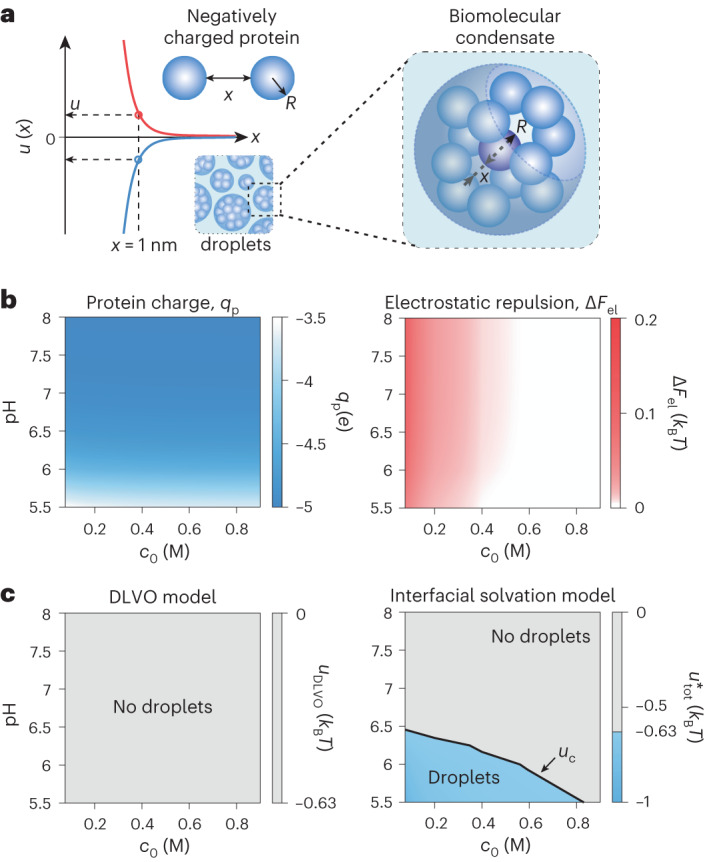
The charge-dependent solvation force can drive the formation of biomolecular condensates. **a**, Schematic representation of biomolecular condensate droplets formed from protein molecules depicted as spheres of radius *R* = 2.5 nm carrying a negative electrical charge equivalent to a net surplus of five glutamic acid groups. Blue and red lines present two possible, qualitatively different interaction potentials *u*(*x*) (for example, ${u}_{{\rm{DLVO}}}\left(x\right)$ or ${u}_{{\rm{tot}}}^{* }\left(x\right)$) for a pair of interacting molecules where *u* is the value of interaction energy at an intersurface separation of *x* = 1 nm in either scenario. **b**, Left: the calculated molecular charge attains its maximum value of *q*_p_ = −5*e* at pH > 6.5 and remains substantially negative over a broad range of pH values. Right: calculation of the electrostatic interaction energy Δ*F*_el_ (*x* = 1 nm) suggests a net repulsive interaction between two molecules for the relevant pH and *c*_0_ range. **c**, Left: applying a pairwise interaction energy threshold for droplet formation of $u\lesssim {u}_{c}=-0.63{k}_{{\rm{B}}}T$ to the calculated value of ${u}_{{\rm{DLVO}}}={\Delta F}_{{\rm{el}}}+{u}_{{\rm{vdW}}}$ we obtain no indication of phase separation from a DLVO perspective. Right: inclusion of the solvation free-energy contribution in the interaction potential ${u}_{{\rm{tot}}}^{* }={{u}_{{\rm{DLVO}}}+\Delta F}_{\mathrm{int}}$ yields a coexistence curve (black line) which displays pH and salt concentration dependence of droplet formation similar to the experimental observations (see Supplementary Information, section 3.7 for details).

Finally, inspired by the spontaneous formation of tuneable clusters in like-charged microspheres in solution, we examine the implications of this long-ranged solvation force for the formation of reversible condensates at the molecular scale under physiological conditions, broadly encountered, for example, in the liquid–liquid phase-segregation problem in biology. We constructed a simple model of a protein as a sphere carrying surface-ionizable groups exposed to the solvent (Fig. 6a) and examined the pair-interaction energy, *u*(*x*), of two spherical, charged molecules carrying five ionizable glutamic acid groups (p*K* = 4.5), immersed in an electrolyte containing monovalent salt at a concentration in the range *c*_0_ = 10 mM–1 M in the pH range 5.5–8 (Fig. 6, Supplementary Fig. 10 and Supplementary Information, section 3.7). We analysed the sign and magnitude of the total interaction potential, ${u}_{{\rm{tot}}}^{* }\left(x\right)$, given by Supplementary Information equation (5), at a nominal intersurface distance of *x* = 1 nm between the molecules.

We noted that the interparticle electrostatic repulsion, reflected in Δ*F*_el_, is expected to be substantial over the range of pH and salt concentration considered since the molecules carry substantial net charge (of the same sign) (Fig. 6b). Interestingly, upon adding the interfacial contribution to the total free energy we found that the interaction could be expected to turn distinctly attractive (that is, ${u}_{{\rm{tot}}}^{* } < 0$ relative to infinite separation), particularly at lower ionic strength and at pH values higher than the p*K* of the charged groups (Supplementary Fig. 10 and Supplementary Information, section 3.7). We then estimated the profile of a coexistence curve demarcating parts of the parameter space where phase separation may occur from regions where the molecules remain well dispersed. We found that the interfacial model suggests the counterintuitive formation of condensates of like-charged molecules at salt concentrations up to *c*_0_ ∼ 0.5 M and at pH values as high as ≈6.5 (Fig. 6c, right panel and Supplementary Information, section 3.7). Such behaviour has in fact been reported for proteins carrying net negative charge^42,47^, for example, the negatively charged wild-type yeast protein Sup35 which carries a net excess of six acidic amino acids (aspartic and glutamic acids) over the number of basic side chains (lysine and arginine) in the domain that controls its phase-segregation response (Supplementary Fig. 10l)^47^. A DLVO-type model of the same interaction, however, would not capture the experimental trends reported for Sup35 (Fig. 6c, left panel and Supplementary Information, section 3.7).

## Conclusion

This study provides compelling evidence for a profoundly different view of interparticle and intermolecular interactions with general implications for self-assembly and clustering of matter in solution, relevant even, for example, to the origin of life^48^. The concerted action of electrical charge at an interface and the local interfacial solvation structure can give rise to an ‘electrosolvation force’ between objects in solution. As a rule of thumb, interparticle attraction between identical, nominally repulsive particles may be expected when the sign of the interfacial electrical potential, *φ*_0_, in a particular solvent matches that of the sign of charge of the particle. The observations call for a re-evaluation of archetypal notions in biomolecular interactions, for example, that negatively charged poly-E stretches in proteins repel unequivocally in solution, which does not necessarily hold depending on the pH in solution (Fig. 2, middle panels). Like-charged molecules in solution may in fact experience a counterintuitive strong and long-ranged attraction, even under physiological conditions. These results have further ramifications for interactions in solvent mixtures, and in solutions containing additives such as zwitterions, osmolytes, polyols or other overall electrically neutral molecules that are not expected to strongly influence the electrostatic interaction per se^49^. Net-neutral molecular species in solution may alter the interfacial solvation structure due to the pure electrolyte and thus modify the total interaction between charged particles. The concept invoked here for longer-ranged interparticle and intermolecular interactions may well be found to hold for intramolecular interactions and conformational changes associated with nanometre-scale processes such as biomolecular folding. Finally, our findings provide evidence for the ability to probe the sign and magnitude of the interfacial electrical potential due to the solvent, previously believed to be immeasurable^44,45,50^.

## Methods

### Experimental methods

#### Preparation of particle suspensions for microscopy measurements

In our experiments we study the behaviour of three distinct types of microspheres with different surface chemistries. The three main classes of particle involve SiO_2_ particles (Bangs Laboratories), amine-derivatized silica particles (NH_2_–SiO_2_, referred to as ‘NH_2_’ or ‘${\rm{N}}{{\rm{H}}}_{3}^{+}$’ in the text) (microParticles) with an estimated NH_2_ group content of >30 µmol g^−1^, and carboxylated melamine formaldehyde (COOH–MF, referred to as ‘COOH’ or ‘COO^−^’ in the text) particles (microParticles) with a carboxyl group content of 400 µmol g^−1^. The particle size distributions provided by the manufacturer are shown in Supplementary Fig. 1.

For experiments on SiO_2_ and COOH particles in aqueous solution, particles were first rinsed (centrifuged and resuspended) in deionized water. Next, they were incubated in 5 mM NaOH (99.99%, Alfa Aesar) solution for 10 min. Following this, they were centrifuged and resuspended in aqueous electrolyte of the required ionic strength around six times until the measured electrical conductivity of the supernatant solution converged to that of the pure electrolyte. Note that in general NaOH treatment is not essential, and overnight exposure to deionized water with subsequent rinsing in deionized water is an equally effective treatment prior to clustering experiments (Supplementary Fig. 11). However, NaOH pretreatment was necessary to observe strong clustering in COOH particles.

NH_2_–SiO_2_ particles were first rinsed in deionized water and then resuspended multiple times in aqueous electrolyte solution until the measured supernatant conductivity converged to that of the pure electrolyte. The suspension was further sonicated for cases in which a large population of ‘sticking’ particles were observed. The presence of stuck particles in the experimental data gives rise to small ‘dimer’ peaks at interparticle separations 2*R* in the measured *g*(*r*) which cannot be entirely eliminated (indicated as ‘d.p.’ in Figs. 2b, 3c and 5c). In experiments on mixtures of SiO_2_ and COOH particles (Fig. 4), the two types of particles were initially mixed at a 1:1 ratio, then incubated in 10 mM NaOH solution for 10 min. Thereafter the procedures were the same as described above.

For experiments on colloidal dispersions in alcohols, NH_2_–SiO_2_ particles were first centrifuged and resuspended in deionized water, followed by resuspension in either ethanol (≥99.8%, Sigma-Aldrich) or IPA (≥99.5%, Sigma-Aldrich). The process of centrifugation and resuspension was repeated multiple times until the value of the supernatant conductivity converged to that measured for the pure alcohol. COOH particles were first centrifuged and resuspended in deionized water, followed by resuspension in ethanol, and final resuspension in either ethanol or IPA for measurements.

#### Preparation and characterization of electrolyte solutions

For experiments examining the dependence of interparticle interactions on the ionic strength of the electrolyte (Fig. 1), various concentrations of NaCl (99.998%, Alfa Aesar) solution were prepared in deionized water; the measured conductivity of these solutions corresponds to a background concentration of monovalent ions of *c*_0_ ≈ 5 μM. The ionic strength of the various electrolyte solutions in our experiments was determined from measurements of electrical conductivity, *s*, performed with a conductivity meter (inoLab Cond 7110). A calibration curve of standard solutions was used for this purpose (Supplementary Fig. 2).

To convert the measured electrical conductivity to a background salt concentration in alcohols, we used the same calibration relationship as for aqueous electrolytes as shown in Supplementary Fig. 2, but corrected the inferred concentrations for the viscosity of the alcohol as suggested in previous work^51^ (the viscosity values used for ethanol and IPA were 1.1 cP and 2.4 cP, respectively, see Supplementary Information, section 1.2).

In experiments exploring the pH dependence of interparticle interactions (Fig. 2), the pH of the electrolyte was adjusted to the desired value by adding either HCl (99.999%, Alfa Aesar) or a Tris buffer (≥99.9%, Carl Roth), to deionised water. Addition of acid or buffering agent to deionized water raised the conductivity of the solution to a value between 1 and 30 μS cm^−1^ (0.01–0.25 mM) depending on the target pH value. For experiments performed at variable pH, ionic strength in the electrolyte was maintained constant (to within ±0.02 mM) across the entire range of pH in a given experimental series via the addition of a variable amount of NaCl. The pH value of the aqueous solution was taken as the mean value of three consecutive measurements using a pH meter (Horiba PH-33). The pH of pure alcohol samples was inferred by extrapolation of pH values measured for water–alcohol mixtures (Supplementary Fig. 2b).

#### Layer-by-layer coating of silica particles with polypeptides and polyelectrolytes

In the experiments presented in Fig. 3, we used alternating coatings of positively and negatively charged polyelectrolytes on plain SiO_2_ particles. Coatings were applied in the following pairs of combinations of positively and negatively charged polyelectrolytes: poly-K (*M*_r_, ≥300,000; Sigma) and poly-E (*M*_r_, 50,000–100,000; Sigma); PDADMAC (*M*_r_, 200,000–350,000; Aldrich) and PSS (*M*_r_, ∼70,000; Aldrich), and finally, PEI (*M*_r_, ∼750,000; Sigma) and PSS.

To coat the particles, plain SiO_2_ particles were first centrifuged and resuspended in deionized water, followed by incubation in 5 mM NaOH solution for 10 min, resuspension in deionized water, and repetition of the resuspension process until the supernatant conductivity converged to that of deionized water. The rinsed particles were then incubated in the polyelectrolyte solution at 0.1% w/v for 20 min with occasional vortexing to improve mixing. The coated particles were centrifuged and resuspended in deionized water to remove any excess polymer and the resuspension procedure repeated until the conductivity of the supernatant no longer changed. Subsequent layers of polymer coatings were applied by repeating the coating procedure described above with the corresponding oppositely charged polyelectrolyte. The sign of the surface charge of each coating layer was confirmed by zeta-potential measurements (Zetasizer Nano Z, Malvern Panalytical).

#### Cuvette preparation and sample loading

We used a glass cuvette with a polished flat-well of 1 mm depth (20/C/G/1, Starna Scientific), as shown in Supplementary Fig. 3b, for all video microscopy measurements. The cuvette was cleaned with piranha solution (3:1 mixture of concentrated sulfuric acid and 30 wt% hydrogen peroxide solution) and then rinsed thoroughly with deionized water. A glass cuvette naturally provides a negatively charged surface in water, ethanol and IPA for experiments with negatively charged particles. For experiments with positively charged particles, the entire cuvette was coated with 1% w/v PEI solution, rinsed and dried under nitrogen to provide a thin layer of positively charged polymer coating. To load the cuvette, the prepared particle solution was carefully pipetted into the well and sealed with the cover slide such that the device was free of air bubbles and held together by capillary force.

#### Microscopy

The optical microscope was constructed using a 470 nm light-emitting diode (LED) (M470L4, ThorLabs), a 10× objective (Olympus UPlanSApo) and a charge-coupled device camera (DCU223M, ThorLabs) for recording images (Supplementary Fig. 3). The sample holder was placed onto a carefully balanced pitch and roll platform (AMA027, ThorLabs). Following complete settling of particles in suspension to a plane near the bottom surface of the cuvette, which typically takes about 2 min, the focus was adjusted such that a clear intensity maximum was observed for all particles. All measurements were performed after complete settling. The intensity of the LED was adjusted such that the intensity maxima of illuminated particles did not exceed the saturation value of the camera, enabling accurate particle localization.

#### Video recording and data processing

Sequential images of the 2D suspension of colloidal particles were taken with ThorCam software at a constant frame rate of 5, 10 or 30 frames per second for 150–500 frames using an exposure time of ≈ 0.5 ms. The images were processed based on the radial symmetry method using the TrackNTrace particle-tracking framework, where the particle centre maximum is detected^52,53^. The localization precision for a static SiO_2_ particle during a 100 s measurement at an exposure time of ≈ 0.5 ms was found to be <20 nm, as shown in Supplementary Fig. 4. In the analysis of experimental images, coordinates of all particle centres were extracted from the recorded frames, and the radial distribution function curve *g*(*r*) calculated and averaged over all images. To clearly distinguish between experiments on particles with different signs of particle charge in three different solvents, the recorded images were digitized and false-coloured (Supplemenetary Information, section 1.6 and Supplementary Fig. 3). The average particle detection efficiency over all experiments was >98%.

### Simulation methods

#### BD simulations of interparticle interactions

We performed BD simulations of a 2D distribution of spheres interacting via an appropriately chosen input potential using the BROWNIAN package in the LAMMPS software^54^. We inferred the required pair-interaction potentials, *U*(*x*), underpinning the experimental data by varying the input potential to the BD simulations. We thus generated simulated radial probability distribution functions, *g*(*r*)s, that matched the experimentally measurements. Validation and further discussion of the BD simulation set-up and approach are provided in Supplementary Information, section 2.1. Example input and necessary simulation files are provided in our Figshare repository, available at: 10.6084/m9.figshare.c.6132003.

We assumed a pairwise interaction potential *U*(*x*) of the form: $U(x)=A{e}^{-{\kappa }_{1}x}+B{e}^{-{\kappa }_{2}x}+{U}_{{\rm{vdW}}}$. Here the first term represents the overall repulsive electrostatic free energy of interaction, $\varDelta {F}_{{\rm{el}}}(x)=A\,\exp (-{\kappa }_{1}x)$, with *A* > 0 and the second term, $\varDelta {F}_{{\rm{int}}}(x)=B\,\exp (-{\kappa }_{2}x)$, denotes the free-energy contribution arising from interfacial solvation^2^. Note that ${\kappa }_{2} < {\kappa }_{1}\approx \kappa$. The third term represents the vdW attraction between silica particles in solution, for which we have used the expression in ref. ^55^.

BD simulations were performed by taking into account the experimentally determined polydispersity in particle size as shown in Supplementary Fig. 1. This implies that at the simulation level a variable particle radius is taken into account to the lowest level of approximation (that is, the interaction potential remains fixed and independent of the size of the particles, which would not be true in practice). Using a value of the Hamaker constant *A*_H_ = 2.4 zJ we found that *U*_vdW_ made a small contribution (≈ −0.5*k*_B_*T*) to the total interaction energy at large separations, *x* ≥ 0.2 μm, that is, for the majority of experiments in this work^56,57^. However, for experiments at higher salt concentrations (*c*_0_ ≈ 1 mM; Fig. 1d), the vdW interaction can make a more substantial contribution (≈ −1*k*_B_*T*) to the interaction at separations *x* ≈ 0.1 μm, and for this reason it was included in our expression for *U*(*x*) when modelling these measurements.

The experimentally measured *g*(*r*) curve provides an estimate of the the location of the minimum in the pair potential *x*_min_. In Supplementary Information equation (6), the screening length ${\kappa }_{1}^{-1}={\kappa}^{-1}$, which is known from the measured salt concentration. We then use a trial value of the interaction free energy at the minimum, $U\left({x}_{\min }\right)=w < 0$, to obtain initial values for the parameters *A* and *B* as inputs for the pair-interaction potential, *U*(*x*), using Supplementary Information equation (4), where we have taken *κ*_2_/*κ*_1_ ≈ 0.95, as suggested in ref. ^28^. We note, however, that this ratio is not a strict requirement and that we may also treat it as a free parameter which yields an alternate set of parameters *A*, *B*, *κ*_1_ and *κ*_2_ that can provide equally good qualitative agreement with the experimental data (see, for example, Supplementary Table 5).

Particle configurations for the BD simulations were initialized via random particle placement in a 200 × 200 μm^2^ simulation box that reproduced the experimental particle density (≈ 0.008 particles μm^−^^2^). The polydispersity of the simulated colloids was drawn from the manufacturer’s size distribution for each particle type, as shown in Supplementary Fig. 1. Periodic boundaries were applied in the *x,y* dimensions while the *z* dimension was held finite. The *z* coordinate of the colloids were fixed at a constant height throughout the simulation, ensuring a 2D system and thus mimicking the experiment. BD simulations were performed assuming that the interactions between the particles may be regarded as pairwise additive (discussed further in the main text and in Supplementary Information, section 3.4)

Convergence of the potential energy per particle in our BD simulations was monitored over time (Supplementary Fig. 5). Particle positions used for the calculation of the final simulated *g*(*r*)s were collected once the value of the potential energy reached a stationary value, after ≈ 30 min of simulation time in a run involving a strongly attractive *U*(*x*) of a well depth of several *k*_B_*T* (Supplementary Fig. 5). Agreement between the simulated and the experimental *g*(*r*)s was assessed for a trial input pair-interaction potential *U*(*x*) and the value of the well depth *w* adjusted in subsequent BD simulations if required, to obtain a final simulated best match with the experimental data. This procedure permitted us to infer the functional form of an underlying pair-interaction potential *U*(*x*) capable of capturing the experimentally measured *g*(*r*).

#### Molecular dynamics simulations of alcohols at interfaces

The excess electrical potential due to the orientation of solvent molecules at an interface, *φ*_0_ or *φ*_int_, is required as an input to the interfacial solvation model to calculate theoretical *U*_tot_(*x*) curves (Supplementary Information, section 3.2). To estimate *φ*_int_(*σ*) as a function of surface electrical charge density, *σ*, we performed molecular dynamics (MD) simulations with the GROMACS MD code^58^. We examined the behaviour of a solvent phase in contact with a model surface composed of oxygen atoms in a parallel-plate capacitor set-up, as described extensively in previous work^27,29^. Example input files, force-field parameters and code for the analysis of the simulations performed in this study are available in our Figshare repository: 10.6084/m9.figshare.c.6132003.

Prior to running MD simulations in the capacitor set-up, we first ran preliminary simulations of a box of 7,500 IPA molecules, without the capacitor wall atoms, under constant pressure, maintained with the Parrinello–Rahman pressure-coupling method. The length of the box in *z* was allowed to fluctuate while keeping the *x*,*y* dimensions fixed to those of the capacitor walls of fixed area. This equilibrated slab of solvent was then sandwiched between capacitor plates comprised of positionally restrained oxygen atoms that only support Lennard–Jones interactions (Supplementary Fig. 7). In our simulations, IPA molecules were parametrized with the CHARMM36 force field^59^. As in previous work, the plates are ≈ 10×10 nm^2^ in area and are separated by ≈ 8 nm of solvent medium in the *z* direction^27,29^. This ensures that any oscillations in the solvent density or dipole moment profiles attain bulk-like properties at a location *z*_mid_ in the middle of the capacitor. A subset of the atoms belonging to the first layer in each wall (in direct contact with the solvent) was randomly assigned a positive (left plate) or a negative charge (right plate) to generate an electric field of specific strength in the box while maintaining electroneutrality within the box. The capacitor system simultaneously yields estimates of *φ*_int_(*σ*) for both positive and negative values of *σ* and provides a well-defined system for comparing solvation at a macroscopic surface with a continuum electrostatics model.

Next, a second round of equilibration was carried out for the entire capacitor system, including the capacitor walls, and consisted of a short NVT run with a velocity-rescaling thermostat, followed by 500 ps in an NPT ensemble where only the *z* dimension of the box was allowed to fluctuate, keeping the the *x*,*y* dimensions fixed. This ensured that solvent molecules were maintained at the correct density throughout the simulation box. Following this procedure, production MD runs of 20 ns duration were performed in an NVT ensemble with trajectory frames written every 20 ps. The particle mesh Ewald method was used to evaluate the long-range electrostatic interactions using a 1 Å grid spacing and a short-range cut-off of 12 Å. The Lennard–Jones interactions were smoothed over the range of 10–12 Å using the force-based switching function. We scaled the *z* dimension of the box by a factor 2 for Ewald summation only and applied the 3dc correction of Yeh and Berkowitz to remove artificial polarization induced by neighbouring image dipoles^60^. The orientation of solvent molecules as a function of distance from the walls was analysed to yield *φ*_int_(*σ*) values that were used in the calculation of the interfacial free energy term, Δ*F*_int_, as previously described^27–29^.

## Online content

Any methods, additional references, Nature Portfolio reporting summaries, source data, extended data, supplementary information, acknowledgements, peer review information; details of author contributions and competing interests; and statements of data and code availability are available at 10.1038/s41565-024-01621-5.

### Supplementary information

Supplementary Information Supplementary experimental methods, simulation methods, calculation methods and discussion, results, Figs. 1–25 and Tables 1–12.

Supplementary Video 1 Ionic strength dependence of charge-reversal asymmetry in interparticle interactions for negatively charged SiO_2_ and positively charged NH_2_–SiO_2_ particles in aqueous solution.

Supplementary Video 2 pH dependence of cluster formation in negatively charged SiO_2_ particles in aqueous solution.

Supplementary Video 3 pH dependence of cluster formation in negatively charged COOH particles in aqueous solution.

Supplementary Video 4 pH dependence of interparticle interactions in positively charged NH_2_–SiO_2_ particles in aqueous solution.

Supplementary Video 5 Controlling interparticle interactions in aqueous solution by switching the sign of particle charge using layer-by-layer coating with polypeptides (poly-K/poly-E) and polyelectrolytes (PDADMAC/PSS).

Supplementary Video 6 Cluster formation in chemically dissimilar particles (SiO_2_ and COOH) at pH 4, 6 and 10.

Supplementary Video 7 Charge-reversal asymmetry of interparticle interactions in alcohols: experiments on negatively charged COOH particles and positively charged NH_2_–SiO_2_ particles in ethanol and isopropanol.

## Data Availability

Raw experimental data are available from our Figshare repository at: 10.6084/m9.figshare.c.6132003.

## Code availability

Simulation files and code used to analyse the experimental and simulated data are available from our Figshare repository at: 10.6084/m9.figshare.c.6132003.
