# Supplementary material for: A charge-dependent long-ranged force drives tailored assembly of matter in solution
Source: Nat Nanotechnol. 2024 Mar 1;19(4):485–93. doi: 10.1038/s41565-024-01621-5 (PMC11026162; doi:10.1038/s41565-024-01621-5)
Supplement: Supplementary file 1 — Supplementary experimental methods, simulation methods, calculation methods and discussion, results, Figs. 1–25 and Tables 1–12. [file 41565_2024_1621_MOESM1_ESM.pdf]

nature nanotechnology

Article

https://doi.org/10.1038/s41565-024-01621-5

A charge-dependent long-ranged force drives tailored assembly of matter in solution

In the format provided by the authors and unedited

Table of Contents

S1 Supplementary experimental methods *5*

S1.1 Preparation of particle suspensions for microscopy measurements 5

S1.2 Electrolyte solution preparation and measurements of solvent properties 6

S1.3 Layer-by-layer coating of silica particles with polypeptides and polyelectrolytes 8

S1.4 Cuvette preparation and sample loading 8

S1.5 Microscopy 9

S1.6 Video Recording and data processing 10

S2 Supplementary simulation methods *12*

S2.1 Brownian Dynamics (BD) simulations of interparticle interactions 12

S2.2 Molecular Dynamics (MD) simulations of alcohols at interfaces 16

S3 Supplementary calculation methods *19*

S3.1 A brief overview of the interfacial solvation model of interparticle interactions 19

S3.2 Summary of the approach to calculating the total interparticle interaction 22

S3.3 Discussion on parameter values used in the theoretical modelling of the experimentally inferred interaction potentials 26

S3.4 Considerations in comparing the results of pair-interaction-based theory and simulation procedures with an experiment involving an ensemble of interacting particles 29

Pairwise additivity of interaction energies is a simplifying assumption 29

Experimental variability in parameters of interest 30

Scope for improvement of the present theoretical model 30

S3.5 Analysing the p*H* response of particle cluster formation 32

Silica particles 33

Carboxyl particles (-COOH groups) 34

Amino-silica particles (-NH_2 groups) 35

S3.6 Cluster formation in mixtures of chemically dissimilar particles 36

S3.7 Protein pair-interaction modelling and calculation of a protein phase separation response as a function of p*H* and salt concentration 38

S4 Supplementary Results *45*

S4.1 Interactions in silica particles in water without prior treatment with NaOH 45

S4.2 Direct measurements of interaction potentials for pairs of isolated particles 45

S4.3 Optical artifacts in particle tracking do not influence the measurements 47

S4.4 The long-range attraction is not influenced by surface charge on the neighbouring coverglass surface 49

S4.5 Ionic strength dependence of cluster formation for COOH particles in aqueous solution 50

S4.6 pH dependence of the interactions of PEI-coated silica particles 51

S4.7 Ionic-strength dependent swelling of positively charged polyelectrolyte coatings detected in $g(r)$ measurements 52

S5 Data Processing & Analysis 55

S5.1 Matching experimentally measured $g(r)$s with those from BD simulations 55

S5.2 Overview of all experimental snapshots and corresponding digitized images 62

S6 Supplementary Tables 65

Table S1. Parameter values used in PB-interfacial solvation model calculations of $U_{tot}(x)$ 65

Table S2. BD simulation parameter values used to model experiments that examine the salt concentration dependence of cluster formation for SiO_2 particles in water 66

Table S3. BD simulation parameter values used to model experiments that examine the salt concentration dependence of interparticle interactions for NH2-SiO_2 particles in water 67

Table S4. BD simulation parameter values used to model experiments that examine the p*H* dependence of cluster formation for SiO_2 particles in water 68

Table S5. Alternative set of parameters for the experiments varying p*H* for SiO_2 particles in water 69

Table S6. BD simulation parameter values used to model experiments that examine the p*H* dependence of cluster formation for COOH particles in water 70

Table S7. Alternative set of parameters for the experiments varying the p*H* for COOH particles in water 71

Table S8. BD simulation parameter values used to model experiments that examine the p*H* dependence of interparticle interactions for NH2-SiO_2 particles in water 72

Table S9. BD simulation parameter values used to model experiments that examine the dependence of cluster formation on the charge of the polypeptide coating layer (poly-K/poly-E) displayed in Fig. 3c 73

Table S10. BD simulation parameter values used to model experiments that examine the dependence of cluster formation on the charge of the polyelectrolyte coating layer (PDADMAC/PSS) displayed in Fig. 3c 74

Table S11. BD simulation parameter values used to model experiments that examine the dependence of cluster formation on the charge of the polyelectrolyte coating layer (PEI/PSS) 75

Table S12. BD simulation parameter values used to model experiments that examine the dependence of cluster formation on the sign of the charge of the particle in ethanol and isopropanol 76

S7 References 77

List of Figures

Fig. S1. Probability distributions of particle diameter for each particle type studied in experiments
Fig. S2. Calibration curves underlying salt concentration and p*H* measurements
Fig. S3. Experimental set-up for measurements on 2-d colloidal dispersions
Fig. S4. Spatial localization accuracy of a single immobilized SiO_2 particle
Fig. S5. Convergence of the potential energy per particle, *PE*/*N*, over the course of a BD simulation
Fig. S6. Influence of the particle polydispersity on the $g(r)$ inferred from a BD simulation
Fig. S7. MD simulations of isopropanol in a capacitor setup: calculation of $\varphi_{\mathrm{int}}(\sigma)$ for an alcohol at an interface
Fig. S8. Schematic depiction of the qualitative average orientation of a solvent molecule at the solid-liquid interface
Fig. S9. ζ potentials for SiO_2 and COOH particles
Fig. S10. Plots of the various contributions to the pair interaction energy $u^*_{\mathrm{tot}}(x)$ for a pair of negatively charged protein molecules, calculated as a function of p*H* and salt concentration c_0 at an intermolecular separation x = 1 nm
Fig. S11. Interaction of silica particles in deionised (DI) water prepared without prior NaOH treatment
Fig. S12. Direct measurement of pair-interaction potentials for COOH particles
Fig. S13. Application of particle tracking corrections and their effect on the measured pair interaction potentials $U(x)$
Fig. S14. Examining the effect of coating the underlying coverglass of the experimental cell with thin metal films
Fig. S15. Ionic strength dependence of the interactions of COOH particles in aqueous electrolyte at p*H*=5.8
Fig. S16. p*H* dependence of the interactions of PEI-coated silica particles in aqueous electrolyte at constant salt concentration, $c_0 \approx 0.12$mM NaCl
Fig. S17. Interactions of PDADMAC coated silica particles in electrolytes with different salt concentrations
Fig. S18. Matching of the experimental radial probability distribution functions $g(r)$s with those from BD simulations for experiments that vary the salt concentration c_0 for SiO_2 particles in water
Fig. S19. Matching the experimental radial probability distribution functions $g(r)$s with those from BD simulations for experiments varying the salt concentration for NH2-SiO_2 particles in water
Fig. S20. Matching the experimental radial probability distribution functions $g(r)$s with those from BD simulations for experiments varying the p*H* for SiO_2 particles in water
Fig. S21. Matching the experimental radial probability distribution functions $g(r)$s with those from BD simulations for experiments varying the p*H* for COOH particles in water
Fig. S22. Matching the experimental radial probability distribution functions $g(r)$s with those from BD simulations experiments varying the p*H* for NH2-SiO_2 particles in water
Fig. S23. Matching the experimental radial probability distribution functions $g(r)$s with those from BD simulations for the polyelectrolyte coating experiments
Fig. S24. Matching the experimental radial probability distribution functions $g(r)$s with those from BD simulations for experiments in alcohols
Fig. S25. Overview of all experimental snapshots and corresponding digitized images

S1 Supplementary experimental methods

S1.1 Preparation of particle suspensions for microscopy measurements

In our experiments we study the behaviour of three distinct types of microspheres with different surface chemistries. The 3 main classes of particle involve SiO2 particles (Bangs Laboratories), amine derivatized silica particles (NH_2-SiO_2, referred to as "NH_2" or "NH_3^+" in the main manuscript) (microParticles GmbH) with an estimated NH_2 group content of >30 µmol/g, and carboxylated melamine formaldehyde (COOH-MF, referred to as "COOH" in the manuscript) particles (microParticles GmbH) with a carboxyl group content of 400 µmol/g. The particle size distributions provided by the manufacturer are shown in Fig. S1.

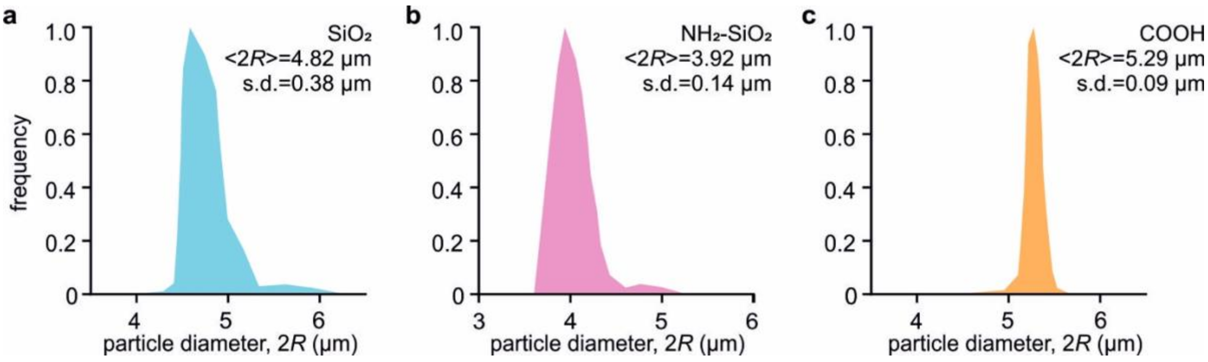

Fig. S1. Probability distributions of particle diameter for each particle type studied in experiments. (**a**) SiO_2, (**b**) NH_2-SiO_2 and (**c**) COOH particles. The distributions are taken from the manufacturer's measurements based on the Coulter principle. The mean and standard deviation (s.d.) of the particle diameter distributions are quoted in the inset. BD simulations of interparticle interactions (see Section S2.1) draw from these particle diameter distributions.

For experiments on SiO_2 and COOH particles in aqueous solution, particles were first centrifuged and resuspended in deionized water. Next, they were incubated in 5mM NaOH (99.99%, Alfa Aesar) solution for 10 minutes. Following this, they were centrifuged and resuspended in aqueous electrolyte of the required ionic strength around six times until the measured electrical conductivity of the supernatant solution converged to that of the pure electrolyte. Note that in general NaOH treatment is not essential and overnight exposure to DI water and rinsing in DI water are equally effective treatments prior to clustering experiments (see Fig. S11). However, NaOH pre-treatment was necessary in order to observe strong clustering in COOH particles. This is likely because a basic solution favours deprotonation of weakly acidic groups.

NH_2-SiO_2 particles were first washed in deionized water and then resuspended multiple times in aqueous electrolyte solution until the measured supernatant conductivity converged to that of the pure electrolyte. The suspension was further sonicated for cases in which a large population of 'sticking' particles were observed. The presence of stuck particles in the experimental data gives rise to small "dimer" peaks at interparticle separations $2R$ in the measured $g(r)$ which cannot be entirely eliminated (indicated as 'd.p.' in Fig. 2b, Fig. 3c, Fig. 5c).

In experiments on mixtures of SiO_2 and COOH particles (Fig. 4), the two types of particles were initially mixed at a 1:1 ratio, then incubated in 10 mM NaOH solution for 10 minutes. Thereafter the procedures were the same as described above.

For experiments on colloidal dispersions in alcohols, NH_2-SiO_2 particles were first centrifuged and resuspended in deionized water, followed by resuspension in ethanol (≥99.8%, Sigma-Aldrich) or isopropanol (≥99.5%, Sigma-Aldrich). The process of centrifugation and resuspension was repeated multiple times until the value of the supernatant conductivity converged to that measured for the pure alcohol. COOH particles were first centrifuged and resuspended in deionized water, followed by resuspension in ethanol, and final resuspension in either ethanol or isopropanol for measurements.

S1.2 Electrolyte solution preparation and measurements of solvent properties

For experiments examining the dependence of interparticle interactions on the ionic strength of the electrolyte (Fig. 1), various concentrations of sodium chloride (NaCl, 99.998%, Alfa Aesar) solution were prepared in deionised water, whose measured conductivity corresponds to a background concentration of monovalent ions of $c_0 \approx 5$ μM. The ionic strength of the various electrolyte solutions in our experiments was determined using measurements of electrical conductivity, s, performed using a conductivity meter (inoLab® Cond 7110). A calibration curve of standard solutions was used for this purpose (Fig. S2). For water, we may estimate the theoretically expected slope, a, in the equation $c_0 = as$ using the expression $a = 4\pi\eta a_{\mathrm{h}}/e^2 N_A$ where $\eta =$ 0.89 cP is the viscosity of water at a temperature of 298 K, a_{h} is the average hydrodynamic radius of the ions in solution, e the elementary charge and N_{A} the Avogadro number[1]. Using ionic radii 1.01 Å and 1.82 Å for Na^+ and Cl^- ions given in Ref. 2, this expression yields a value of a =102.4 kg mol A^{-2} s^{-3}, which is only about 30%

larger than the experimentally measured value for water of 77.5 kg mol A^{-2} s^{-3}as shown in Fig. S2a.

In order to convert the measured electrical conductivity to a background salt concentration in alcohols, we used the same calibration relationship as for aqueous electrolytes as shown in Fig. S2, but corrected the inferred concentrations for the viscosity of the alcohol as suggested in previous work[1] (viscosity values used for ethanol and isopropanol were 1.1 cP and 2.4 cP respectively). This procedure assumes that the hydrodynamic radii for the ionic species are identical in both water and alcohols, and it is possible therefore that our experimentally inferred salt concentration values in alcohols are not highly accurate.

In experiments exploring the pH dependence of interparticle interactions (Fig. 2), the pH of the electrolyte was adjusted to the desired value by adding either hydrochloric acid (HCl, 99.999%, Alfa Aesar) or a buffering agent tris(hydroxymethyl)aminomethane (TRIS, ≥99.9%, Carl Roth) to deionised water. Addition of acid or buffering agent to deionised water raised the conductivity of the solution to a value between 1 and 30 μS/cm (0.01 to 0.25 mM) depending on the target pH value. For experiments performed at variable pH, the ionic strength in the electrolyte was maintained constant (to within ±0.02 mM) across the entire range of pH in a given experimental series via the addition of a variable amount of NaCl. The pH value of the aqueous solution was taken as the mean value of three consecutive measurements using a pH meter (Horiba PH-33). The pH of pure alcohol samples was inferred by extrapolation of pH values measured for water-alcohol mixtures (Fig. S2b).

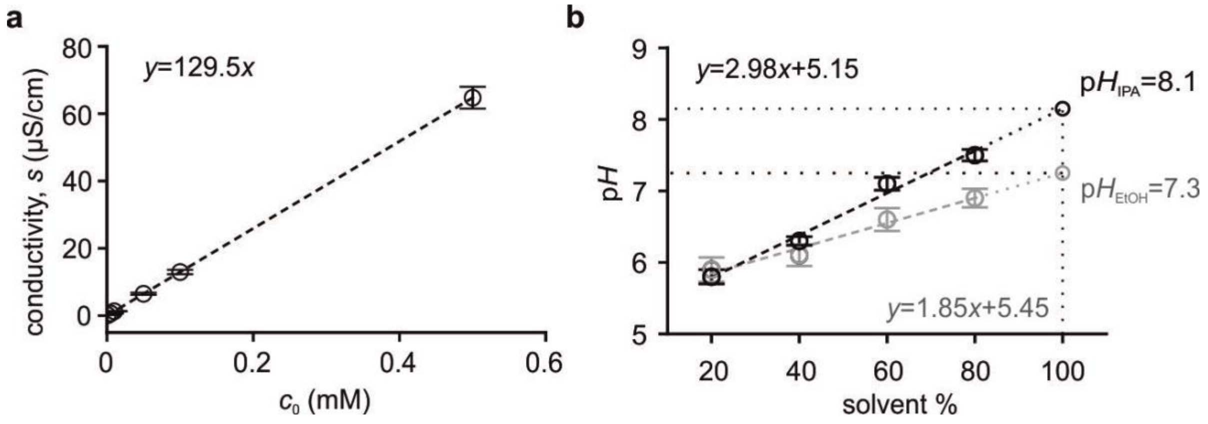

Fig. S2. Calibration curves underlying salt concentration and pH measurements. (**a**) Dependence of NaCl solution conductivities on salt concentrations of standard solutions. Data are presented as measured values ± 5% instrumental error. (**b**) Measurement of the pH of alcohol-water mixtures for ethanol (EtOH -grey) and isopropanol (IPA - black) mixtures. Data are presented as averaged values ± S.D. of three set of measurements.

S1.3 Layer-by-layer coating of silica particles with polypeptides and polyelectrolytes

In the experiments presented in Fig. 3, we used alternating coatings of positively and negatively charged polyelectrolytes on plain SiO_2 particles. Coatings were applied in the following pairs of combinations of positively and negatively charged polyelectrolytes: poly-L-lysine hydrobromide (poly-K, Molecular weight ≥300,000, Sigma) and poly-L-glutamic acid sodium salt (poly-E, mol. wt. 50,000-100,000, Sigma); poly(diallyldimethylammonium chloride) (PDADMAC, mol. wt. 200,000-350,000, Aldrich) and poly(sodium 4-styrenesulfonate) solution (PSS, mol. wt. ~70,000, Aldrich), and finally poly(ethyleneimine) solution (PEI, mol. wt. ~750,000, Sigma) and PSS.

In order to coat the particles, plain SiO_2 particles were first centrifuged and resuspended in deionised water, followed by incubation in 5 mM NaOH solution for 10 minutes, resuspension in deionised water, and repetition of the resuspension process until the supernatant conductivity converged to that of deionised water. The washed particles were then incubated in the polyelectrolyte solution at 0.1% w/v for 20 minutes with occasional vortexing to improve mixing. The coated particles were centrifuged and resuspended in deionised water to remove any excess polymer and the resuspension procedure repeated until the conductivity of the supernatant no longer changed. Subsequent layers of polymer coatings were applied by repeating the coating procedure described above with the corresponding oppositely charged polyelectrolyte. The sign of the surface charge of each coating layer was confirmed by zeta potential measurements (Zetasizer Nano Z, Malvern Panalytical).

S1.4 Cuvette preparation and sample loading

We used a glass cuvette with a polished flat-well of 1 mm depth (20/C/G/1, Starna Scientific), as shown in Fig. S3b for all video microscopy measurements. The cuvette was cleaned with piranha solution (3:1 mixture of concentrated sulfuric acid and 30 wt. % hydrogen peroxide solution) and then rinsed with deionised water thoroughly. A glass cuvette naturally provides a negatively charged surface in water, ethanol and isopropanol for experiments with negatively charged particles. For experiments with positively charged particles, the entire cuvette was coated with 1% w/v PEI solution, rinsed and dried under N_2 to provide a thin layer of positively charged polymer coating.

To load the cuvette, the prepared particle solution was carefully pipetted into the well and sealed with the cover slide such that the device was air-bubble-free and held together by capillary force.

S1.5 Microscopy

The optical microscope was constructed using a 470 nm collimated LED light (M470L4, ThorLabs), a 10x objective (Olympus UPlanSApo) and a CCD camera (DCU223M, ThorLabs) for recording images (Fig. S3). The sample holder was placed onto a carefully balanced pitch, roll and tilt platform (AMA027, ThorLabs). Follwing complete settling of particles in suspension to a plane near the bottom surface of the cuvette, which takes about 2 mins typically, the focus was adjusted such that a clear intensity maximum was observed for all particles. All measurements were performed after complete settling. The LED light intensity was adjusted such that the intensity maxima of illuminated particles did not exceed the saturation value of the camera, enabling accurate particle localization. In general, in the experiments where particles form clusters, we initially note the formation of small clusters characterised by strong interparticle interactions. With the passage of time (several tens of minutes), clusters could be found to grow in size, but the strength of the interparticle interactions may also alter as the pH and ionic strength of the solution can change. Since the interparticle interaction is expected to strongly depend on pH and salt concentration and we do not actively control these values in the observation chamber, time evolution of the structure of the suspension is not unexpected.

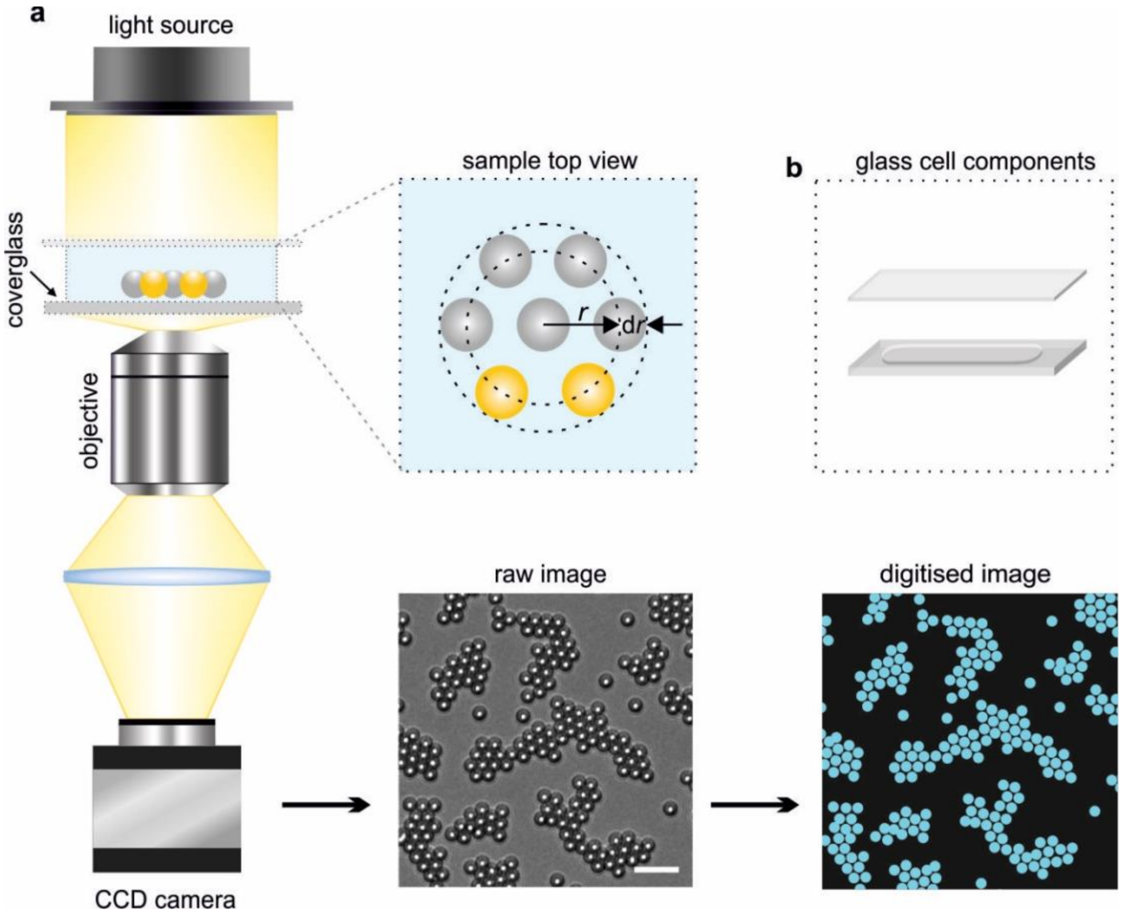

Fig. S3. Experimental set-up for measurements on 2-d colloidal dispersions. (**a**) Colloidal particles levitating above the coverglass surface are illuminated and imaged on to a CCD camera for a period of 30 seconds (scale bar: 20 μm. Particle coordinates are extracted using single particle tracking software (see Section S1.6). This information is used to generate probability density function, $g(r)$, profiles as well as digitised images. In the digitised microscopy images, particles are represented as coloured discs of uniform diameter $2R$ on a black background (see Section S1.6 and Fig. S25). (**b**) Diagram of the microscopy observation cell used in all experiments. The glass cell chamber has a depth of 1 mm and a nominal volume of 0.31 mL and the bottom coverglass surface is 1.25 mm thick.

S1.6 Video Recording and data processing

Sequential images of the two-dimensional suspension of colloidal particles were taken with ThorCam™ Software at a constant frame rate of 5, 10 or 30 frames per second (fps) for 150-500 frames using an exposure time of ≈0.5 ms The images were processed based on the radial symmetry method using the TrackNTrace particle tracking framework, where the particle centre maximum is detected[3,4]. The localization precision for a static SiO_2 particle during a 100-second measurement at an exposure time of ≈0.5 ms was found to be less than

20 nm, as shown in Fig. S4. In the analysis of experimental images, coordinates of all particle centres were extracted from the recorded frames and the radial distribution function curve $g(r)$ calculated and averaged over all images. This uncertainty in particle localization adds in quadrature with the uncertainty on the true mean diameter of the subset of the particles in any given experiment. We therefore estimate an overall uncertainty in measured separations in a given measurement at approximately ± 100 nm for silica particles.

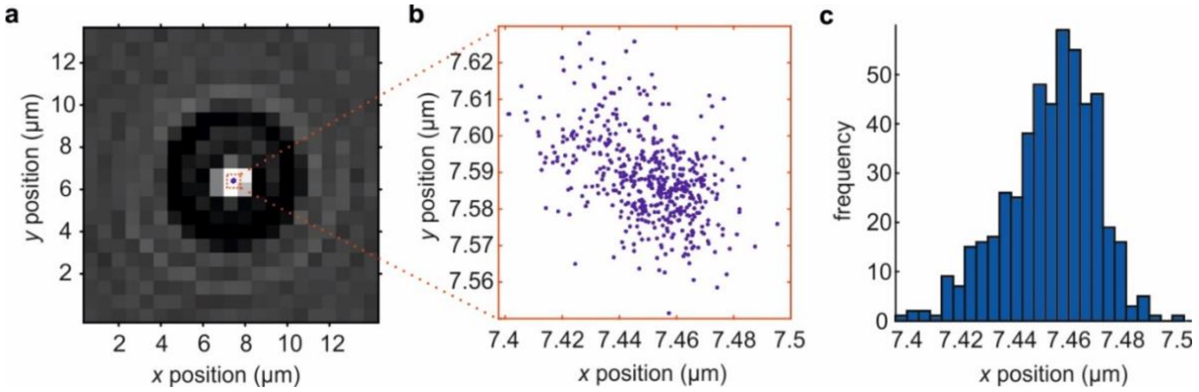

Fig. S4. Spatial localization accuracy of a single immobilized SiO_2 particle. (**a**) Optical microscopy snapshot of a single SiO_2 particle immobilized on the glass surface. (**b**) Scatter plot of particle location determined over 500 frames of imaging using TrackNTrace, with a standard deviation in the *x* and *y* dimensions of 16 nm and 12 nm, respectively. (**c**) Histogram of particle location in the *x* dimension.

In order to clearly distinguish between experiments on particles with different signs of particle charge in three different solvents, the recorded images were digitized and false-coloured (Fig. S3). The digitization process involved extracting the coordinates of all particle centres. Each particle centre was then presented as a disc of diameter corresponding to the nominal particle diameter $2R$. Note that the particle samples have a size distribution (Fig. S1) which is not taken into account in the digitization process since the individual particle sizes are not known with high accuracy. This can occasionally result in overlapping of particles in the digitized images and videos which are provided for illustrative purposes only. Sporadic failure to detect particles in individual raw images can also cause the appearance of "blinking" particles in the digitized videos, which is not present in the raw data (Supplementary Video 1-7). This was found to occur in particular for COOH particles in water due to the lower refractive index of melamine resin compared to silica. The average particle detection efficiency over all experiments was greater than 98%.

S2 Supplementary simulation methods

S2.1 Brownian Dynamics (BD) simulations of interparticle interactions

In Figs. 1, 2, 3 and 5 we have presented forms of the underlying pair interaction potentials $U(x)$ that have been inferred from BD simulations to match the experimentally measured radial probability distribution functions, $g(r)$s. This section provides a description of procedures and further detail on BD simulations. From Table S2 to Table S12 we provide a comprehensive table of the $U(x)$ parameters used to match simulations to the experimental data. We also present graphs that overlay the experimental and simulated $g(r)$s for experiments shown in Fig. S18 - Fig. S24.

We performed BD simulations of a two-dimensional distribution of interacting spheres using the BROWNIAN package in the Large-scale Atomic/Molecular Massively Parallel Simulator (LAMMPS) software[5]. Example input and necessary simulation files are provided in our Figshare repository, available at: https://doi.org/10.6084/m9.figshare.c.6132003.

The relevant timescales for colloidal motion in our BD simulations include a diffusive timescale $\tau_{\mathrm{diff}} \approx \langle \mathrm{MSD} \rangle / 2D$ which is on the order of 1s. In this expression, $\langle \mathrm{MSD} \rangle \approx (0.30 \times 10^{-6}\mathrm{m})^2 \approx 1 \times 10^{-13}\mathrm{m}^2$ denotes a typical mean squared displacement in x for a random walker exploring the spatial range of a representative pair interaction potential $U(x)$ in this study, and D ($\approx 1 \times 10^{-13}\mathrm{m}^2\mathrm{s}^{-1}$) is the diffusion coefficient in water of a particle of diameter $2R = 4.82$ µm. Expanding the displacement $\Delta x = x - x_{\mathrm{min}}$ to the lowest order around the potential minimum, x_{min}, we have $U(\Delta x) \approx k\Delta x^2$, which permits us to deduce a viscous relaxation time $\tau_{\mathrm{r}} = \frac{\gamma}{k} \approx 0.01\mathrm{s} - 0.1\mathrm{s}$. Here, $\gamma = 6\pi\eta R$ denotes the Stokes drag on the particle, where η is the viscosity of the solvent and k denotes a stiffness parameter characterizing the pair interaction potential well. We further have the momentum relaxation time $\tau_{\mathrm{p}} = \frac{m}{\gamma} = 2.5$ µs for 4.82 µm diameter silica particles which shows that $\tau_{\mathrm{p}} \ll \tau_{\mathrm{r}}$, placing the problem in the overdamped Langevin or Brownian Dynamics regime.

The overdamped Langevin equation of motion for each colloid can be written as:

$$-\nabla U(x) - \gamma\dot{\vec{x}} + \vec{f}(t) = 0 \qquad \text{S 1}$$

In this equation, $\vec{f}(t)$ is the random thermal force acting on the particle which has a Gaussian probability distribution with zero mean, $\langle \vec{f}(t) \rangle = 0$, and correlation function $\langle \vec{f}_i(t)\vec{f}_j(t') \rangle = 2\gamma k_{\mathrm{B}} T \delta_{i,j}\delta(t-t')$. The above equation can be integrated in time to obtain

particle displacements and trajectories as a function of time. The simulation timestep ($\approx 2.5 \times 10^{-5}$s) in the integration was chosen to be much smaller than τ_{r}.

In Eq.S 1, $U(x)$ is the total pairwise interaction potential between the colloids, which we assume to be of the form:

$$U(x) = Ae^{-\kappa_1 x} + Be^{-\kappa_2 x} + U_{\mathrm{vdW}} \qquad \text{S 2}$$

Here the first term represents the overall repulsive electrostatic free energy of interaction, $\Delta F_{\mathrm{el}}(x) = A\exp(-\kappa_1 x)$, with $A > 0$, and the second term, $\Delta F_{\mathrm{int}}(x) = B\exp(-\kappa_2 x)$, denotes the free energy contribution arising from interfacial solvation[2]. Note that $\kappa_2 < \kappa_1 \approx \kappa$. Importantly the $\Delta F_{\mathrm{int}}(x)$ term implies an attractive contribution to the total free energy for negatively charged particles[6,7]. The third term represents the van der Waals (vdW) attraction between silica particles in solution, for which we have used the expression derived in Ref. 8:

$$U_{\mathrm{vdW}} = -\frac{A_{\mathrm{H}}}{12}\left\{\frac{y}{x'^2 + x'y + x'} + \frac{y}{x'^2 + x'y + x' + y} + 2\ln\frac{x'^2 + x'y + x'}{x'^2 + x'y + x' + y}\right\} \qquad \text{S 3}$$

In the above equation, $x' = \frac{x}{2R_1}$ and $y = \frac{R_2}{R_1}$, where R_1 and R_2 are the radii of the corresponding particles. BD simulations were performed on taking into account the experimentally determined polydispersity in particle sizes as shown in Fig. S1. This implies that at simulation level, variable particle radius is taken into account to the lowest level of approximation (i.e., the interaction potential remains fixed and independent of the size of the particles which would not be true in practice). Using a value of the Hamaker constant A_{H} =2.4 zJ (taken from Ref. 9 and which is in agreement with other literature estimates[10]), we found that U_{vdW} made a small contribution ($< -0.5\, k_{\mathrm{B}}T$) to the total interaction energy at large separations, $x \geq 0.2$ µm, i.e., for the majority of experiments in this work. We emphasize therefore that the vdW interaction cannot be responsible for the strong and long-ranged minima implied by the clusters observed in experiment. However, for experiments at higher salt concentrations ($c_0 \approx$ 1mM, see Fig. 1d), the vdW interaction can make a more significant contribution ($\approx -1\, k_{\mathrm{B}}T$) to the interaction at separations $x \approx 0.1$ µm, and for this reason it was included in our expression for $U(x)$ in modelling these measurements.

The experimentally measured $g(r)$ curve provides an estimate of the location of the minimum in the pair potential x_{min}. In Eq. S 2, the screening length in solution $\kappa_1^{-1} = \kappa^{-1}$(the Debye length), is known from the measured salt concentration. We then use a trial value of the interaction free energy at the minimum, $U(x_{\mathrm{min}}) = w < 0$, to obtain initial values for the parameters A and B as inputs for the pair interaction potential $U(x)$, using the equations:

$$A = -\frac{w\kappa_2\ \exp(\kappa_1 x_{\min})}{\kappa_1\ - \kappa_2}\ ; B = \frac{w\kappa_1\ \exp(\kappa_2 x_{\min})}{\kappa_1\ - \kappa_2} \quad \text{S 4}$$

where we have taken $\kappa_2/\kappa_1 \approx 0.95$, as suggested in Ref. 7. We note however that this ratio is not a strict requirement and that we may also treat it as a free parameter which yields an alternate set of parameters A, B , κ_1 and κ_2 that can provide equally good qualitative agreement with the experimental data (see e.g. Table S5).

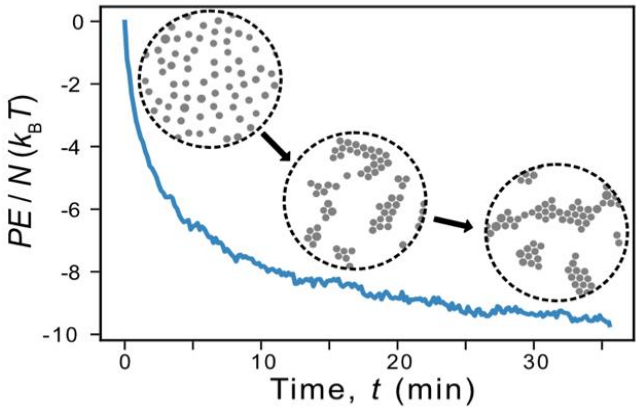

Fig. S5. Convergence of the potential energy per particle, *PE*/*N*, over the course of a BD simulation. The simulation box measures $200 \times 200\ \mu m^2$ and contains a number of particles, $N \approx 300$, which corresponds to a particle density typical for experiments. The initial configuration of colloids at $t = 0$ involves large average interparticle distances ($x > 1$ µm) so that $PE/N =\ 0$. In the above case the system is permitted to evolve under the influence of an attractive pair potential $U(x)$ (A =32580, $B = -25848$, κ_1 =13.16, κ_2 =12.5), which has a minimum of $w = -6k_B T$ at $x_{\min} = 0.43$ µm. The system exhibits the formation of strong, stable and slowly reorganizing clusters within a period of a few minutes, with clusters growing in size over time. In this case, the asymptotic value of $PE/N \approx -10\ k_B T$, which corresponds to an average number of nearest neighbours of ≈ 3.5, as is evident from the cluster configuration at large *t*. Evolution of the initial configuration of particles (left inset) over time shows the formation of particle clusters (right inset). Final, converged particle configurations are used to generate $g(r)$ distributions for comparison with experiments.

The BD simulation set-up was validated by comparing the Stokes-Einstein value of the particle diffusion coefficient, $D = k_B T/6\pi\eta R$, with that inferred from a mean squared displacement (MSD) analysis for simulated motion of a single colloid diffusing in 2 dimensions. We also ensured that the pairwise interaction potential $U(x)$ input into a simulation of two interacting particles could be recovered via Boltzmann inversion of the probability distribution of interparticle distances using the procedure described in Section S4.2.

Particle configurations for the BD simulations were initialized via random particle placement in a $200 \times 200\ \mu m^2$ simulation box that reproduced the experimental particle density (≈ 0.008 particles/μm^2). The polydispersity of the simulated colloids was drawn from the manufacturer's size distribution for each particle type, as shown in Fig. S1. Periodic boundaries were applied in the x, y dimensions whilst the z dimension was held finite. The z coordinate of the colloids were fixed at a constant height throughout the simulation, ensuring a 2D system, mimicking experiment. Convergence of the potential energy (PE) per particle in our BD simulations was monitored over time (Fig. S5). Particle positions used for the calculation of the final simulated $g(r)$s were collected once the value of the potential energy reached a stationary value, after approximately 30 minutes of simulation time in a simulation involving a strongly attractive $U(x)$ of a well depth of several $k_B T$ (Fig. S5).

Agreement between the simulated and the experimental $g(r)$s was assessed for a trial input pair interaction potential $U(x)$ and the value of the well depth w adjusted in subsequent BD simulations if required, in order to attain a final simulated best match to the experimental data. This procedure permitted us to infer the functional form of an underlying pair interaction potential $U(x)$. These BD simulations assume the interactions between the particles may be regarded as pairwise additive. As discussed in the main text and Section S3.4, a pair potential inferred from BD is not expected to accurately reflect the true pair potential of the system particularly in the light of factors such as particle polydispersity which exert substantial influence on the extracted $g(r)$ profile (Fig. S6). In fact given the enormous variability in particle properties, a true pair potential may in fact constitute an idealisation that cannot be probed in practice.

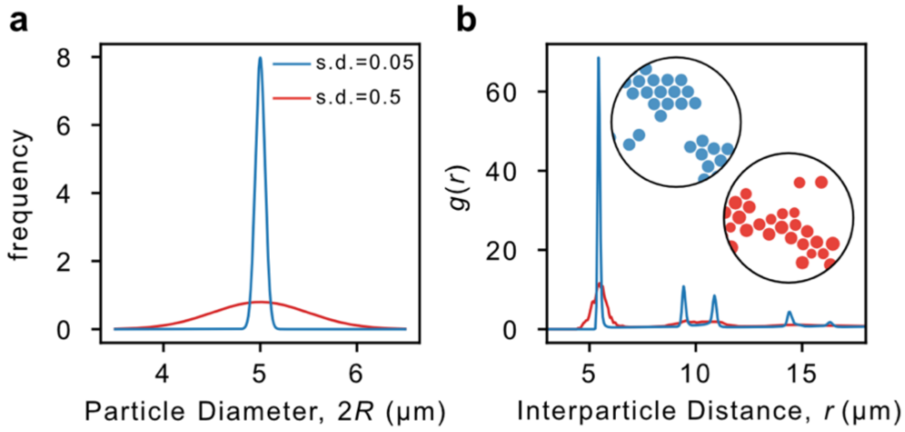

Fig. S6. Influence of the particle polydispersity on the *g*(*r*) inferred from a BD simulation. (**a**) Two normally distributed particle size distributions with mean 5 μm and standard deviations of $\mathrm{s.d.} = 0.05$ and 0.5 (blue and red curves respectively). Particle radii drawn from these size distributions were fed into independent BD simulations and allowed to evolve under the influence the same attractive pair potential $U(x)$ (A =27150, B = −21540, κ_1 =13.2, κ_2 =12.5), which has a minimum of $w = -5k_{\mathrm{B}}T$ at $x_{\mathrm{min}} = 0.43$ μm. (**b**) $g(r)$s determined from BD simulations of the two particle size distributions in (a), clearly showing a high sensitivity of the $g(r)$ to the particle polydispersity. The $g(r)$ corresponding to the narrow size distribution ($\sigma = 0.05$, shown in blue) is characterised by tall, sharp peaks. The majority of clusters formed are arranged in a perfect hexagonal pattern (blue particles, shown inset). The $g(r)$ corresponding to the larger size distribution ($\sigma = 0.5$, shown in red) on the other hand, is characterized by broad, noisy peaks of much smaller height. In simulations of particles with a broader size distribution the clusters display less crystalline order (red particles, shown inset). Our experimental data also clearly demonstrates the role of particle polydispersity, with COOH particles (which have a narrow particle size distribution, see Fig. S1) displaying tall, sharp peaks in the experimental $g(r)$s (see Fig. S21), in contrast to SiO_2 particles which have a broader size distribution and broader $g(r)$ peaks (see Fig. S20).

S2.2 Molecular Dynamics (MD) simulations of alcohols at interfaces

The excess electrical potential due to the orientation of solvent molecules at an interface, φ_{int} is required as an input to interfacial solvation model in order to calculate theoretical $U(x)$ curves (see Section S3.2). In order to estimate $\varphi_{\mathrm{int}}(\sigma)$ as a function of surface electrical charge, σ, we performed molecular dynamics (MD) simulations with the GROMACS MD code[11]. We examined the behaviour of a solvent phase in contact with a model surface composed of oxygen atoms in a parallel plate capacitor setup, as described extensively in previous work[6,12]. Example input files, force field parameters and code for the analysis of the simulations performed in this study are available in our Figshare repository: https://doi.org/10.6084/m9.figshare.c.6132003.

Prior to running MD simulations in the capacitor setup, we first ran preliminary simulations of a box of 7500 isopropanol molecules, without the capacitor wall atoms, under constant pressure, maintained with the Parrinello-Rahman pressure coupling method. The length of the box in z was allowed to fluctuate, whilst keeping the x, y dimensions fixed to those of the capacitor walls of fixed area. This equilibrated slab of solvent was then sandwiched between capacitor plates comprised of positionally restrained oxygen atoms that only support Lennard-Jones interactions (see Fig. S7). In our simulations, isopropanol and ethanol molecules were both parametrized with the CHARMM36 forcefield[13]. As in previous work, the plates are $\approx 10 \times 10$ nm^2 in area and are separated by approximately 8 nm of solvent medium in the z-direction[6,12]. This ensures that any oscillations in the solvent density or dipole

moment profiles attain bulk-like properties at a location z_{mid} in the middle of the capacitor. A subset of the atoms belonging to the first layer in each wall (in direct contact with the solvent) were randomly assigned a positive (left plate) or a negative charge (right plate) in order to generate an electric field of specific strength in the box whilst maintaining electroneutrality within the box. The capacitor system simultaneously yields estimates of $\varphi_{\mathrm{int}}(\sigma)$ for both positive and negative values of σ and provides a well-defined system for comparing solvation at a macroscopic surface with a continuum electrostatics model.

Next, a second round of equilibration was carried out for the entire capacitor system, including the capacitor walls, and consisted of a short NVT run with a v-rescale thermostat, followed by 500 ps in an NPT ensemble where only the z-dimension of the box was allowed to fluctuate, keeping the the x, y dimensions fixed. This ensured that solvent molecules were maintained at the correct density throughout the simulation box. Following this procedure, production MD runs of 20ns duration were performed in an NVT ensemble, with trajectory frames written every 20 ps. The particle mesh Ewald (PME) method was used to evaluate the long-range electrostatic interactions using a 1 Å grid spacing and a short-range cut-off of 12 Å. The Lennard-Jones interactions were smoothed over the range of $10 - 12$ Å using the force-based switching function. We scaled the z-dimension of the box by a factor 2 for Ewald summation only and applied the 3dc correction of Yeh and Berkowitz to remove artificial polarization induced by neighbouring image dipoles[14].

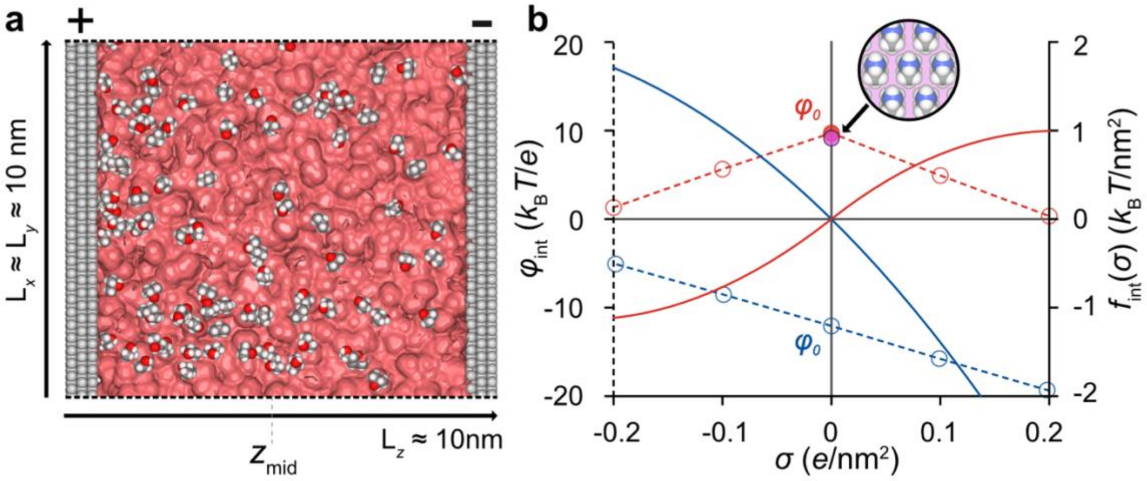

Fig. S7. MD simulations of isopropanol in a capacitor setup: calculation of $\varphi_{\mathrm{int}}(\sigma)$ for an alcohol at an interface (**a**) Schematic representation of a simulation cell corresponding to the "capacitor system" used to calculate the excess interfacial potential $\varphi_{\mathrm{int}}(\sigma)$ that arises due to symmetry broken orientational behaviour of

solvent molecules at an interface. Solvent molecules are confined between two plates of dimensions $\approx 10 \times 10$ nm^2, each made up of three layers of hexagonally packed, positionally restrained oxygen atoms, separated by ≈ 8 nm along the z-direction. A subset of the interfacial wall atoms on the left and right plates are randomly assigned integer charge of +1e (left plate) and -1e (right plate) in order to generate an overall charge density of $\pm\sigma$ on the respective plates. (**b**) The excess interfacial potential $\varphi_{\mathrm{int}}(\sigma)$ (left axis, dashed lines and circle markers) and the corresponding excess free energy of interfacial solvation $f_{\mathrm{int}}(\sigma)$ (right axis, solid curves) for isopropanol and water for comparison (shown in red and blue respectively). The reorientation of IPA molecules at an O-atom surface that increases in charge density causes φ_{int} to decrease in magnitude, go through zero, and turn increasingly negative. Note that surface chemistry can have a substantial impact on the behaviour of the solvation potential (φ_{int}) in organic solvents[12]. That said, the value of φ_0 for isopropanol molecules in contact with a surface patterned with amine groups (circle inset) is also shown (pink data point), confirming that the sign and magnitude of φ_0 for a more realistic model surface remain unchanged from the O-atom-surface value.

S3 Supplementary calculation methods

S3.1 A brief overview of the interfacial solvation model of interparticle interactions

Our present understanding of the physical mechanism underpinning the proposed interaction is founded in the fact that a solvent molecule at an interface, e.g., that between a charged particle and the fluid, displays orientational behaviour that is different from its counterpart in the bulk. Like dipoles in the bulk fluid, the net orientation of interfacial solvent molecules depends on the local electrical field, but unlike molecules in the bulk, the average orientation of interfacial molecules has additional character due to the broken symmetry created by an interface. This orientational behaviour induced by the interface is associated with an 'excess' contribution to the free energy that is not included in continuum descriptions of electrostatic interactions on which the DLVO theory is based. The assumption that the solvent may be treated as a dielectric continuum does not admit a molecular-level description of the medium and cannot therefore predict any free energy contribution arising from orientational behaviour of interfacial solvent molecules. Furthermore, simulation studies that seek to account for finite ion size and other experimental aspects in greater detail, and yet work with a 'primitive model' of the solvent, which treats water as a smooth continuum, would not be expected to capture our experimentally observed long-ranged attraction between like-charged particles. Our working model of the interfacially induced interaction - described in Refs. 6,7- thus goes beyond PB theory to explain the experimental observations and will be outlined in this section.

When two like-charged particles approach each other in a fluid their net charge decreases via a process called 'charge regulation', minimising the free energy of the system[15]. Nonetheless, the interparticle interaction remains net repulsive. The reduction in particle charge however entails an 'excess' orientational free energy contribution from interfacial solvent molecules. Our current working model of the overall interaction assumes that this excess, interfacial contribution to the free energy superimposes on the traditional electrostatic free energy. The result is a pair interaction potential that can depart strongly from the continuum expectation, turning attractive or repulsive at long range depending on the experimental conditions.

Notably, although our proposed interfacial free energy arises from solvent molecules located within about 0.5-1 nm from an interface, it can in fact make substantial contributions to the total free energy at large interparticle separations. This is because charge regulation – the underlying phenomenon which triggers the free energy contribution – is long ranged. We emphasize that at present our proposed mechanism does not invoke long-range correlations in or

ordering of solvent molecules in the bulk electrolyte[16]. Furthermore, at small distances from the interface, both interfacial spectroscopy measurements and molecular simulations on neat water are in unequivocal agreement concerning surface charge dependent molecular orientation[17-20]. We point out that the interaction mechanism we propose shares some features with, but is substantially different from the well-established *short-range* "hydration force" which manifests at very small separations, typically < 3 nm, between surfaces[21].

We now summarise the main ingredients of an effective model of interparticle interactions in solution. We write the total interparticle interaction potential between two particles in solution as the sum of the van der Waals (vdW) contribution, U_{vdW}, the mean field electrostatic contribution, ΔF_{el}, and an interfacial free energy contribution, ΔF_{int}, as follows

$$U^{*}_{\mathrm{tot}}(x) = U_{\mathrm{vdW}}(x) + \Delta F_{\mathrm{el}}(x) + \Delta F_{\mathrm{int}}(x) = U_{\mathrm{DLVO}}(x) + \Delta F_{\mathrm{int}}(x) \qquad \text{S 5}$$

In Eq. S 5, $U_{\mathrm{DLVO}} = U_{\mathrm{vdW}} + \Delta F_{\mathrm{el}}$ represents the total interparticle interaction potential as postulated in the DLVO theory.

For large spheres at large distances from each other (say $\kappa x > 2$), $U_{\mathrm{vdW}}(x)$ may be ignored and the interaction potential may be written as

$$U_{\mathrm{tot}}(x) = \Delta F_{\mathrm{el}}(x) + \Delta F_{\mathrm{int}}(x) \approx A\exp(-\kappa_1 x) + B\exp(-\kappa_2 x) \qquad \text{S 6}$$

Here A and B can be treated as constants, $\kappa^{-1} \approx \frac{0.304}{\sqrt{c_0}}$ nm is the Debye length in an aqueous electrolyte containing monovalent salt at a concentration of c_0 moles/litre, and represents the length scale on which electrostatic interactions are screened by counterions in solution[7]. Furthermore, $\kappa_1 \approx \kappa$ is the effective inverse decay length of the electrostatic free energy (ΔF_{el}) term, and we have $\kappa_1 > \kappa_2 \approx 0.95\kappa_1$[7]. Note that the condition $\kappa_1 > \kappa_2$ plays a key role in the appearance of a minimum and, within our model, arises from examining the spatial dependence of the calculated interfacial free energy term, $\Delta F_{\mathrm{int}}(x)$. However, further details of the microscopic physical origin of this additional effective screening length κ_2^{-1} and the relationship $\kappa_1 > \kappa_2$ may be further explored in future work. The interfacial solvation model[7] further suggests that

$$B \propto e z \varphi_{\mathrm{int}} \Gamma R^2 \frac{\mathrm{d}\alpha}{\mathrm{d}\psi_{\mathrm{s},\infty}} \psi_{\mathrm{s},\infty} \qquad \text{S 7}$$

where

$$\alpha = \frac{1}{1 + 10^{z(\mathrm{p}H - \mathrm{p}K)}\exp(z\psi_{\mathrm{s},\infty})} \qquad \text{S 8}$$

is the ionisation probability of a surface ionisable group of valence $z = \pm 1$ in the ionized state[15], which in turn implies

$$z\frac{\mathrm{d}\alpha}{\mathrm{d}\psi_{\mathrm{s},\infty}} = \frac{-10^{z(\mathrm{p}H-\mathrm{p}K)}\exp\left(z\psi_{\mathrm{s},\infty}\right)}{\left[1+10^{z(\mathrm{p}H-\mathrm{p}K)}\exp\left(z\psi_{\mathrm{s},\infty}\right)\right]^2} \leq 0 \qquad \text{S 9}$$

Eqs. S 8-S 9 above capture the phenomenon of charge regulation. Here, Γ denotes the number density of ionisable groups in the particle, while pH and pK denote the negative decadic logarithm of the proton concentration in solution and the equilibrium constant of proton dissociation of the groups respectively. In turn, $\psi_{\mathrm{s},\infty} = \frac{e\phi_{\mathrm{s},\infty}}{k_{\mathrm{B}}T}$ is the dimensionless electrical potential $\phi_{\mathrm{s},\infty}$ created at the surface of an isolated charged object by the ion distribution in the electrolyte, and e is the elementary charge.

In contrast to ϕ which is the electrical potential due to the ions in the system, φ_{int} represents an excess electrical potential created at a planar interface by dipoles representing solvent molecules in the vicinity[22]. Crucially, even at uncharged surface, this interfacial electrical potential, φ_0, is generally not zero and can take a positive or a negative sign depending on the solvent and the chemistry of the surface[6,12]. In general we may take $\varphi_{\mathrm{int}} \approx \varphi_0$, and as Eq.S 7 shows, the sign of φ_{int} can dramatically modify the form of the interparticle interaction potential, turning it attractive or repulsive at long range. Molecular simulation studies provide estimates of $\varphi_0 \approx -0.5$ to -0.1 V < 0 for an interface immersed in water with respect to the bulk phase[6,12,19,22-24]. Simulations further suggest $\varphi_0 \approx +0.2$ V > 0 for surfaces immersed in alcohols such as ethanol (EtOH) and isopropanol (IPA). Note that the quantity φ_0, the solvent-induced electrical potential at a neutral interface in our work, is similar to "the potential of zero charge" in electrochemistry and interface science, as well as the air-liquid interfacial potential[25,26].

The main experimental trends suggested by Eqs.S 5-S 9 are: (1) the breaking of charge reversal symmetry in interparticle interactions (e.g., negatively charged particles are characterised by $B < 0$ in water and may therefore attract, whereas positive particles imply $B > 0$, and should therefore repel), (2) use of a solvent in which the sign of φ_0 is opposite to that of water may reverse this trend (i.e., positive particles may attract whereas negatives repel), and (3) the magnitude of the solvation contribution to the interparticle interaction should depend strongly on the pH in solution. Importantly, when $\frac{\mathrm{d}\alpha}{\mathrm{d}\psi_{\mathrm{s},\infty}} \to 0$, which occurs outside the range of pH given approximately by $\mathrm{p}K + 0.2 \lesssim \mathrm{p}H \lesssim \mathrm{p}K + 2.5$, the electrostatic contribution to the free energy, $\Delta F_{\mathrm{el}}(x)$, should be the only surviving term in Eq.S 6. Here we

expect to obtain $U_{\text{tot}}^{*} \cong U_{\text{DLVO}}$ and the total interaction potential should revert to a form that agrees with the DLVO prediction.

It is worth noting here that although our theoretical results for interfacial solvation properties utilise molecular dynamics (MD) simulation data for model oxygen-atom surfaces, recent MD simulation studies on water orientation at a variety of more realistic surfaces whose chemistry ranges from that of inorganic silica to polymeric surfaces clearly reveal that essential features of interfacial water orientation, and the sign of the associated interfacial potential, φ_0, are preserved across these materials[12]. It is possible that the dominant role played by hydrogen bonding in water is responsible for the comparative indifference of qualitative aspects of the orientational behaviour of water at an interface to specific surface chemistry[12,22,23]. Interfaces with organic solvents may however be expected to show significant dependence of molecular orientation – and hence a dependence of both the sign and magnitude of φ_0 – on surface chemistry[12].

S3.2 Summary of the approach to calculating the total interparticle interaction

Figs. 1, 2 and 5 display pair interaction potentials, $U_{\text{tot}}(x)$, calculated using our interfacial solvation model as described previously[6,7]. The essential details of the model are recapitulated below. We solve the PB equation

$$\nabla^2\psi(\boldsymbol{r}) = \kappa^2 \sinh\psi(\boldsymbol{r}) \qquad \text{S 10}$$

for two hollow, identical spheres of radius R and surface charge density, σ, at a variable intersurface separation, x, immersed in an electrolyte with a concentration of monovalent salt c_0. Here ψ is the dimensionless electrical potential in the electrolyte and $\kappa = \sqrt{\frac{2e^2c_0}{\varepsilon\varepsilon_0 k_\text{B}T}}$ is the inverse of the Debye length - a measure of the distance over which the electrical potential decays from its surface value ψ_s, due to screening by the cloud of oppositely charged counterions in solution. Furthermore, e is the elementary charge, k_B is Boltzmann's constant, and ε is the relative permittivity of the electrolyte medium ($\varepsilon = 78.5$ for water at temperature $T = 298$ K). We calculate the potential distribution everywhere in the system subject to the boundary condition applied to the surface of each particle:

$$\sigma(R) = z\alpha(R)\Gamma e \qquad \text{S 11}$$

where $z = \pm 1$ denotes the sign of charge of the ionized group (e.g., $z = -1$ for an acidic group) and Γ is the number density of ionisable groups on the surface of the particle. In this

equation, α is the ionisation probability of a group on the surface of the particle and is given by Eq. S 8 [15,27].

Having obtained the distribution of electrical potential ψ in the entire system, we then evaluate the following free energy functional for the electrostatic free energy as described previously[27-30]. Thus we have:

$$F_{\mathrm{el}}(x) = -\int_V \left\{\frac{\varepsilon\varepsilon_0}{2} E(\boldsymbol{r};x).E(\boldsymbol{r};x) + 2c_0 k_{\mathrm{B}} T(\cosh\psi(\boldsymbol{r};x) - 1)\right\} \mathrm{d}V + \cdots \quad \text{S 12}$$

$$\Gamma k_{\mathrm{B}} T \int_S \ln\frac{1-\alpha(R;\ x)}{1-\alpha(R;\ \infty)} \mathrm{d}A$$

where $E(r;x)$ denotes the electric field at a point r in the electrolyte for an interparticle surface separation, x. Subtracting the value of the integral at very large separation say, $x = 20\kappa^{-1}$, gives the electrostatic interaction free energy, i.e., $\Delta F_{\mathrm{el}}(x) = F_{\mathrm{el}}(x) - F_{\mathrm{el}}(x = 20\kappa^{-1})$. We have also previously shown that the electrostatic interaction energy which takes the general screened Coulombic form given by $\Delta F_{\mathrm{el}} \propto \frac{\exp(-\kappa r)}{r}$ can be well approximated by a simple exponential for the large spheres and the comparatively small separations range in our experiments, i.e., we work with $\Delta F_{\mathrm{el}}(x) = A\exp(-\kappa x)$ since $x < R \gg \kappa^{-1}$, where $A > 0$ for like-charged particles[7].

Our model then introduces an interfacial free energy term, $F_{\mathrm{int}}(x)$, which represents a free energy contribution from the orientational behaviour of the interfacial solvent molecules. This term is calculated using MD simulation results for the excess electrical potential at interface $\varphi_{\mathrm{int}}(\sigma)$ as function of σ. Integration of the $\varphi_{\mathrm{int}}(\sigma)$ vs. σ relationship for a given surface type and solvent gives the solvation free energy $f_{\mathrm{int}}(\sigma) = \int_0^\sigma \varphi_{\mathrm{int}}(\sigma)\mathrm{d}\sigma$ per unit area of interface (Fig. S8).

Owing to charge regulation, the charge density σ at any point of the particle surface is a function of the inter-surface separation, x. As a result, $f(\sigma)$ becomes a function of interparticle separation. Thus, for a given value of x, the total interfacial free energy, F_{int}, can be calculated via the surface integral

$$F_{\mathrm{int}}(x) = \int_S f_{\mathrm{int}}(\sigma(R,x))\,\mathrm{d}A \quad \text{S 13}$$

Similar to the evaluation of ΔF_{el} we determine ΔF_{int} by evaluating the integral for each location x and subtracting the value at large separation $x = 20\kappa^{-1}$, i.e., $\Delta F_{\mathrm{int}}(x) = F_{\mathrm{int}}(x) - F_{\mathrm{int}}(x = 20\kappa^{-1})$. We further find that for the interaction of large particles in solution ($R >$

κ^{-1}), the interfacial solvation free energy at long range can also be well approximated by an expression of the form $\Delta F_{\rm int}(x) = B\exp(-\kappa_2 x)$, where $\kappa_2 < \kappa$ (Ref. 7) .

In contrast to $\Delta F_{\rm el}(x)$, which is generally always positive in the interaction of like-charged objects, the sign of B is governed by both that of the sign of the charge of the particles and that of the solvation potential, $\varphi_{\rm int}$, as displayed by Eq. S 7 (Ref. 7). We obtain the total interaction potential between a pair of particles by adding the two contributions to the free energy. Thus we have:

$$U_{\rm tot}(x) = \Delta F_{\rm el}(x) + \Delta F_{\rm int}(x) \qquad \text{S 14}$$

The assumption of free-energy additivity we invoke in writing Eq.S 14 has a long history. Dating back at least to the Derjaguin-Landau-Verwey-Overbeek (DLVO) theory, such assumptions are widely used in colloid science[31,32]. The assumption of superposition of hydration and electrostatic forces, for instance, has been explicitly tested in atomistic simulations and found to hold within accuracy limits under the relevant conditions[33].

Importantly, a value of $B < 0$ in Eq.S 6 implies an attractive contribution to the pair potential from the interfacial term, $\Delta F_{\rm int}$, which is opposite in sign to and therefore counteracts the interparticle electrostatic repulsion, $\Delta F_{\rm el}$. Conversely, $B > 0$ implies that $\Delta F_{\rm int}$ is a positive quantity that increases monotonically with decreasing separation, reinforcing $\Delta F_{\rm el}$. Since $A > 0$ in the interaction of like charged objects, Eq.S 6 thus implies the possible occurrence of a long ranged minimum in $U_{\rm tot}(x)$ when $B < 0$, provided that $A/|B| > 1$. If on the other hand $A/|B| \gg 1$ or $\ll 1$, we expect monotonically repulsive or attractive interaction potentials at long range, respectively.

Furthermore, under conditions where $z\frac{{\rm d}\alpha}{{\rm d}\psi_{\rm s}} < 0$, Eq.S 7 demonstrates that the overall sign of B is determined by the signs of $\varphi_{\rm int}$ and $\psi_{\rm s,\infty}$. Thus for $\frac{{\rm d}\alpha}{{\rm d}\psi_{\rm s}} \neq 0$, our model suggests that $\Delta F_{\rm int} < 0$ for weakly negatively charged particles in water, since both φ_0 and $\psi_{\rm s,\infty} < 0$. Whereas for positively charged particles where $\psi_{\rm s,\infty} > 0$, we have $\Delta F_{\rm int} > 0$. Therefore, for a particle with a given sign of charge, the sign of B, which governs its interactions with a similar neighbour, may be said to be determined by that of φ_0. In practice, surfaces in solution rarely exceed a charge density magnitude of 0.3 e/nm^2 (Fig. S8a, bottom panel). Consequently, for objects in water we generally expect $\varphi_{\rm int} < 0$ and for weakly charged surfaces we may make the approximation $\varphi_{\rm int} \approx \varphi_0 = \varphi(\sigma = 0)$. Eq.S 7 implies that the interfacial contribution generates an attractive long-ranged force between negatively charged particles and a repulsive

force for positively charged particles. We expect the converse to hold for a solvent where $\varphi_0 > 0$, i.e., positive particles should attract.

We further note that $0 \lesssim \left|z\frac{\mathrm{d}\alpha}{\mathrm{d}\psi_s}\right| \lesssim 0.25$ depending on the pH in solution. Eq.S 7 shows that when $\left|\frac{\mathrm{d}\alpha}{\mathrm{d}\psi_s}\right|$ is large, the interfacial term can be substantial, implying significant qualitative and quantitative departure from standing theories. This departure is expected to occur when the pH is in the vicinity of the pK of the ionizable groups, or more precisely, when $10^{z(\mathrm{p}H-\mathrm{p}K)}\exp(z\psi_{s,\infty}) \approx 1$. Thus, for anionic groups with $z=-1$ and nominal values of $\psi_{s,\infty} \approx -0.5$ to -6 at low and high pH respectively, we may expect $\left|\frac{\mathrm{d}\alpha}{\mathrm{d}\psi_s}\right|$, and therefore ΔF_{int}, to be large in the range of pH given by p$K + 0.2 \lesssim$ p$H \lesssim$ p$K + 2.5$ (Fig. 2d). Conversely when $\frac{\mathrm{d}\alpha}{\mathrm{d}\psi_s} \to 0$, which occurs well outside the above indicated pH range, Eq. S 7 suggests no contribution at all from the interfacial term, i.e., $\Delta F_{\mathrm{int}} \to 0$.

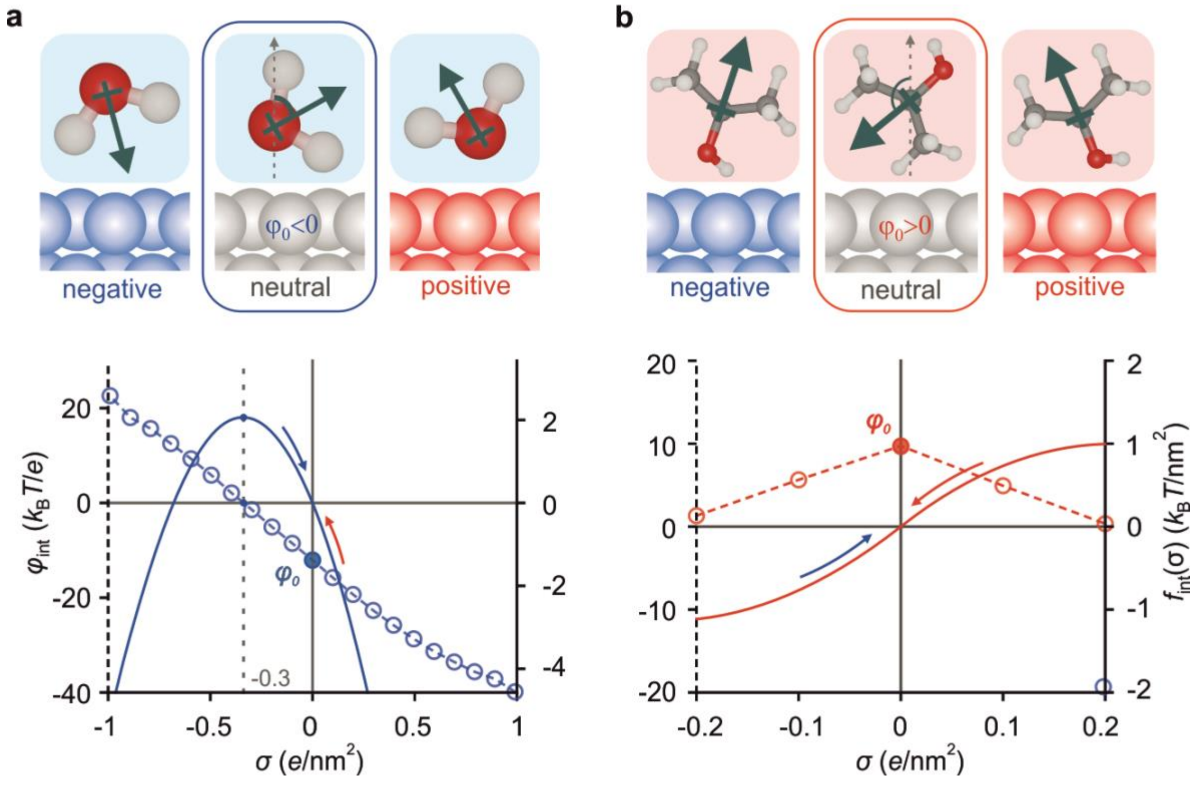

Fig. S8. Schematic depiction of the qualitative average orientation of a solvent molecule at the solid-liquid interface. (**a**) Top: Schematic representation of the dominant orientation of water molecules close to a neutral interface. On average, a molecule close to the interface points its oxygen atom slightly towards the interface and hydrogen atoms towards the bulk, giving a net dipole moment (green arrow) whose *z*-component points away from the interface and towards the bulk. Dashed vertical arrow depicts the surface normal. This symmetry-

breaking in average orientation gives rise to an interfacial electrical potential (or solvation potential) with negative sign, i.e., $\varphi_0 < 0$ (centre panel). At a positively charged surface the average molecular orientation is reinforced giving a more strongly negative value of φ_{int} (right panel). At a strongly negatively charged surface the water molecule flips around, pointing its hydrogen atoms and its dipole strongly towards the interface (left panel). The re-orientation of water at a negatively charged surface results in a gradual increase in φ_{int} with increasing magnitude of negative surface charge, going through 0 and turning positive as the hydrogen atoms increasingly point towards the interface (open symbols, bottom panel). The plots in the lower panels depict the dependence of φ_{int} on surface charge density, σ as obtained from MD simulations of SPC water at a model surface composed of oxygen atoms[6,12] (open symbols). The integral $f_{\text{int}}(\sigma) = \int_0^{\sigma} \varphi_{\text{int}}(\sigma)\text{d}\sigma$ gives the interfacial solvation free energy per unit area (solid line)[6,12]. When two like charged particles approach each other, charge regulation reduces the magnitude of the charge on the particles in general, regardless of whether the particle is positively or negatively charged. The blue and red arrows depict the impact of charge regulation on the interfacial free energy contribution in water. The approach of negatively charged particles is accompanied by a reduction in interfacial free energy implying an attraction (downward pointing blue arrow) that can mitigate and even overwhelm the electrostatic repulsion, ΔF_{el}. The converse is true for the approach of positively charged particles, where the interfacial solvation contribution implies an additional repulsion that reinforces ΔF_{el} (upward pointing red arrow). (**b**) MD simulations of isopropanol (IPA) at a model neutral oxygen atom surface show that the IPA molecule points its oxygen atom away from the surface giving rise to $\varphi_0 > 0$ (top row). Contrary to the behaviour of water at an O-atom interface, we find that regardless of whether the surface charge turns increasingly positive or negative, the IPA molecule flips around, pointing its dipole away from the surface (green arrow), giving a reduction in φ_{int} compared to φ_0. The reorientation of IPA at a charged interface thus causes φ_{int} to go through zero and turn increasingly negative as the oxygen atom points more strongly towards the surface. Note however that surface chemistry can have a substantial impact on the behaviour of the solvation potential in organic solvents[12]. Regardless, for weakly charged surfaces, typical in experiments, the behaviour of the interfacial free energy is governed by the sign and magnitude of φ_0. Assuming the O-atom surface accurately captures the sign of φ_0, we expect that f_{int} provides a decreasing, attractive contribution to the pair interaction energy (downward pointing red arrow) for the approach of positively charged particles, and vice versa for the interaction of negatively charged particles (upward pointing blue arrow). All dipole moment vectors for illustrative purposes only.

S3.3 Discussion on parameter values used in the theoretical modelling of the experimentally inferred interaction potentials

The input parameters in the theoretical model of the interfacial free energy include the radius of the particles, R, the dielectric constant of the bulk solvent phase, the concentration of monovalent salt in solution, c_0, the number density of the surface ionisable groups, and the parameter $p = z(\text{p}H - \text{p}K)$. R is known from measurement. We fix the values of all other parameters to the values displayed in Table S1 and vary the value of p to obtain calculated pair

interaction potentials characterized by values of w and x_{min}that agree with the experimentally inferred potentials.

Ionizable group density, Γ: For silica surfaces we assume $\Gamma = 1$ nm^{-2}, which is a typical value for silica[17,34]. We take $\Gamma \approx 0.1$ nm^{-2} for the COOH and NH_2 functionalized particles. Here we have assumed that chemically functionalized particles are likely to have approximately an order of magnitude lower group density than amorphous solids such as silica. The precise value of Γ is not very important to the final modelling results since a different value of Γ will require a slightly different value of p to give a similar pair potential. Given the uncertainty in pK value of at least 1-2 units, the calculated results may be regarded as not particularly sensitive to the exact value of Γ. We therefore hold Γ constant for a given material and vary the value of p in the calculations to obtain a pair potential that captures the experimentally inferred profile.

Salt concentration, c_0: All other parameters remaining constant, salt concentration strongly affects the form of the calculated pair potential by directly influencing the screening length, κ^{-1}. Values of c_0 quoted in the experimental data denote nominal salt concentrations inferred from electrical conductivity measurements of the background electrolyte solution used to produce particle suspensions (see Section S1.2). These concentration measurements may not accurately reflect *in situ* values of concentration in the experimental microscopy chamber which can be different. In general, when modelling experiments on positive particles (both in water and in organic solvents) we found that the best agreement with measurements was obtained for c_0 values in the model that were about 0.04-0.1 mM larger than the nominal, measured bulk electrolyte values (Table S1). We attribute this observation to the fact that the silica chamber walls were chemically functionalized with positively charged polyelectrolytes for all experiments with positively charged particles. This additional chemical processing is likely to alter the ionic strength of the suspension added to the chamber. However, for experiments with negatively charged particles, and for experiments at higher salt concentrations, the disparity between nominal measured concentration values and those used in the modelling procedure was generally small (Table S1). Note that experiments performed without any added salt, in “deionised” water, can in general carry larger uncertainties on the estimated ionic strength. This is due in part to CO_2 dissolution from the air, and the general susceptibility of a pure solvent to impurities from contact with other phases including experimental surfaces.

Modelling silica: For experiments on silica at various salt concentrations and pH in the range 5.5-6.5 shown in Fig. 1, we model pair interactions with a single value of p (implying a single pK value in the range 4-6.5). Clearly, this is a significant oversimplification given the variety of pKs known to describe the system[34,35]. But the implication in doing so is that the overall behaviour can be captured by a single effective pK value representing groups whose pKs lie in the vicinity of the single nominal pK. Groups with p$K > 6$, higher than the nominal effective pK, are assumed not to contribute to the interaction as they can be regarded as effectively completely discharged. Groups with more acidic pKs (p$K < 4$) are captured using a small fraction ($f_1 = 0.5\%$) of a highly acidic, permanently ionized species of pK characterised by $p_1 = \text{p}K - \text{p}H = -5$. This approach permits us to construct a coarse-grained model for silica behaviour in the experiments described in Fig. 1. Note that given the large number of poorly controlled parameters involved in describing the silica surface we do not attempt to provide calculated $U(x)$ profiles as a function of pH for silica (experiments in Fig. 2).

Modelling interactions in alcohols - salt concentration and pK *values*: In our PB electrostatics model we treat both isopropanol and ethanol as media with a dielectric constant $\varepsilon = 20$. We use a value of $\varphi_0 = +0.2$ V for the interfacial solvation potential in calculating the interfacial free energy term.

Similar to the experiments on positive particles in water, we use a value of c_0 larger by about 0.075 mM compared to the nominal experimental salt concentration. Furthermore, conversion of measured electrical conductivities to salt concentration in alcohols involves poorly controlled assumptions on the ionic species in solution and the corresponding ionic radii (see Section S1.2). As a result, we may expect that the inferred experimental salt concentrations may carry an error as large as a factor of 2. Experimental uncertainties notwithstanding, the theoretically modelled curves are based on c_0 values close to the experimentally measured nominal values (Table S1).

Note that calculations of $U_{\text{tot}}(x)$ for alcohols (presented in Fig. 5) assume the same value of $\varphi_0 = +0.2$ V for both neutral COOH and NH_2 surfaces in IPA (Fig. 5a). But there is no reason for these values to be the same, since surface chemistry can influence the magnitude and even the sign of φ_0 for a given solvent[12]. In the present study, qualitative arguments concerning the orientation of alcohols at model oxygen atom surfaces would suggest that the sign of φ_0 is preserved. But the magnitude of φ_0 could be very different for the two (real)

surfaces and this will influence the calculated $U_{\text{tot}}(x)$ curves. The calculated $U_{\text{tot}}(x)$ curves in Fig. 5 thus serve the purpose of qualitative comparison with experimental observations.

Furthermore, pK values for ionisable groups in organic solvents are not known accurately. Our previous work on charge measurements on silica in polar and non-polar solvents suggested an upward shift in the effective pK of silica of ≈3.5 units in ethanol[1]. This estimated shift was based on simple solvation energy estimates and was found to agree with experiments. Applying similar considerations to COOH and NH_2 surfaces in the experiments, we expected an upward shift of the pK of carboxylic acid groups to p$K > 8$ and a downward shift of basic NH_2 group pK values to p$K < 7$ in alcohols. The values of p obtained in the modelling procedure for COOH particles (Table S1) however imply a rather large upward shift of pK of approximately 6-7 units to about p$K = 11 - 13$ for COOH particles and not much of a shift for amino groups, i.e., p$K \approx 8$ for NH_2 groups in ethanol. Remarkably, our inferred values of pK for COOH groups and NH_2 groups in ethanol, listed in Table S1 are found to be in good agreement with the literature values for carboxylic acids such as acetic and benzoic acids in ethanol (p$K > 10$) and a range of aliphatic amines in ethanol ($7 <$ p$K < 9$)[36,37]. Note that for carboxyl particles we have attempted to match the repulsion measured in the experiment. Interestingly, from the present parameter values, the dominant contribution to the repulsive $U_{\text{tot}}(x)$ appears to stem mainly from the $\Delta F_{\text{int}}(x)$ term rather than from the $\Delta F_{\text{el}}(x)$ term as one might intuitively expect. This balance will of course alter for a (different) value of $\varphi_0 < +0.2$ V. Therefore, the present set of results provide only qualitative indications of the expected behaviour, but the trends that emerge are all physically plausible.

S3.4 Considerations in comparing the results of pair-interaction-based theory and simulation procedures with an experiment involving an ensemble of interacting particles

Pairwise additivity of interaction energies is a simplifying assumption

A significant caveat on the presented combined experiment-theory approach is that we attempt to map a collective interaction between heterogeneous particles on to a pair-interaction calculated for an isolated pair of identical particles. This procedure should at best warrant qualitative comparisons between experimentally inferred and theoretically calculated potentials as outlined in the next section. Furthermore, in our BD simulation-based modelling of the experimentally observed interactions, we assume pairwise additivity of interparticle

interactions. This is a simplifying approximation and unlikely to be rigorously true in experiments[38]. Interaction potentials inferred from our BD simulations thus represent a pair potential that could give rise to the experimentally observed $g(r)$ structure in a situation where collective interactions can be described in a strictly pairwise additive manner. Although these inferred potentials are likely to provide a good approximation of the pair potential in experiment, they cannot be taken to accurately represent the "true pair potential". Pair potentials may indeed be accessed in measurements on interactions of isolated pairs of particles as shown in Fig. S12. But in such experiments, the uncertainty on the size and properties of the individual particles in each measured pair interaction can impede quantitative comparison to a theoretical calculation. Note that in the present cluster experiments we average over a large number of particles which suggests that we may reasonably expect the size distribution of particles in the measurement to approach that of the particle sample. We further account for particle size variation by incorporating the known 'ensemble-level' size distribution as an input into the BD simulation (see Fig. S1 and Fig. S17). Accurately accounting for the particle size distribution is likely to pose a challenge in relating direct pair-potential measurements to theoretical calculations.

<u>Experimental variability in parameters of interest</u>

Next, the experiments inherently contain a degree of variability and perturbation that are hard, if not impossible to control and account for. A few of these include: (1) average particle size in an experiment which may not correspond to the nominal mean value characterizing the sample, (2) particle-to-particle variability in surface properties, such as group density, pK values and their distribution, charge density, and particle size (3) change in surface chemistry (e.g., pK) as a function of electrolyte composition (4) drifts in pH and ionic strength as a function of time, which can influence the surface properties and therefore the stability of clusters. As a result of numerous sources of drift and variability in measurements, qualitative agreement between the theoretical model and the pair potential inferred from the $g(r)$ measurements is the best that can be expected in most cases.

<u>Scope for improvement of the present theoretical model</u>

The above caveats and considerations notwithstanding, there appear to be two aspects of the present theoretical model that suggest the need for further improvement in future work:

(1) One shortcoming of the present rudimentary theoretical model is that it often suggests a strong interparticle attraction to contact under conditions where the experiments clearly display dominance of the repulsion over the attraction at short range. In general, we find that the model does well at capturing the total interaction and the appearance of minima at large separations ($x > 200$ nm). However for experiments on negatively charged particles at ionic strengths $c_0 \gtrsim 0.1$ mM, the present model of the total pairwise interparticle interaction over-estimates the magnitude of the attractive interfacial contribution, ΔF_{int}, at smaller separations, $x < 200$ nm (Fig. 2c, middle panel), often implying attraction to contact where the experiments show stable crystalline clusters with large intersurface separations. In other words, in the present model the ΔF_{int} term appears to grow too strongly in magnitude at smaller intersurface separations. Assuming that at these separations the electrostatic part of the interaction, ΔF_{el}, is accurately given by PB theory including charge regulation, the experimental observations could be seen to imply a damping of the growth of the ΔF_{int} term at shorter range. At present there is no mechanism in the model that would provide such a damping. But future improvements to the modelling approach, such as a more integrated theory of electrostatics and solvation at an interface, or alternatively, a model that incorporates aspects of the interfacial behaviour of the solvent that are not captured in present molecular simulations may resolve this discrepancy[16].

(2) Secondly, as pointed out in previous work, in calculating $U_{\text{tot}}(x)$ we observe an over-reliance of the depth and location of the minimum (i.e., w and x_{min}) on the precise input value of p (Ref 6). Currently we believe that much of this dependence could be attributed to our attempt to capture an experimentally measured average response arising from numerous sources of experimental variability and temporal drift (particle size, charge, pH, pK, salt concentration) by varying a single parameter, p, in the calculation. However, it is entirely possible that a more in-depth theoretical treatment is likely to free our calculated results from their strong dependence on p. Again, inclusion of additional effects such as the influence of ions on the interfacial solvation structure may well alter the final calculated results, possibly enabling our modelling procedure to capture experimental observations more accurately and broadly[16]. These preliminary indications provide fertile ground for the advancement of an in-depth understanding of the contribution of the interfacial electrolyte to interparticle and intermolecular interactions in solution.

S3.5 Analysing the pH response of particle cluster formation

Comparing the pH response of the well depth, w, with the qualitative indications from the theoretical model enables us to test for the presence of a key signature of charge regulation in the interfacial free energy term, as shown in Fig. 2. The expression for the interfacial free energy displayed in Eq. S 7, gives the amplitude of the ΔF_{int} term as follows: $B \propto ez\varphi_{\text{int}} \frac{\mathrm{d}\alpha}{\mathrm{d}\psi_{\text{s},\infty}} \psi_{\text{s},\infty} \Gamma R^2$.

We expect the magnitude of the well depth w to show qualitative correlation with that of $\left|\frac{\mathrm{d}\alpha}{\mathrm{d}\psi_{\text{s},\infty}}\right|$ which is expected to change substantially as a function of pH. Depending on pH, we expect $0 \lesssim \left|\frac{\mathrm{d}\alpha}{\mathrm{d}\psi_{\text{s},\infty}}\right| \lesssim 0.25$, which suggests that when $|\mathrm{p}K - \mathrm{p}H| \gg 0$, the interfacial contribution $\Delta F_{\text{int}} \to 0$, and the interparticle attraction between negative particles should vanish altogether. Note that other quantities in Eq. S 7 – namely, φ_{int} and $\psi_{\text{s},\infty}$– do depend on the surface charge density, σ, but they can be assumed not to change as *substantially* as $\left|\frac{\mathrm{d}\alpha}{\mathrm{d}\psi_{\text{s},\infty}}\right|$ as a function of pH. For anionic surface groups, σ increases in magnitude with increasing pH. The surface electrical potential $\psi_{\text{s},\infty}$ therefore increases in magnitude with increasing pH, whereas the interfacial potential φ_{int} decreases in magnitude with increasing pH for low charge densities typically encountered in experimental systems (Fig. S8). Therefore, for the purpose of this analysis we treat the product $\varphi_{\text{int}}\psi_{\text{s},\infty}$ as remaining effectively constant in relation to the large variation expected in $\left|\frac{\mathrm{d}\alpha}{\mathrm{d}\psi_{\text{s},\infty}}\right|$ as a function of pH. The dependence of $\psi_{\text{s},\infty}$ on pH is denoted as $\psi_{\text{s},\infty}(\mathrm{p}H)$ in Eq.S 15.

Figure 2d plots measured values of w as symbols together with dashed lines for the value of $\frac{\mathrm{d}\alpha}{\mathrm{d}\psi_{\text{s},\infty}}$ as given by Eq.S 9, repeated below for convenience:

$$\frac{\mathrm{d}\alpha}{\mathrm{d}\psi_{\text{s},\infty}} = \frac{-z10^{z(\mathrm{p}H-\mathrm{p}K)}\exp\left[z\psi_{\text{s},\infty}(\mathrm{p}H)\right]}{\left\{1 + 10^{z(\mathrm{p}H-\mathrm{p}K)}\exp\left[z\psi_{\text{s},\infty}(\mathrm{p}H)\right]\right\}^2} = -z\left[1 - \alpha(\psi_{\text{s},\infty})\right]\alpha(\psi_{\text{s},\infty}) \qquad \text{S 15}$$

For anionic groups where $z = -1$, we have $\frac{\mathrm{d}\alpha}{\mathrm{d}\psi_{\text{s},\infty}} > 0$, whereas for cationic groups where $z = +1$, we have $\frac{\mathrm{d}\alpha}{\mathrm{d}\psi_{\text{s},\infty}} < 0$. In the above equation for $\frac{\mathrm{d}\alpha}{\mathrm{d}\psi_{s,\infty}}$, the experimental p$H$ is known from measurement, and nominal values of pK of the ionizable groups are known for the COOH and NH_2 particles. For silica, a range of pKs of the surface groups is available from reports in the literature[34,35]. In order to estimate $\psi_{\text{s},\infty}$, the only remaining unknown in the

expression, we may refer to zeta (ζ) potential measurements which provide indicative values for $\psi_{s,\infty}$ that are however not quantitatively exact. We emphasize that $\psi_{s,\infty}$ and ζ are themselves functions of pH, which in fact underpins the overall pH dependence of $\frac{d\alpha}{d\psi_{s,\infty}}$. Details used to generate the theoretical expected curves in Fig. 2d for the three types of particles are provided below.

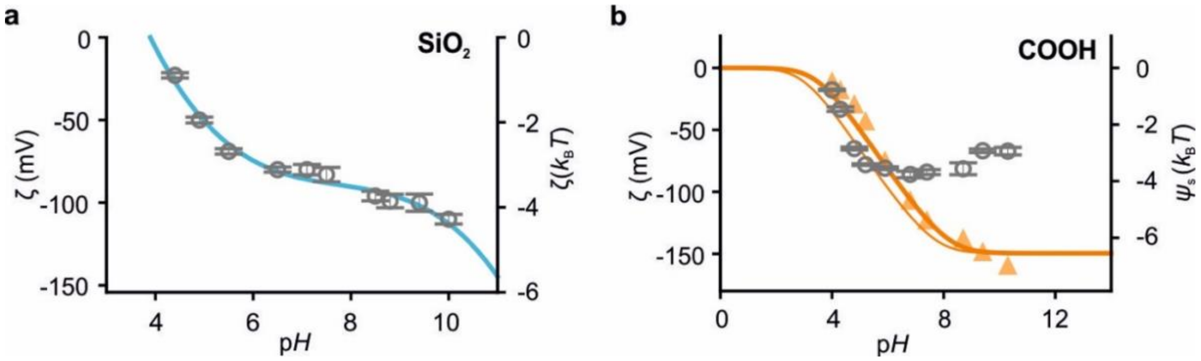

Fig. S9. ζ potentials for SiO$_2$ and COOH particles (**a**) Measured values of $\zeta(\mathrm{p}H)$ for SiO$_2$ particles used as $\psi_s(\mathrm{p}H)$ in Eq. S 15 (grey circles) The blue curve shows a fit function $\psi_s = 0.0479pH^3 - 1.1178pH^2 + 8.8668pH - 20.405$ and is provided as a guide to the eye. (**b**) Measured ζ potentials for COOH particles (grey circles). Orange triangles denote values of the surface potential, $\psi_s(\mathrm{p}H)$, used in Eq. S 15. At higher pH (>5) these values agree well with ψ_s vs. pH trends calculated for COOH particles using $\Gamma = 0.15$ nm^{-2} and pK = 4.5 (thick solid line), and pK = 5 (thin solid line.) At low pH < 5, the values are similar to both measured ζ potentials as well as the calculated ψ_s values. All ζ potentials represent mean values ± S.D. from 3 sets of measurements.

Silica particles

Because the silica surface carries a variety of ionizable species with pK values ranging from 2 to 11, we use a generalized form of Eq. S 9 applicable for a system with multiple pK values[34,35]. In particular we have

$$\frac{d\alpha}{d\psi_{s,\infty}} = \sum_i f_i \frac{d\alpha_i}{d\psi_{s,\infty}} = -z \sum_i f_i[1 - \alpha_i(\psi_{s,\infty})]\alpha_i(\psi_{s,\infty}) \qquad \text{S 16}$$

where f_i is the fractional preponderance of the ith species of pK value pK_i. As stated in Eq. S 8, the ionization probability of a group of species i may be written as

$$\alpha_i = \frac{1}{1 + 10^{z(\mathrm{p}H - \mathrm{p}K_i)}\exp(z\psi_{s,\infty})} \qquad \text{S 17}$$

In the analysis for silica shown in Fig. 2d we apply Eqs.S 16-S 17 above, setting $\psi_{s,\infty}$= ζ, the measured zeta potential values at each pH (grey circles, Fig. S9a). We further assume 7 species of ionizable surface groups of pK values p$K_1 = 3$, p$K_2 = 3.8$, p$K_3 = 5$, p$K_4 = 6$, p$K_5 = 7$, pK_6 =9, pK_7 =10 of nearly equal preponderance (i.e., $f_1 = f_2 = 0.2, f_3 = f_4 = f_5 = 0.15, f_6 = 0.1$ and $f_7 = 0.05$.)

Because the surface chemistry and properties of silica are so complex, and the values of the parameters involved not sufficiently accurately known, it is not meaningful in our view to attempt more detailed modelling of the silica response. It is interesting to note however that shallow minima in the pair-potential ($|w| \approx 2\ k_B T$), which imply attractions arising from the ΔF_{int} term in our model, remain measurable all the way up to the highest pH values. Within our model, such behaviour points to the presence of charge regulating groups with p$K > 9$ which is well known to be the case for silica[35]. Note that this broad response of silica is markedly different from particles carrying a single ionizable chemical species of well-defined pK as described further below. Finally, the present comparison of the trends for the $\frac{d\alpha}{d\psi_{s,\infty}}$ term with the measured values of well depth w, can only be regarded as qualitative as we ignore the variation of the ΔF_{el} term with pH. For acidic groups such as silanols and carboxyls, ΔF_{el} is expected to increase in magnitude with increasing pH and thus independently contribute to a lowering in magnitude of w. That the interparticle attraction is observed to persist at very high pH for silica, even in the face of an increasing electrostatic repulsion, provides further strong of evidence of a lingering, albeit diminishing attractive contribution. The observations align well with the expectation, based on our model, of an interfacial solvation contribution arising from a small population of very weakly acidic groups (p$K > 9$) known to populate the silica surface.

Carboxyl particles (-COOH groups)

Modelling the pH response of the carboxylated melamine particles permits us to attempt a more quantitative comparison of experimental trends with model predictions. We assume that the surface of our carboxyl particles may be described by a single ionizable species of p$K = 4.5$, similar to acetic acid. A quantitative calculation of $\frac{d\alpha}{d\psi_{s,\infty}}$, would require values of $\psi_{s,\infty}$ as a function of pH at a greater accuracy that that provided by ζ potential measurements. It is well known that the zeta potential does not accurately capture the value of the electrical potential at the surface of a charged particle due to the high non-linearity of the PB equation in

the regime of high potentials. We therefore use an assumed trend for the $\psi_{s,\infty}$ vs. pH relationship shown in Fig. S9 (orange triangles). This trend in $\psi_{s,\infty}$ reflects the behavior of ζ at low surface potentials as expected (Fig. S9), but $\psi_{s,\infty}$ exceeds ζ in magnitude at p$H > 5$ where the surface is expected to be strongly ionised. We point out that our assumed trend in $\psi_{s,\infty}$ vs pH agrees well with a PB calculation for particles with a group density of $\Gamma = 0.15$ nm^{-2} and pK values 4.5 and 5 (solid lines, Fig. S9).

Amino-silica particles (-NH_2 groups)

In modelling the aminated-silica particles we assume a single protonatable species of p$K = 9.5$. We assume behavior that is qualitatively analogous to COOH particles, where $|\psi_{s,\infty}|$ vs pH qualitatively follows the carboxyl response except that it is reflected and shifted to larger pH values as shown in the ζ potential measurements presented in Fig. 2b. As a result, the response in $\left|\frac{d\alpha}{d\psi_{s,\infty}}\right|$ is mirrored and shifted to larger pH values as shown in Fig. 2d. According to our model, the experimental observation for the interaction of positive particles would correspond to *enhanced repulsions* in the regime $\frac{d\alpha}{d\psi_{s,\infty}} \neq 0$. The present experiments are not sensitive enough to quantitatively measure and reliably distinguish between the strength of repulsive interactions in different experiments, but the data clearly show that attractive long-ranged minima are never observed in the pair potentials, as suggested by the model (i.e., the data are consistent with $B \propto \varphi_0 \frac{d\alpha}{d\psi_{s,\infty}} \geq 0$). Because the inferred pair potentials consistently indicate monotonic repulsive pair potentials, we have $w = 0$ for all values of pH. The above analysis demonstrates that the measured trends in w for both positively and negatively charged particles are well captured in the magnitude and sign of $\frac{d\alpha}{d\psi_{s,\infty}}$, providing a crucial test of the predictions of the interfacial solvation model and of Eq. S 7 in particular.

On a related note, it is worth pointing out that in general at low salt concentrations, we expect an upward shift of bulk pH at which clustering occurs in anionic particles, relative to the nominal pK value of an isolated, acidic ionisable group. The reason for this shift is related to the increase in magnitude of $\psi_{s,\infty}$ with increasing pH for anionic particles. The up-shift of the "clustering pH" may be viewed as a simple consequence of the elevation of electrical potential at a charged surface with a lowering of salt concentration which directly influences the bulk pH value at which the ionization probability changes strongly (i.e., $\left|\frac{d\alpha}{d\psi_{s,\infty}}\right|$ is large) as

shown in Eq.S 8. The phenomenon is sometimes referred to as a "pKa shift" has been broadly recognised and reported in a range of studies on charged molecules surfaces such as DNA, lipid bilayers and polyelectrolyte coatings[39]. For a negatively charged surface, the $\exp\,(-\psi_{\mathrm{s},\infty})$ term in Eq.S 8 and Eq. S 17 suggest that we may view the effective pH in the vicinity of the surface as lower than in the bulk. Thus as a consequence, in experiments that monitor the bulk pH, the pH value at which half-maximal ionization probability is obtained shifts to larger values. This up-shift is frequently interpreted as an apparent upward shift in pK of the groups themselves. For positively charged surfaces a converse downward shift in effective pK is expected[39]. But such shifts are primarily a consequence of a collective electrostatic effect arising from the interaction of neighbouring charges in a dense arrangement of ionizable groups, typical of macromolecules and polyelectrolytes, with the pK of the group *per se* not requiring to be different at a surface from the value in isolation. Such an 'electrostatic shift' of pK in a charged object could be contrasted with shifts in intrinsic pK values that might arise from electronic, dielectric or solvation effects in the immediate environment of the group, altering the effective pK of a group at a surface or buried in a protein[40,41].

S3.6 <u>Cluster formation in mixtures of chemically dissimilar particles</u>

In the interaction of two particles of identical radius and dissimilar surface chemistry, the prefactor of the exponential term that gives the free energy due to interfacial solvation in Eq. S 6 which may be written as follows:

$$B_{ij} \propto ezR^2 \left[\varphi_{\mathrm{int},i}\, \Gamma_i \left(\frac{\mathrm{d}\alpha}{\mathrm{d}\psi_{\mathrm{s},\infty}} \right)_i \psi_{\mathrm{s},\infty,j} + \varphi_{\mathrm{int},j}\, \Gamma_j \left(\frac{\mathrm{d}\alpha}{\mathrm{d}\psi_{\mathrm{s},\infty}} \right)_j \psi_{\mathrm{s},\infty,i} \right] \quad \text{S 18}$$

where the subscripts "$i = 1$" and "$j = 2$" denote the corresponding values of quantities for particles "1" and "2" in the interaction. Particle 1 may belong to species A whose chemistry is different from that of particle 2 of species B. In the present analysis, we ignore variations in φ_{int} between different types of surfaces, but we may assume the value remains constant for a specific set of solution conditions. We then have two major quantities that could significantly affect the magnitude of B, namely $\left(\frac{\mathrm{d}\alpha}{\mathrm{d}\psi_{\mathrm{s},\infty}}\right)$ and $\psi_{\mathrm{s},\infty}$, each characterising a different particle of the two engaged in a pair interaction. Of the two quantities, the one that displays the largest variation depending on solution conditions is $\left(\frac{\mathrm{d}\alpha}{\mathrm{d}\psi_{\mathrm{s},\infty}}\right)$.

Thus, when the values of $\left(\frac{\mathrm{d}\alpha}{\mathrm{d}\psi_{\mathrm{s},\infty}}\right)$ for both species (and implicitly, values of $\psi_{\mathrm{s},\infty}$for both species) are reasonably large, we expect B to be large for both cross-species (B_{ij}) and same-species (B_{ii}, B_{jj}) pair interactions. Therefore particle clusters that form in mixtures of different types of particle should contain both species. Similarly, when $\left(\frac{\mathrm{d}\alpha}{\mathrm{d}\psi_{\mathrm{s},\infty}}\right) \to 0$ for both species (typically occurring at extremes of pH) and for similar values of $\psi_{\mathrm{s},\infty}$, clusters form neither in pure suspensions nor in mixtures of the two species, since both terms in Eq. S 18 are small. Under solution conditions where, e.g., $\left(\frac{\mathrm{d}\alpha}{\mathrm{d}\psi_{\mathrm{s},\infty}}\right)_2$ is small – i.e., the value of $\left(\frac{\mathrm{d}\alpha}{\mathrm{d}\psi_{\mathrm{s},\infty}}\right)$ of particle #2 in the interaction (e.g., COOH particles in Fig. 4) is small - same-species clusters in species B generally do not form. This is because $\left(\frac{\mathrm{d}\alpha}{\mathrm{d}\psi_{\mathrm{s},\infty}}\right)_2 \to 0$ for both partners in a COOH pair at the relevant pH, which snuffs out B, and therefore the attractive interfacial term altogether. But the value of $\psi_{\mathrm{s},\infty,2}$ can nonetheless be large enough to produce a substantial perturbation of surface potential on particle 1 of species A (SiO_2), since $\Delta\psi_{\mathrm{s},1} \propto \psi_{\mathrm{s},2}$ (Ref. 7). This perturbation of electrical perturbation can couple with a significant $\left(\frac{\mathrm{d}\alpha}{\mathrm{d}\psi_{\mathrm{s},\infty}}\right)_1$ value on particle #1 to give a net B_{12} value that is large enough for an experimentally observable (and favourable) total interfacial free energy contribution. In other words, under specific conditions, the quantities in one of the terms in Eq. S 18 can remain large enough to give a substantial total B_{12} value despite the fact that the other term tends to zero. This can manifest as an attraction and cluster formation for dissimilar negatively charged particles in water in a situation where one species forms clusters on its own (species A in our example) and the other does not (species B). Thus, a reasonable value of $\left(\frac{\mathrm{d}\alpha}{\mathrm{d}\psi_{\mathrm{s},\infty}}\right)$ on one species of particle can be sufficient for an attractive pair interaction between dissimilar particles. This results in an 'OR' type of "truth-table" for a binarised value of $\left(\frac{\mathrm{d}\alpha}{\mathrm{d}\psi_{\mathrm{s},\infty}}\right)$ characterising cluster formation in mixtures of particles, where "0" represents $\frac{\mathrm{d}\alpha}{\mathrm{d}\psi_{\mathrm{s},\infty}} \to 0$ and "1" implies $\frac{\mathrm{d}\alpha}{\mathrm{d}\psi_{\mathrm{s},\infty}} > 0$ for anionic particles. Note that we omit detailed discussion of the influence of surface potential values, $\psi_{\mathrm{s},\infty,1}$ and $\psi_{\mathrm{s},\infty,2}$, on this set of observations because the measured ζ potentials indicate similar values of $\psi_{\mathrm{s},\infty}$ for both species, particularly in the strongly cluster-forming range of pH 4-8.

S3.7 Protein pair-interaction modelling and calculation of a protein phase separation response as a function of pH and salt concentration

Here we describe an analysis performed in order to obtain a qualitative indication of the ability of a system of identical, electrically charged protein molecules to phase separate depending on the pH and salt concentration in solution. In this analysis we regard droplet formation in proteins as similar to cluster formation in colloidal particles. Analogous to the observations on colloidal interactions we assume that a net attraction in the pair interaction energy is required for individual protein molecules to overcome translational entropy and coalesce into a separate droplet-phase. We consider two different total interaction potentials, $u(x)$: the "canonical DLVO potential" $u_{\mathrm{DLVO}}(x)$, and our proposed pair interaction potential that includes the interfacial solvation contribution to the DLVO interaction energy, i.e., $u^*_{\mathrm{tot}}(x)$.

We consider two spheres of radius $R = 2.5$ nm carrying 5 ionisable Glutamic acid residues ($\mathrm{p}K = 4.5$) and calculate the distance dependent pair interaction energy using a procedure identical to that used for colloidal particles (see SectionS3.2). The radius and net charge of the spheres in this analysis are characteristic of but not identical to the yeast wild-type protein Sup35 which has recently been shown to undergo phase separation and carries a net excess of 6 acidic amino acids (aspartic and glutamic acids) over the number of basic side chains (lysine and arginine) in the domain that controls its phase segregation response [42].

Thus we calculate both $u_{\mathrm{DLVO}}(x) = u_{\mathrm{vdW}}(x) + \Delta F_{\mathrm{el}}(x)$ as well as $u^*_{\mathrm{tot}}(x) = u_{\mathrm{DLVO}}(x) + \Delta F_{\mathrm{int}}(x)$ as previously described for colloidal particles and outlined in Section S3.2. We approximate the vdW interaction energy using the expression for interacting spheres of radius $R \sim x$. Thus we have: $u_{\mathrm{vdW}}(x) = -\frac{A_{\mathrm{H}}}{12}\frac{R}{x}$, where we take the Hamaker coefficient $A_{\mathrm{H}} = 3\ k_{\mathrm{B}}T$ in line with the 3-10 $k_{\mathrm{B}}T$ range expected for proteins at $T = 298\mathrm{K}$ (Ref. 43). We use the sign and magnitude of $u(x)$ at small separations as an indication of the presence or absence of an attractive interaction required to trigger phase segregation or droplet formation in solution. In particular, we examine the value of $u = u(x = 1\ \mathrm{nm})$ in scenarios corresponding to $u_{\mathrm{DLVO}}(x)$ and $u^*_{\mathrm{tot}}(x)$, i.e., both with and without the interfacial free energy contribution. In particular, $u_{\mathrm{vdW}} = u_{\mathrm{vdW}}(x = 1\ \mathrm{nm}) \approx -0.6\ k_{\mathrm{B}}T$. Note that an inter-protein spacing of $x = 1$ nm corresponds to a concentration of protein within the droplet of $c_{\mathrm{drop}} \approx 5$ mM, which is a typical value reported in biological phase separation studies[44]. Fig. 6 and Fig. S10 displays two-dimensional plots of both u_{DLVO} and u^*_{tot} as a function of pH and salt

concentration for the two interacting spheres. A change of sign in u from net repulsive ($u > 0$, red) to attractive behaviour ($u < 0$, blue) is indicative of the possible onset of phase separation.

But clearly the sign of the interaction energy, u, is not the only determinant of phase separation. Although it may be expected that an attractive pair potential is a necessary condition for phase separation, it is by no means sufficient. In order for an attractive pair interaction potential to dominate the overall phase behaviour and result in observable phase separation, the total interaction energy experienced by a molecule within a droplet would need to outweigh the loss in entropy associated with its state in free solution. The (unfavourable) entropic contribution to the free energy for a molecule moving from bulk solution into the droplet may be estimated as $+k_\mathrm{B}T\ln\left(\frac{c_\mathrm{drop}}{c_\mathrm{bulk}}\right)$ per molecule, where c_drop denotes the concentration of protein in the droplet/condensate phase and c_bulk denotes the concentration of protein in bulk solution.

In other words, at the boundary of stability, we require the chemical potential of the protein in the two phases to equal, i.e.,

$$\mu_\mathrm{drop} = \mu_\mathrm{bulk} \qquad \text{S 19}$$

which implies

$$u_\mathrm{drop} + k_\mathrm{B}T\ln(c_\mathrm{drop}) = k_\mathrm{B}T\ln(c_\mathrm{bulk}) \qquad \text{S 20}$$

Here u_drop is the total (stabilizing) free energy of interaction of a single molecule of protein in the droplet state relative to the bulk state. As a consequence, when stable droplets are observed we may expect the following condition to hold:

$$u_\mathrm{drop} < k_\mathrm{B}T\ln\left(\frac{c_\mathrm{bulk}}{c_\mathrm{drop}}\right) \qquad \text{S 21}$$

Taking typical values of $c_\mathrm{bulk} \approx 10 - 100$ μM and $c_\mathrm{drop} = 5$ mM gives $\frac{c_\mathrm{drop}}{c_\mathrm{bulk}} \lesssim 500$ (Ref. 44). Thus if the energetic contribution satisfies $u_\mathrm{drop} \lesssim \ln\,(500) \cong -6.2\ k_\mathrm{B}T$, we may expect phase segregation to occur. We point out that in the present analysis, c_bulk is the concentration of protein in the bulk solution at equilibrium with the droplet phase, but this value could be lower than the starting nominal concentration of protein in the experiment.

Importantly, a molecule in a droplet condensate engages in polyvalent interactions with more than one nearest neighbour. Therefore u_drop is not the same as the pair interaction energy u (Fig. S10). In order to estimate u_drop, we assume a coordination number of ≈ 10 per molecule (which is the average of number of nearest neighbours in body-centered and face-centered close-packing) (Fig. S10i). We also assume that the total interaction energy for a molecule in the droplet state can be regarded as pairwise additive to the first approximation.

Thus a total stabilizing interaction energy of $u_{\mathrm{drop}} = -6.2\ k_{\mathrm{B}}T$ for a single molecule in the droplet state corresponds to a pair interaction energy of $u \approx -0.62\ k_{\mathrm{B}}T$ for a single pair of molecules. We may therefore expect phase separation in the region of the diagram where $u < -0.62\ k_{\mathrm{B}}T$.

Fig. S10e displays a coexistence curve in the total interaction energy diagrams corresponding to $\mu_{\mathrm{drop}} = \mu_{\mathrm{bulk}}$, which in this case denotes the condition $u_{\mathrm{c}} = -0.63\ k_{\mathrm{B}}T$. The coexistence curve separates the parameter space into a region where phase separation (droplet formation) may be expected to occur ($u < u_{\mathrm{c}}$) and another where the suspension may be expected to remain uniformly dispersed ($u > u_{\mathrm{c}}$). Comparing the coexistence curves in the DLVO and in the interfacial solvation free energy diagrams, we note a marked qualitative difference between the two predicted behaviours (Fig. S10g, h). Remarkably the interfacial solvation model is able to qualitatively capture the recently reported pH and salt concentration dependent phase separation behaviour for the wild type Sup35 protein (Fig. S10h, k, l). Here, similar to other observations in the literature, droplet formation is enhanced as the salt concentration is lowered. Droplet formation is also expected to be significant at lower pH, but in fact occurs at pH values significantly higher than the pK values of the ionisable groups, where the molecules retain significant amounts of negative charge and the electrostatic repulsion term ΔF_{el} remains substantial (Fig. S10a,f). Droplet formation vanishes at high pH where the electrostatic repulsion overwhelms all other attractive contributions.

For the same value of $u = u_{\mathrm{c}}$, the DLVO model would suggest no phase separation at all (Fig. S10g, j). The sole source of intermolecular attraction in the DLVO model is the vdW contribution to the pair interaction, $u_{\mathrm{vdW}} \approx -0.6\ k_{\mathrm{B}}T$, which we assume to remain constant regardless of pH and salt concentration. In this analysis the vdW contribution alone appears insufficient to overcome the entropy loss due to droplet formation. However, assuming for argument that the vdW contribution were larger and indeed sufficient to sustain a phase separation response on its own (say $u_{\mathrm{vdW}} \approx -1\ k_{\mathrm{B}}T$), then the region corresponding to $u \approx 0$ (white band in Fig. S10d) in the DLVO interaction energy diagram presents an approximate contour of a possible coexistence curve. Clearly the pH -salt dependence of such a coexistence curve, is very different to the experimental observations (Fig. S10d). Under the influence of a DLVO type of interaction, phase separation would be expected to occur at higher salt concentrations, regardless of pH, which is not supported by experimental observations on proteins such as Sup35 and Ddx4[42,44] (Fig. S10l).

Finally it is worth noting that for particles carrying mixed acidic and basic groups of different pK values, such as proteins, where the interacting objects may have a nominal net charge of a given sign, but in fact carry non-uniform patchy regions of charge of either sign, the overall pH and salt concentration dependence of ΔF_{int}, and therefore the phase separation behavior, could be more complex. Here, the sign of $\frac{\mathrm{d}\alpha}{\mathrm{d}\psi_{\mathrm{s},\infty}}$ for the local charged patches or groups is a decisive factor, and the requirement for $\left|\frac{\mathrm{d}\alpha}{\mathrm{d}\psi_{\mathrm{s},\infty}}\right|$ to be large may be met at more than one pH value or over a range of pH values. The present analysis however focuses on the simplest of systems where the objects carry ionizable groups of the same qualitative nature, i.e., acidic.

Ultimately a model of interactions including amino acid residue level detail may be required to provide quantitative calculations of the behaviour expected of proteins within the interfacial solvation model. Future work could examine the use of molecular simulations that incorporate both explicit water and charge regulation of ionisable protein groups in order to construct more accurate models of interaction free energies for a variety of molecular systems[45]. Note that the charge-asymmetry of the interfacial solvation contribution we describe by no means precludes coacervate formation in positively charged molecular systems containing aromatic rings. Here, condensate formation is believed to be driven by an attractive cation-π interaction[46].

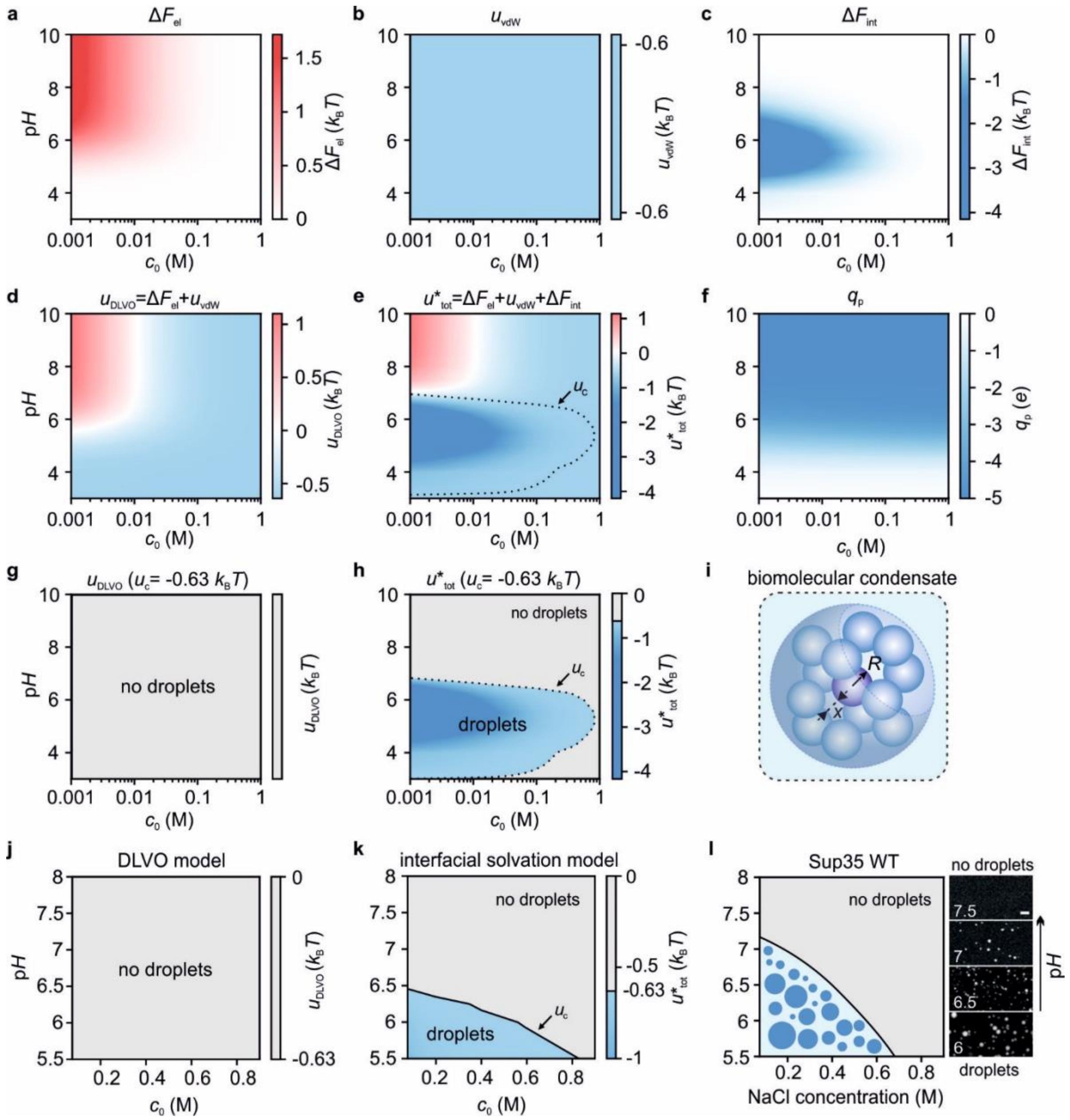

Fig. S10. (a-c) Plots of the various contributions to the pair interaction energy $u^*_{\mathrm{tot}}(x)$ for a pair of negatively charged protein molecules, calculated as a function of pH and salt concentration c_0 at an intermolecular separation $x = 1$ nm. With the interaction energy referenced to zero at $x = \infty$, positive and negative values of interaction free energies at $x = 1$ nm are indicative of repulsion or attraction respectively. **a**: The electrostatic free energy, ΔF_{el}, is substantial and positive particularly for p$H > 5$, implying a repulsive Coulombic interaction, as expected. **b**: the attractive vdW interaction energy, $u_{\mathrm{vdW}} = -0.6\ k_{\mathrm{B}}T$, which we assume is not a function of pH or salt concentration. **c**: The interfacial solvation free energy, ΔF_{int}, displays a substantially negative contribution in the range of pH 4 to 7.5 and at lower salt concentrations. (**d**) Total interaction energy $u_{\mathrm{DLVO}} = \Delta F_{\mathrm{el}} + u_{\mathrm{vdW}}$ expected from a DLVO model of interactions. (**e**) Total interaction energy $u^*_{\mathrm{tot}} = u_{\mathrm{DLVO}} + \Delta F_{\mathrm{int}}$ expected following inclusion of the interfacial solvation contribution in the total interaction energy. Black dotted contour denotes the coexistence curve given by $u_{\mathrm{c}} = -0.63\ k_{\mathrm{B}}T$. (**f**) Calculated protein charge, q_{p}, shows that the charge on the molecules remains substantially negative over a wide range pH values. (**g, j**) The DLVO model suggests no phase

separation regardless of pH and c_0 with $u_\mathrm{c} = -0.63\ k_\mathrm{B}T$, as expected. Setting $u_\mathrm{c} > -0.5\ k_\mathrm{B}T$ would in fact support the prediction of phase separation based on this interaction energy diagram, however the contour of the resulting coexistence curve would be significantly different from experimental observations (see panel (l)), as is evident from the 'white region' in the plot in (d). (**h, k**) Expected dependence of phase separation on pH and c_0 from the interfacial solvation model. (**i**) Schematic depiction of a phase-separated biomolecular condensate (droplet) showing a single molecule of radius R engaged in interactions with 12 nearest neighbours. (**l**) Measured pH and salt concentration dependence of the phase separation of yeast wild-type protein Sup35. The region shaded blue depicts conditions that support the formation of droplet condensates. Fluorescence images of condensate formation at a salt concentration of ≈ 0.1 M depict marked reduction and disappearance of droplets at p$H > 6.5$. Experimental data reproduced with permission from Ref. 42.

We conclude with a few final comments embedding our observations in the broader context of the history of experimental investigation of the interaction between charged objects in solution and the conventional expectation that PB theory, which always predicts repulsions, is expected to hold, especially in solutions containing low concentrations of monovalent ions. Our calculation of the pair interaction potential that arises *when charge regulation couples to the behaviour of interfacial water (*whose properties we have obtained from atomistic MD simulations) shows that there are conditions where like charged particles attract as well as a range of conditions where they repel in accordance with the canonical expectation. From the perspective of the model therefore, it is expected that experiment can and does often show full agreement with PB theory: e.g., our own experiments on positively charged particles in water, as well as negatively charged particles at pH values reasonably far away from the pK, show behaviour that one would consider in full agreement with PB theory. Note also that it comes as no surprise that theoretical studies that have extensively examined the topic (without considering the effects that we account for in combination) have not reported the possibility of attraction between like-charged particles.

To sum up the observations in our study, the ability to *qualitatively* alter the attractive or repulsive nature of an interparticle interaction at long range by tailoring the surface properties of the particle points to an interfacially governed contribution to the total interaction free energy of a pair of particles in solution. The amenability of the interaction to tuning using the solution pH and particle pK strongly suggests that charge regulation plays a defining role in the magnitude of the free energy contribution. The interparticle interaction further displays a stark qualitative asymmetry with respect to the charge on the particle such that *the sign of the particle charge determines the sign of the force* at long range. Importantly, the type of charge-reversal asymmetry observed in water can be inverted through the use of a solvent with

different structural and interaction properties. Our proposed mechanism invoking the excess free energy associated with solvent orientation at an interface captures all these features and explains the experimental observations. The remit of the problem specifically covers pH and salt-concentration dependent cluster or phase formation, as reported in a range of chemical and biochemical processes. Examples of relevant assembly processes range from gelation, coagulation, crystallisation and biological phase segregation to histone-modulated packaging of DNA, virus binding to sulfonated carbohydrates on host cells, biofouling, or indeed any experimental situation where counterintuitive attractions are implicated between suspended entities carrying net electrical charge of the same sign (negative in the aqueous phase).

S4 Supplementary Results

S4.1 Interactions in silica particles in water without prior treatment with NaOH

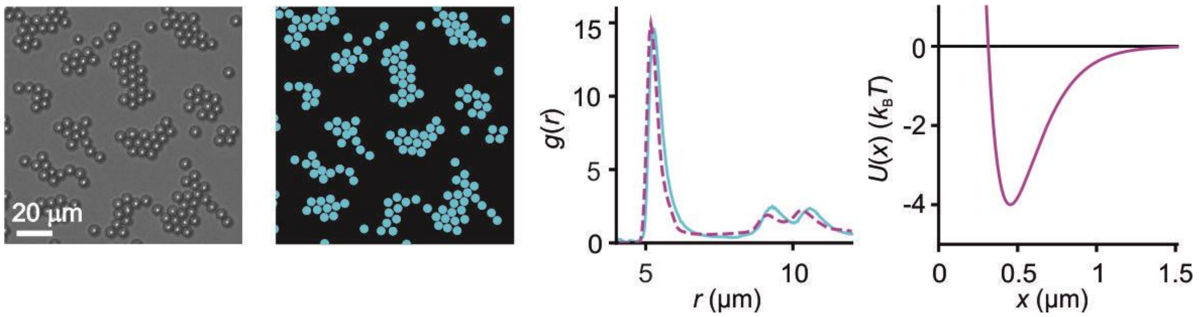

Fig. S11. Interaction of silica particles in deionised (DI) water prepared without prior NaOH treatment. (Left) Experimental and digitised microscopy images of clusters of silica colloids in DI water that had no prior NaOH treatment, as is standard practice for the experiments in this work (see Section S1.1). The experimental protocol here involved washing the silica particles 5 times in DI water, leaving for 12 hours in DI and then washing again 5 times before being resuspended in the experimental cell. Particles are represented as coloured discs of uniform diameter $2R$ on black background. (Right) Overlays of experimental and simulated $g(r)$(solid blue and dashed magenta coloured lines respectively). Also shown is the corresponding inferred pair interaction potential $U(x)$ of the form of Eq. S 6, which is used as an input to a BD simulation to match the experimental data. Parameters corresponding to the inferred $U(x)$ curve are $A = 2137.051$, $B = -1903.887$, $\frac{\kappa_2}{\kappa_1} = 0.95$, and κ_1^{-1} =136 nm.

S4.2 Direct measurements of interaction potentials for pairs of isolated particles

We have further measured the interaction potential between isolated pairs of particles in solution. Fig. S12 presents measurements of the pair potential by single particle tracking of two interacting COOH particles.

In order to directly measure the pair interaction potential $U(x)$ we focused our attention on the behaviour of isolated, diffusing pairs of particles. We define an isolated pair as one where the particles are several particle diameters away from all other neighbouring particles. We examined particle pairs in experiments on COOH particles similar to those shown in Fig. 1. Images were recorded at a frequency of 10 fps using exposure times less than 1 ms. Frame-by-frame tracking of particle centres over a total measurement period of 50 seconds permitted us to construct a histogram of interparticle distances, r. We then converted the measured radial number density function, $N(r)$, into a radial probability density function, $P'(r) = \frac{N(r)}{2\pi r \Delta r}$,

where Δr is the width of a bin in the histogram. We work with a rescaled probability density function $P(r) = \frac{P'(r)}{P'_{\max}(r)}$ where $P'_{\max}(r)$ is the maximum value of the measured radial probability density function, $P'(r)$. Subsequently, we replaced the interparticle distance variable, r, with the intersurface separation, x, using the relation $x = r - 2R$, where $2R = 5.29$ μm is the nominal average diameter of the COOH particle sample. We then obtained a measured pair potential $U(x)$ by Boltzmann inversion of the intersurface probability density function, i.e., $U(x) = -k_{\mathrm{B}}T\ln\,[P(x)]$. Error bars on the free energy values for each of the measured points are derived from a shot-noise estimate given by $\sqrt{N(r)}$ for each bin of the number density histogram. Note that all measurements of inter-separation, x, in such pair-potential measurements could contain a large systematic uncertainty, of approximately 100 nm for COOH particles, which arises from the absence of information on the true particle radii in the interacting pair of interest.

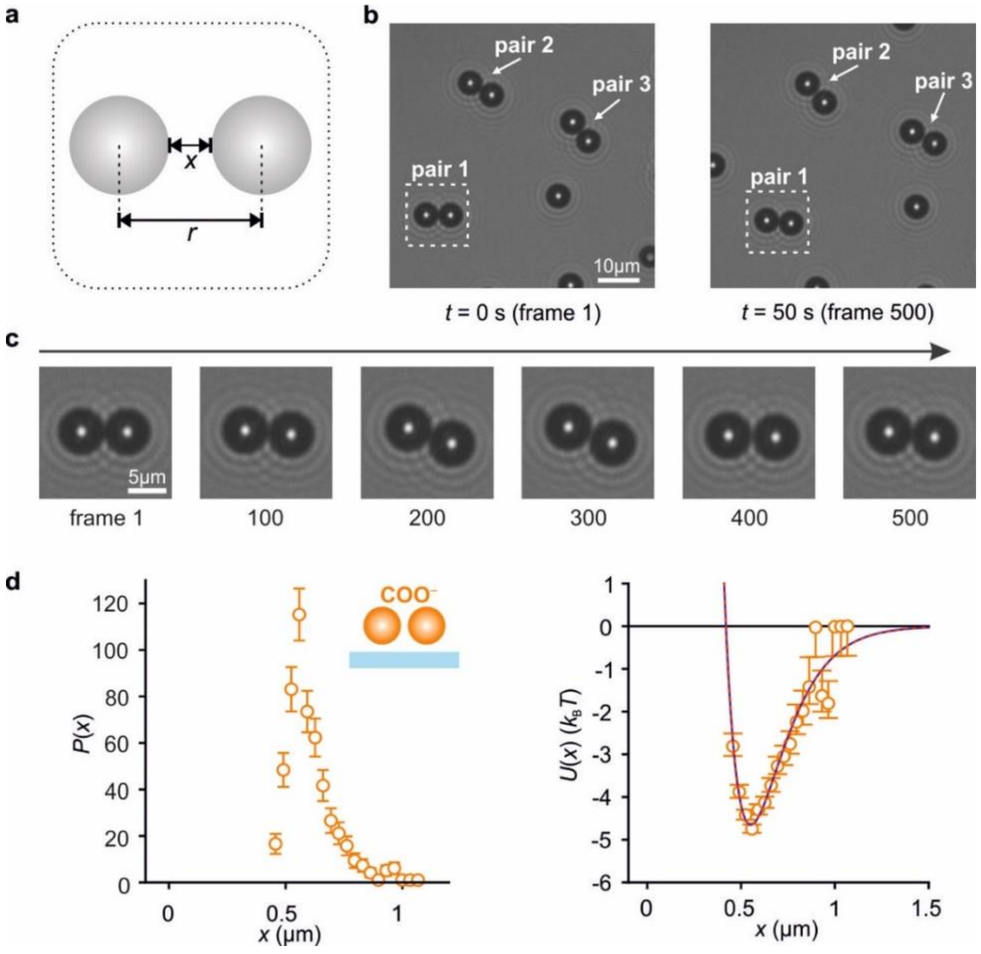

Fig. S12. Direct measurement of pair-interaction potentials for COOH particles. (**a**) Schematic of a pair of colloidal particles with center-to-center distance and intersurface separation of a particle denoted by r and x respectively. (**b**) Experiments on a low concentration of particles (approximately factor 2 lower than for the cluster experiments) under solution conditions that display particle clustering often contain isolated, strongly interacting "bound pairs" of particles located several micrometers from other neighbouring particles as indicated (top) (**c**) Snapshots in time of an interacting pair of particles (middle panel) over a period of 500 frames corresponding to 50 s. (**d**) Probability density distribution, $P(x)$, (left panel) of the intersurface separation, x, acquired over a typical period of 50 s, and measured pair-interaction energy profile, $U(x)$, (right panel) for a single pair of COOH particles. The measured $U(x)$ data (orange symbols) are presented together with fits to Eq. S 6. In one case we treat A, B and the screening length κ_1^{-1} as free parameters and hold the ratio of the two screening lengths fixed, i.e., $\frac{\kappa_2}{\kappa_1} = 0.95$ (blue dashed line). In the second case, we treat A, B and the ratio of the two screening lengths $\frac{\kappa_2}{\kappa_1}$ as free parameters, holding the Debye length fixed at $\kappa_1^{-1} = 129$ nm, which is the value corresponding to the experimentally measured ionic strength (red dashed line). The fit parameters in each case are as follows: Fit #1 (blue dashed line): $A = 6403$, $B = -5441$, $\kappa_1^{-1} = 129 \pm 9$ nm, $R^2 = 0.83$; Fit #2 (red dashed line): $A = 7139$, $B = -6174$, $\frac{\kappa_2}{\kappa_1} = 0.96 \pm 0.13$, $R^2 = 0.91$. Fit parameter values averaged over 11 different COOH pairs yield: Approach #1: $A = 14882$, $B = -11789$, $\kappa_1^{-1} = 114 \pm 8$ nm, $R^2 = 0.87$, and Approach #2: $A = 1118707$, $B = -1117949$, $\frac{\kappa_2}{\kappa_1} = 0.96 \pm 0.06$, $R^2 = 0.83$. Error bars on $P(x)$ and $U(x)$ values for each of the measured points are derived from a shot-noise estimate given by $\sqrt{N(r)}$, as described in Section S4.2.

S4.3 Optical artifacts in particle tracking do not influence the measurements

Prior work on the measurement of pair interaction potentials between colloidal particles has demonstrated that conventional particle tracking algorithms, which make use of centroid or radial symmetry methods to determine particle location, are prone to errors in particle localisation[47-49]. Errors may be expected especially when particles are in very close proximity with one another and the intensity distribution around each particle is affected by that of its neighbour.

Baumgartl *et al.* pointed out that overlapping of particle images distorts positional information on particles, giving rise to a subtle distortion of a measured interaction potential corresponding to apparent measured minima of 0.2-0.5 $k_{\mathrm{B}}T$ depth[47]. We measure attractions in the range of 5 $k_{\mathrm{B}}T$ where the experimental uncertainty on inferred depth is itself of the order of 1 $k_{\mathrm{B}}T$. Therefore at the outset, we expect that any contribution from optical artifacts to our estimate of minima depths is smaller than the depth of potential measured in most cases, and also smaller than our experimental uncertainty. Furthermore from the literature, the spurious appearance of a minimum in an otherwise purely repulsive interaction would seem to be

relevant for smaller particles (2 μm diameter or less). The apparent potential minimum results from a redistribution of probability density as a function of separation. For larger particles of 5 μm diameter as in our work, Ref. 49 presents a calculation of the error in position determination using conventional centroid based and radial symmetry based tracking methods. Applying the theoretically expected correction as well as the algorithm provided in Yücel *et al.* to correct our particle localisation measurements we obtain the results shown in Fig. S13 below[49]. As expected the corrections due to optical effects are within the errors of the measurement.

The next particle tracking correction method we applied to our data followed the approach outlined in Ref 48. Here, an image of an isolated particle and its associated diffraction pattern is used to construct a series of composite images of particles at a known separation r_{t}. The 'apparent' separation r_{m} is then determined by the same image analysis method (the radial symmetry method) used to analyse experimentally acquired images, and the difference $r_{\mathrm{t}} - r_{\mathrm{m}}$ found as a function of r_{t} (see Fig. S13c). The maximum error in the inferred interparticle distance $r_{\mathrm{t}} - r_{\mathrm{m}} = 37$ nm occurs at an inter-particle separation corresponding to the distance of nearest approach $r_{\mathrm{t}} = 2R$. The functional form of the correction shown in Fig. S13c was linearly interpolated and applied to correct the inter-particle distances measured in an experiment of two interacting COOH particles. These corrected distances were then used to obtain corrected pair interaction potentials $U(x)$ (see Fig. S13b), using the method outlined in the previous section.

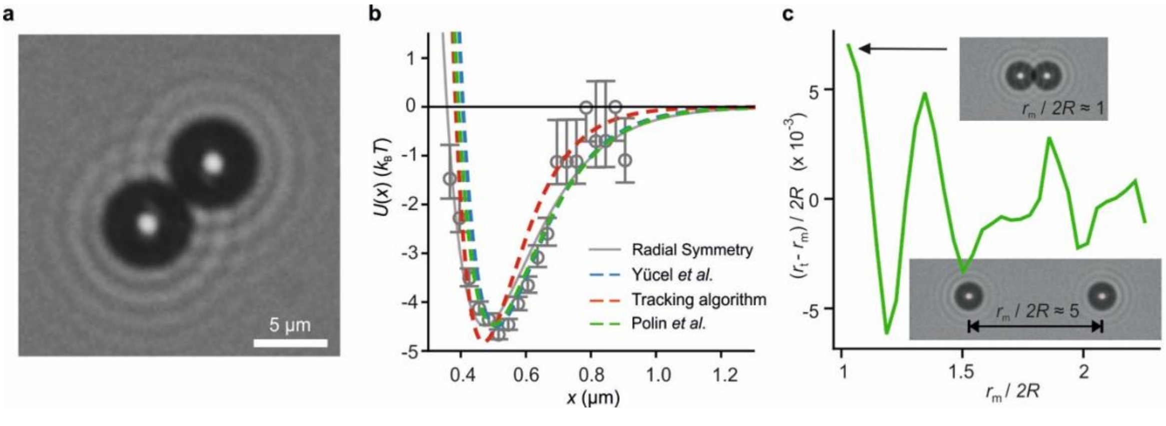

Fig. S13. Application of particle tracking corrections and their effect on the measured pair interaction potentials $U(x)$. (**a**) Digital video microscopy snapshot of a pair of interacting COOH particles, diameter $2R$ = 5.29 μm. The particle images are characterised by a bright intense region at the particle centers, surrounded by a dark ring and associated diffraction pattern. (**b**) Measured pair interaction potentials, $U(x)$, calculated using the

method outlined in Section S4.2, and for which the particle center coordinates were determined via the TrackNTrace particle tracking code employing the radial symmetry method[4] (grey symbols, accompanied by grey fit curve) i.e. the particle tracking method used in all experiments in this work. Fits to $U(x)$ data obtained by applying a correction to the measured distances according to Ref. 49 (blue dashed line), applying the particle tracking algorithm provided in Ref. 49 to our experimental data (red dashed line) and applying a different correction to r as described in Ref. 48 (green dashed line).). All fits of $U(x)$ data are to the form to Eq. S 6 and were performed setting $\frac{\kappa_2}{\kappa_1} = 0.95$ in all cases. The parameters are as follows: (1) $A = 5596$, $B = -4779$, $\kappa_1^{-1} =$ 113nm (grey) (2) $A = 13926$, $B = -11363$, $\kappa_1^{-1} = 100$ nm (blue) (3) $A = 24819$, $B = -19743$, $\kappa_1^{-1} =$ 84 nm (red), and (4) $A = 10166$, $B = -8424$, $\kappa_1^{-1} = 105$ nm (green). The fit error in the quoted Debye lengths, κ_1^{-1}, does not exceed 8 nm for all cases. Good agreement between the various curves shows that particle-tracking correction methods have a negligible effect on the inferred well depth w and x_{min} values of the underlying pair interaction potential. Error bars on $U(x)$ values for each of the measured points are derived from a shot-noise estimate given by $\sqrt{N(r)}$, as described in section S4.2. (**c**) Plot of the expected difference $r_{\mathrm{t}} - r_{\mathrm{m}}$ between the true and measured interparticle separation as a function of r_{t}, for our experimental system, using the method of Ref. 48.

S4.4 <u>The long-range attraction is not influenced by surface charge on the neighbouring coverglass surface</u>

In order to examine the role played by the neighbouring, like-charged bounding surface we performed measurements on clusters and bound pairs of particles using a coverglass (as shown in Fig. S3) coated with a thin metal layer that masks the surface charge. We used conformal coatings of 5 nm Chrome as well as surfaces patterned with Cr-Au films (see Fig. S14). Cluster formation occurs regardless of the magnitude and spatial distribution of charge on the underlying surface. Measurements on clusters in the vicinity of metal films yielded pair-potentials whose parameters (Debye length κ_1^{-1} and depth of minimum w) agreed within error with measurements performed using uncoated glass surfaces (Fig. S14a).

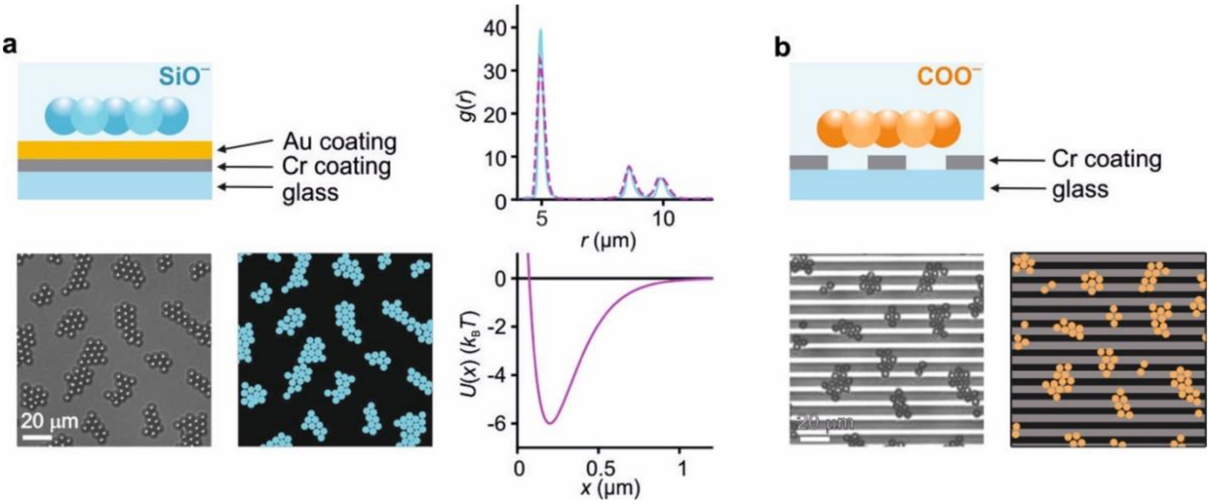

Fig. S14. Examining the effect of coating the underlying coverglass of the experimental cell with thin metal films. (**a**) Schematic representation of the experimental system system displaying a cluster of SiO_2 particles above the underlying coverglass which is coated with a 5 nm thick chrome (Cr) film followed by a 10 nm thick gold (Au) film (top), in order to suppress any electrostatic contribution from the glass, similar to Ref. 48. Experimental and digitised microscopy images of silica colloidal clusters formed above the gold coated surface. Particles are represented as coloured discs of uniform diameter $2R$ on a black background (bottom left). Overlays of experimental and simulated $g(r)$ data (solid blue and dashed magenta coloured lines respectively) (top right). Corresponding inferred pair interaction potential $U(x)$, used as input to a BD simulation to match the experimental $g(r)$ (bottom right). $U(x)$ has the form of Eq. S 6 where $A = 571.1803$, $B = -554.6975$, $\frac{\kappa_2}{\kappa_1} = 0.95$, and $\kappa_1^{-1} =$124 nm which corresponds to the experimentally measured salt concentration. These values maybe compared with a direct pair-potential measurement of $U(x)$ in the same experiment presented in Extended Data Fig. 1d. Here we treat A, B and the screening length κ_1^{-1} as free parameters and hold the ratio of the two screening lengths fixed, i.e., $\frac{\kappa_2}{\kappa_1} = 0.95$. The fit parameters are $A = 928$, $B = -871$, and $\kappa_1^{-1} = 103 \pm 7$nm ($r^2 = 0.83$ for the fit). (**b**) Schematic representation of the experimental system in which the coverglass surface is patterned with a 5 nm thick Cr film etched to create a surface with alternating metallised and non-metallised stripes of 5 μm width. (top). Experimental and digitised microscopy images of COOH colloidal clusters formed above the patterned Cr surface (bottom).

S4.5 Ionic strength dependence of cluster formation for COOH particles in aqueous solution

The general trends observed for interactions of COOH particles in response to the variation in solution ionic strength resemble those for SiO_2 particles: the attractions appear strongest at the lowest salt concentrations and gradually weaken with increasing salt concentration (Fig. S15 and Fig. S25c). However, the surface functional group density also

plays an important part in the effect, as suggested by Eq. S 7. Carboxylated melamine resin particles in our experiments have nominal carboxyl group densities of 400 μmol/g. Interestingly, a sample of carboxylated polystyrene particles 6.39 μm in diameter (microParticles GmbH) with carboxyl group densities of 15 μmol/g did not display cluster formation behaviour in our experiments. Eq. S 7 clearly shows that the magnitude of the interfacial free energy contribution is proportional to the density of ionizable groups in the particle (Γ), providing a conceivable explanation for the absence of visible cluster formation in particle preparations carrying a low density of ionizable surface groups.

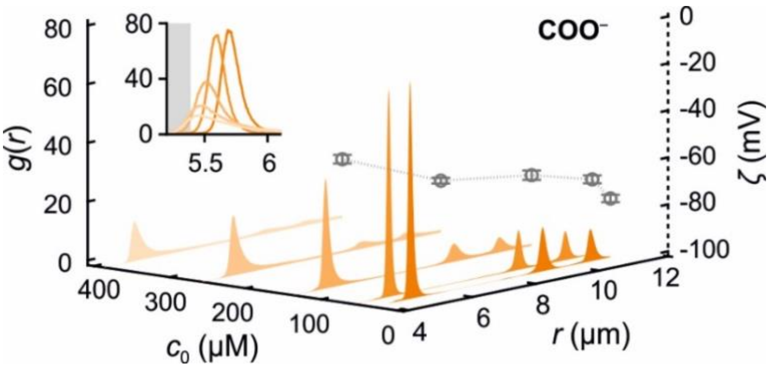

Fig. S15. Ionic strength dependence of the interactions of COOH particles in aqueous electrolyte at p*H*=5.8. The overall trend recapitulates the observations for SiO_2 particles. ζ potential measurements represent mean values ± S.D from 3 sets of measurements.

S4.6 pH dependence of the interactions of PEI-coated silica particles

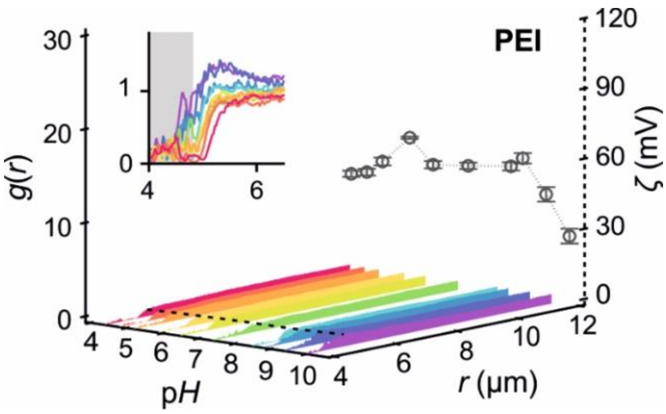

Fig. S16. p*H* dependence of the interactions of PEI-coated silica particles in aqueous electrolyte at constant salt concentration, c_0 ≈ 0.12mM NaCl. Negatively charged silica particles coated with PEI polymer (via the

method outlined in Section S1.3) become positively charged over a broad pH spectrum, as reflected in the zeta (ζ) potential measured across this range. In accordance with the results shown in Fig. 3 for poly-K and PDADMAC polymers, no clustering is observed for these positively charged (coated) particles in water. Matching of the experimental radial probability distribution functions $g(r)$ with those from BD simulations for a polyelectrolyte coating experiment involving PEI coated particles give inferred pair interaction potentials $U(x)$ that are purely repulsive (see Fig. S23). ζ potential measurements represent mean values ± S.D from 3 sets of measurements

S4.7 Ionic-strength dependent swelling of positively charged polyelectrolyte coatings detected in *g*(*r*) measurements

Although the $g(r)$ profile for a system of repulsive particles is relatively featureless, for experiments involving positively charged poly-K and PDADMAC coated particles we observe a markedly different profile for the radial probability distribution functions $g(r)$s when compared to positively charged NH_2-SiO_2 particles (see Fig. S17 and Fig. S23). We found that the slope of the $g(r)$ curve at a location around the nominal particle diameter was significantly smaller for the positively charged polyelectrolyte coated particles, gradually increasing and attaining a value of $g(r) \approx 1$ at an interparticle distance of $r \approx 8$ µm which is much larger than the corresponding value of $r \approx 5$ µm observed on experiments for NH_2-SiO_2 particles (Fig. S23). This is a significant disparity in behaviour given that the silica particles used for polymer coating experiments are only about 1 µm larger in diameter than the NH_2-SiO_2 particles. On first glance, this might suggest an exceptionally long ranged repulsion in the interparticle interaction. We noted however that varying the prefactor A and setting $B = 0$ in Eq. S 2 and varying the screening length for a purely repulsive pair interaction potential $U(x)$ could not capture the experimentally observed behaviour in a BD simulation. However, we then found that a BD simulation with a particle size distribution whose mean is larger than that of the nominal size distribution by about 1.7 µm was better able to match the experimental $g(r)$s (see Fig. S17). We suggest that the observations likely reflect a contribution to an enlarged effective particle size from swollen and highly repulsive polyelectrolyte brushes attached to the surface of the particles (Fig. S17a). PDADMAC used in the experiment has a molecular weight of 350,000 g/mol and a total contour length, $l_{\max}$, of around 2 µm. Thus, nearly fully extended mutually repulsive polymer chains at the surface could be thought to contribute to a significant increase in the effective particle diameter (Fig. S17).

To further investigate this phenomenon and to test our hypothesis, PDADMAC coated particles were suspended in solutions of different salt concentrations and the corresponding $g(r)$s measured (see Fig. S17). At $c_0 = 0.05$ mM NaCl, the characteristic 'long range' behaviour can be observed in the $g(r)$. The region of the $g(r)$ with gradually increasing slope extended to as far as $r = 10$ µm which cannot be explained with the nominal particle diameter of $2R = 4.98$ µm and the relevant Debye length. We then found that increasing the salt concentration to 0.15 mM and higher, the form of the measured $g(r)$ altered substantially (see Fig. S17), reverting to behaviour that is captured in BD simulations performed using the nominal particle size distribution. We attribute these observations to ionic strength dependent electrostatic swelling and collapse of surface polymer brushes. We observe similar behaviour for poly-K coated spheres, and we model the interaction in a similar fashion.

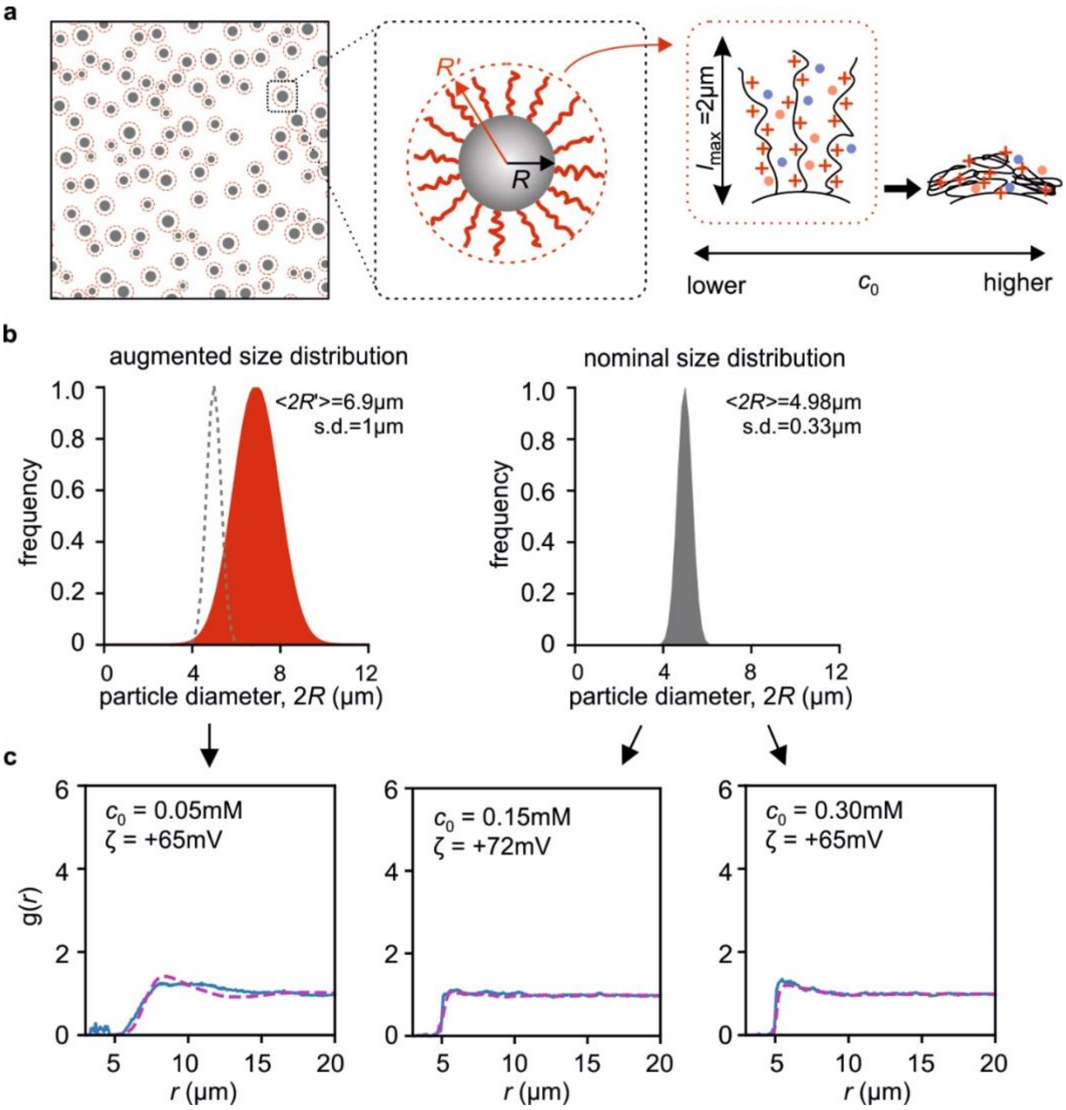

Fig. S17. Interactions of PDADMAC coated silica particles in electrolytes with different salt concentrations. (**a**) Illustration of the swelling and collapse of a positively charged polyelectrolyte brush at low salt and high salt concentrations, inferred from these measurements. Snapshot of a configuration of particles in a BD simulation (left). Silica particles are denoted as grey discs and the red halos around the particles denote an effective "swollen" diameter due to surface attached polyelectrolyte chains. (**b**) Particle size distributions used in BD simulations for polymer coated particles at low salt concentrations, $c_0 < 0.1$ mM (left) and higher salt concentrations (right). (**c**) Overlays of experimentally measured (blue) and simulated (dashed magenta) $g(r)$s for PDADMAC coated particles in solutions of different ionic strength. A particle size distribution with an increased average diameter of $2(R + l_{\max})$ was needed to better capture experiments at lower salt concentrations.

S5 Data Processing & Analysis

S5.1 Matching experimentally measured $g(r)$s with those from BD simulations

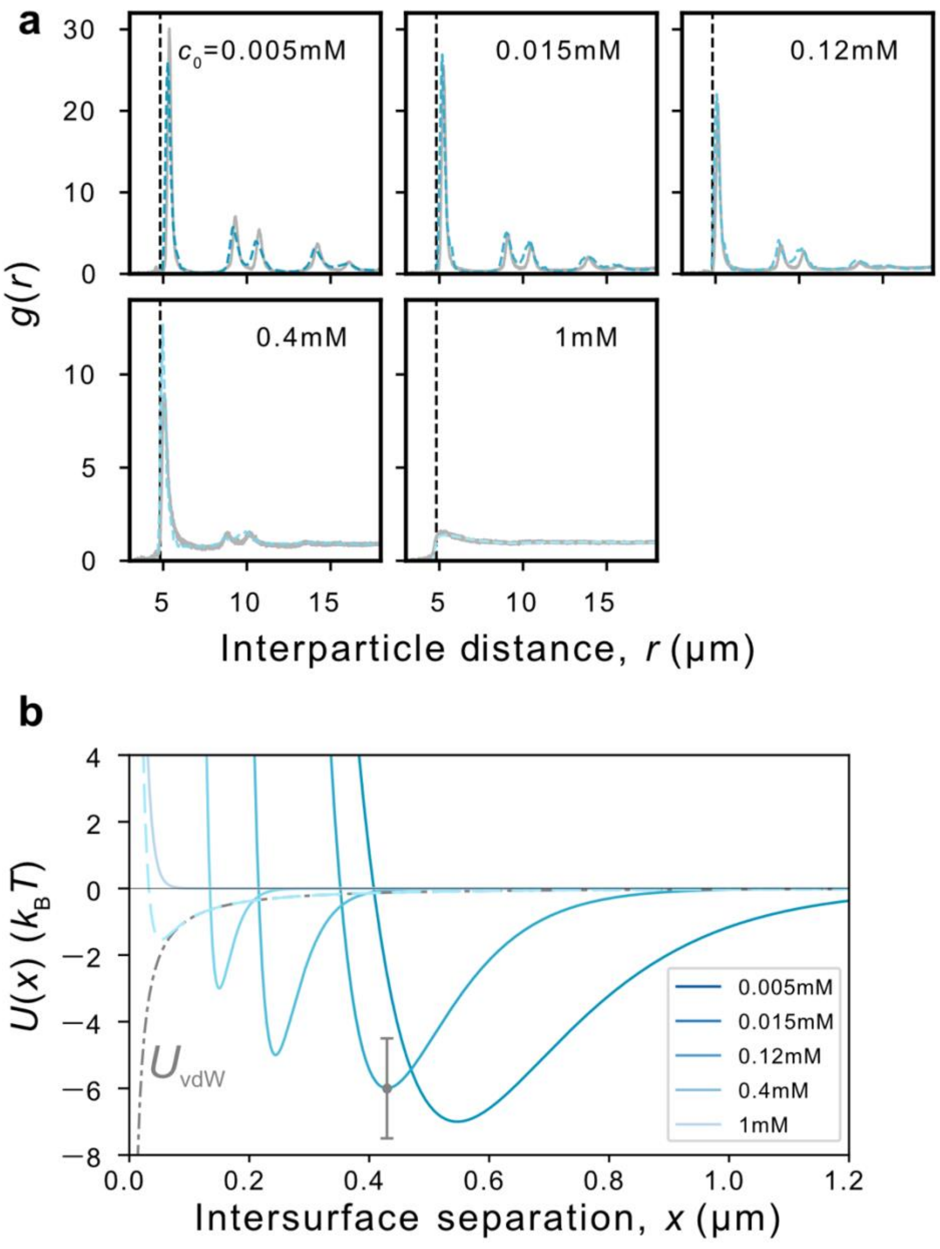

Fig. S18. Matching of the experimental radial probability distribution functions $g(r)$s with those from BD simulations for experiments that vary the salt concentration c_0 for SiO_2 particles in water (shown in Fig. 1). (**a**) The experimental and simulated data (grey and dashed shaded blue lines respectively) are presented overlaid. The black dashed vertical line indicates the nominal particle diameter. (**b**) Corresponding inferred pair interaction potentials $U(x)$. The grey dashed line shows the vdW contribution, which is discussed in detail in Section S2.1. The vertical error bar denotes an estimated uncertainty of $\pm 1.5\ k_BT$ in the overall process, arising from particle size uncertainties and drift in experimental conditions over time that affect cluster properties. The same set of curves presented in the main text figure includes a horizontal error bar on the intersurface separation x. This error bar captures the overall uncertainty in measured interparticle separation (arising from the particle size dispersion and localisation error, see Section S1.6), and is intended to facilitate comparison with a calculated theoretical pair potential where the particle diameter is fixed.

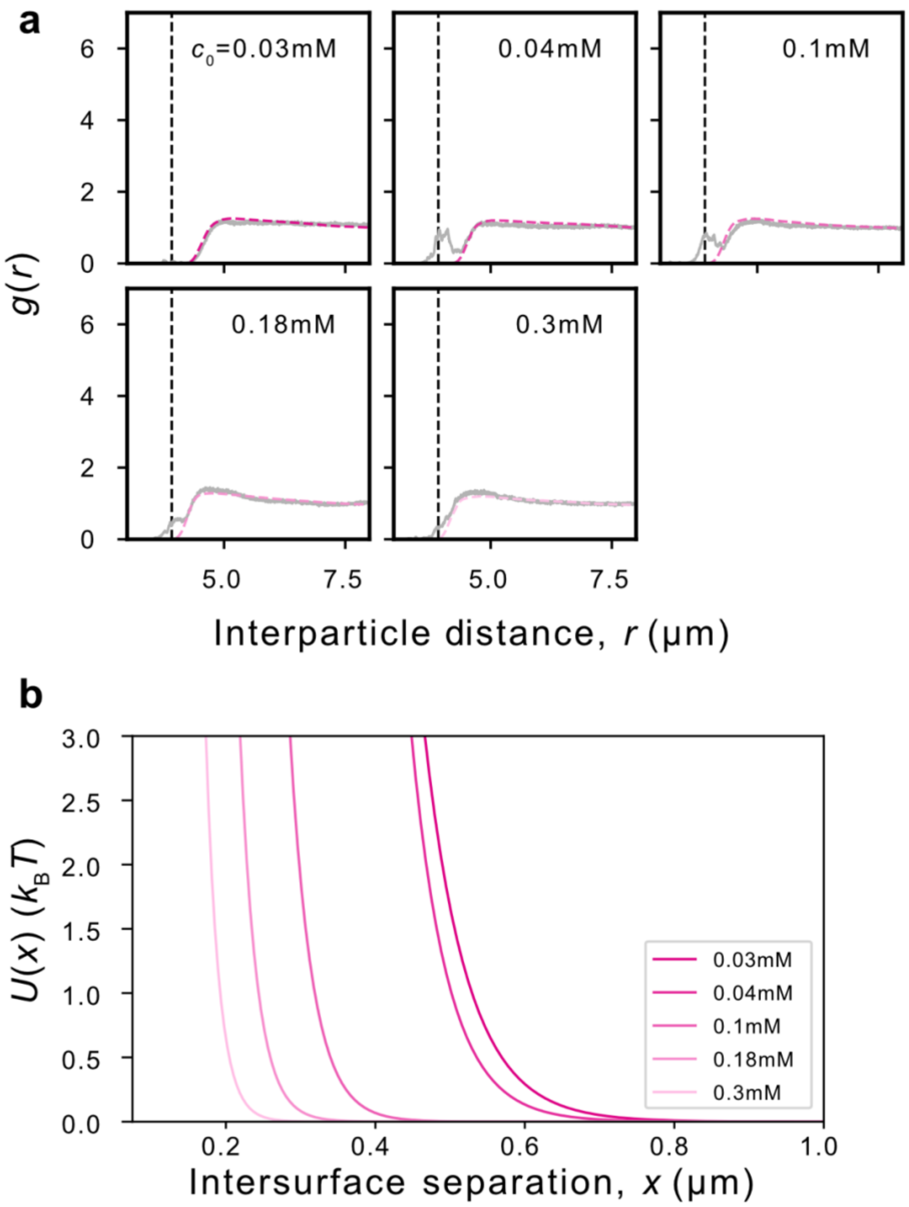

Fig. S19. Matching the experimental radial probability distribution functions $g(r)$s with those from BD simulations for experiments varying the salt concentration for NH2-SiO_2 particles in water (shown in Fig. 1). (**a**) Overlays of experimental and simulated data (grey and dashed shaded pink lines respectively). The black dashed vertical line indicates the nominal particle diameter. (**b**) Corresponding inferred pair interaction potentials $U(x)$. The same set of curves presented in the main text figure includes a horizontal error bar on the intersurface separation x. This error bar captures the overall uncertainty in measured interparticle separation (arising from the particle size dispersion and localisation error, see Section S1.6), and is intended to facilitate comparison with a calculated theoretical pair potential where the particle diameter is fixed.

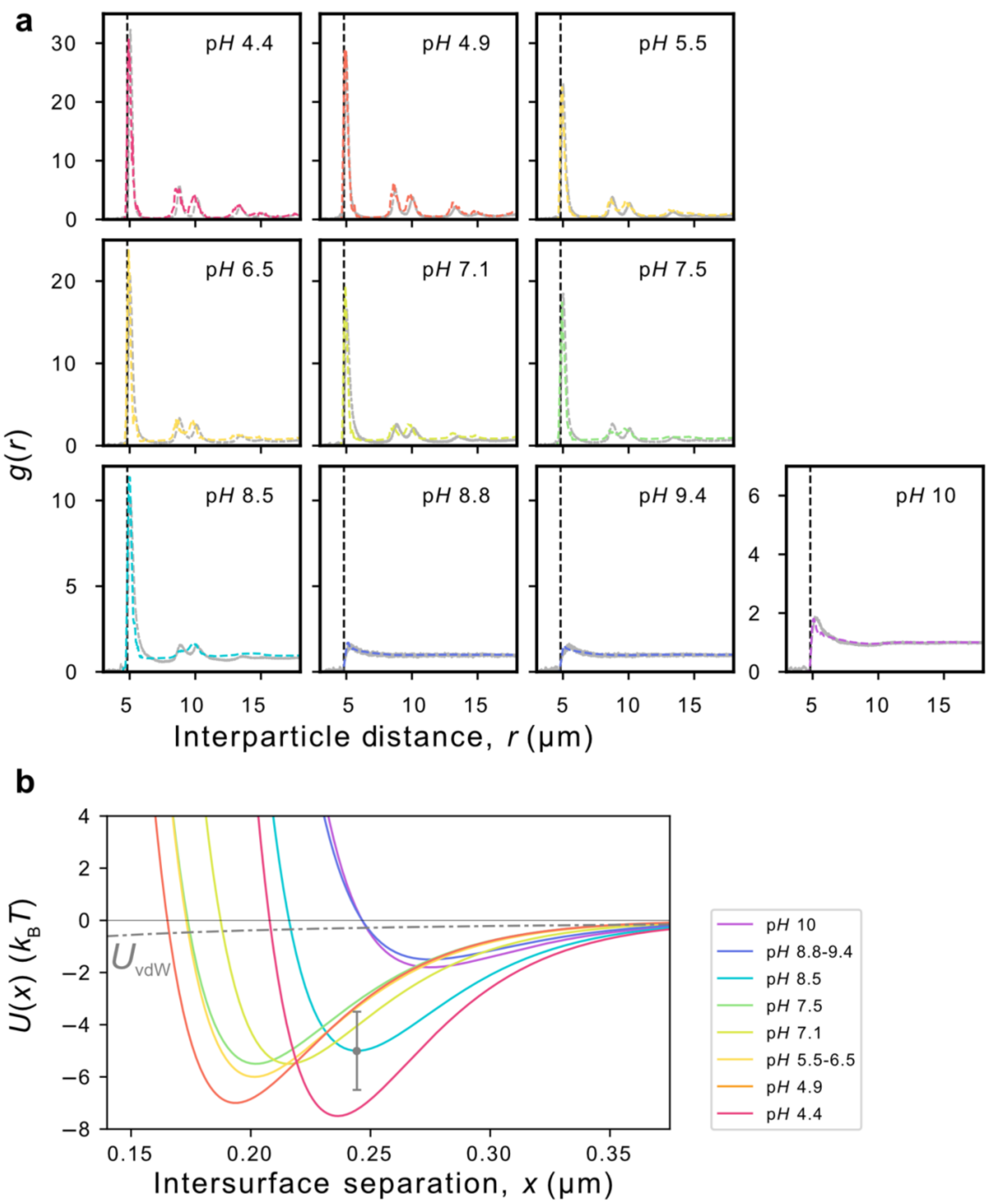

Fig. S20. Matching the experimental radial probability distribution functions $g(r)$s with those from BD simulations for experiments varying the p*H* for SiO_2 particles in water (shown in Fig. 2). (**a**) Overlays of experimental and simulated data (grey and dashed coloured lines respectively). The black dashed vertical line indicates the nominal particle diameter. (**b**) Corresponding inferred pair interaction potentials $U(x)$. The vertical error bar denotes an estimated uncertainty of $\pm 1.5\ k_{\mathrm{B}}T$ in the overall process, arising from particle size uncertainties and drift in experimental conditions over time that affect cluster properties. The same set of curves presented in the main text figure includes a horizontal error bar on the intersurface separation x. The error bar captures the overall uncertainty in measured interparticle separation (arising from the particle size dispersion and localisation error, see Section S1.6), and is intended to facilitate comparison with a calculated theoretical pair potential where the particle diameter is fixed.

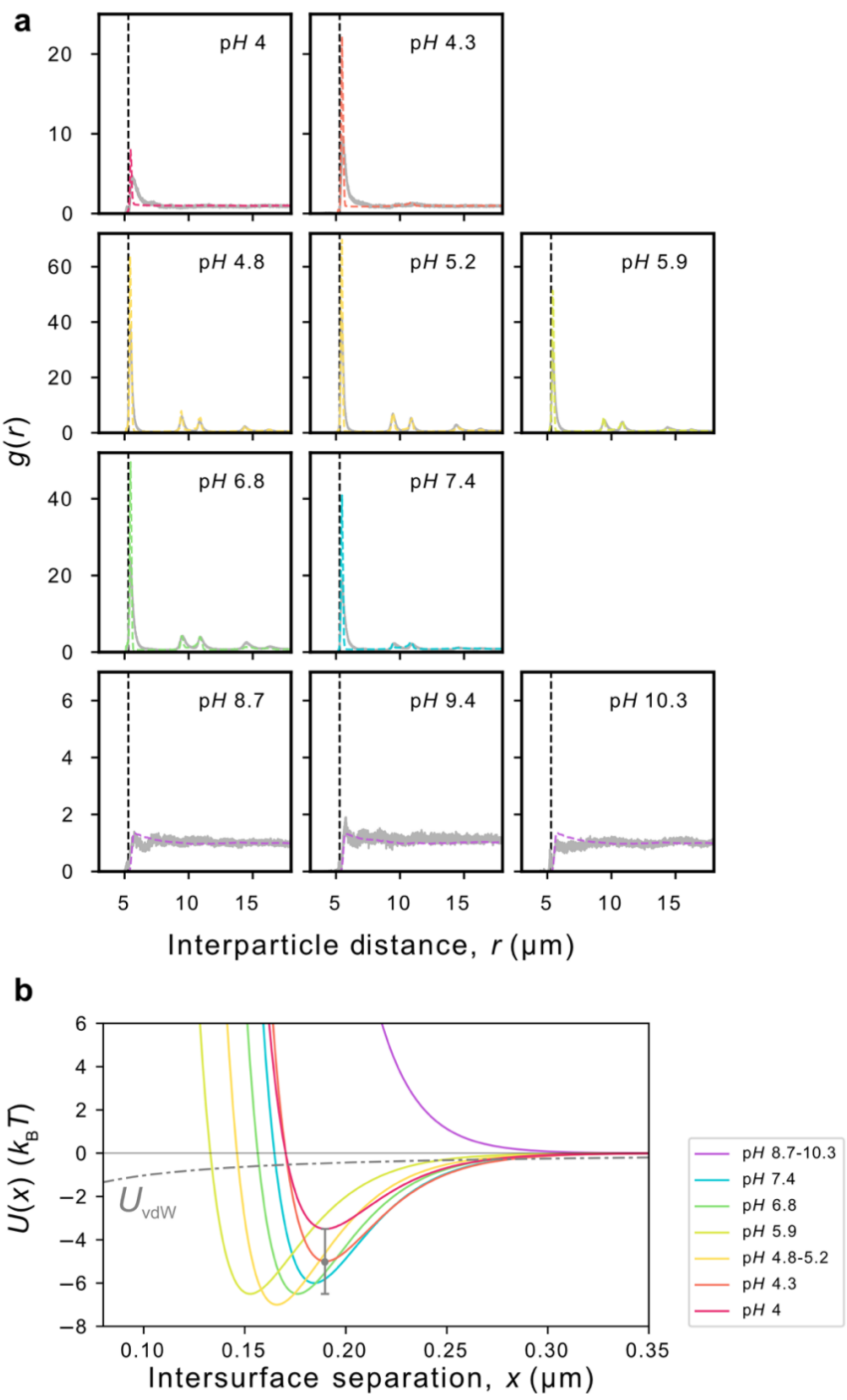

Fig. S21. Matching the experimental radial probability distribution functions $g(r)$s with those from BD simulations for experiments varying the pH for COOH particles in water (shown in Fig. 2). (**a**) Overlays of experimental and simulated data (grey and dashed coloured lines respectively). The black dashed vertical line indicates the nominal particle diameter. (**b**) Corresponding inferred pair interaction potentials $U(x)$. The vertical error bar denotes an estimated uncertainty of $\pm 1.5\ k_{\mathrm{B}}T$ in the overall process, arising from particle size uncertainties and drift in experimental conditions over time that affect cluster properties. The same set of curves presented in the main text figure includes a horizontal error bar on the intersurface separation x. This error bar captures the overall uncertainty in measured interparticle separation (arising from the particle size dispersion and localisation error, see Section S1.6), and is intended to facilitate comparison with a calculated theoretical pair potential where the particle diameter is fixed.

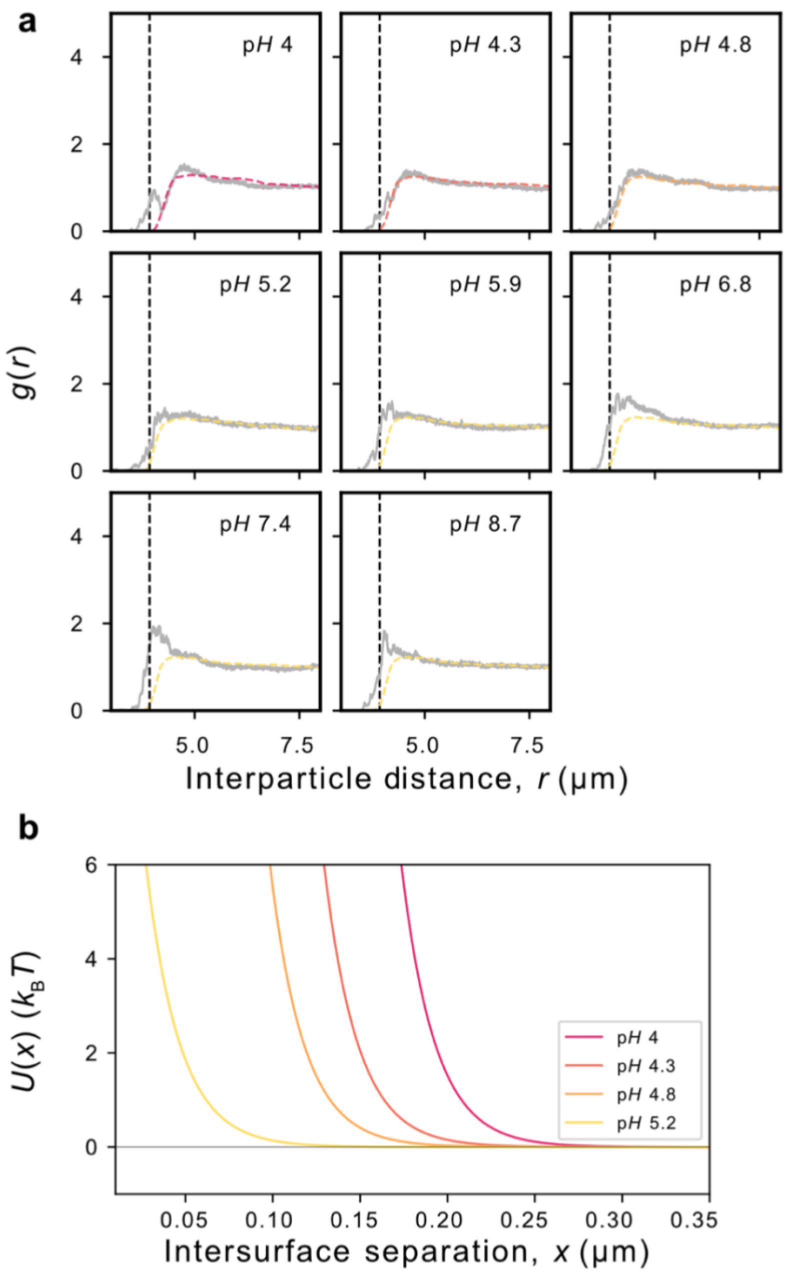

Fig. S22. Matching the experimental radial probability distribution functions *g*(*r*)s with those from BD simulations experiments varying the p*H* for NH2-SiO_2 particles in water (shown in Fig. 2). (**a**) Overlays of experimental and simulated data (grey and dashed coloured lines respectively). The black dashed vertical line indicates the nominal particle diameter. (**b**) Corresponding inferred pair interaction potentials $U(x)$. Experimental data for p$H \geq 5.9$ reveals an increasing proportion of particles stuck particles, presumably in vdW contact with one another. This manifests in a peak in the $g(r)$ plot around the nominal particle diameter. $U(x)$ parameters for these cases cannot be reliably extracted. The same set of curves presented in the main text figure includes a horizontal error bar on the intersurface separation x. This error bar captures the overall uncertainty in measured interparticle separation (arising from the particle size dispersion and localisation error, see Section S1.6), and is intended to facilitate comparison with a calculated theoretical pair potential where the particle diameter is fixed.

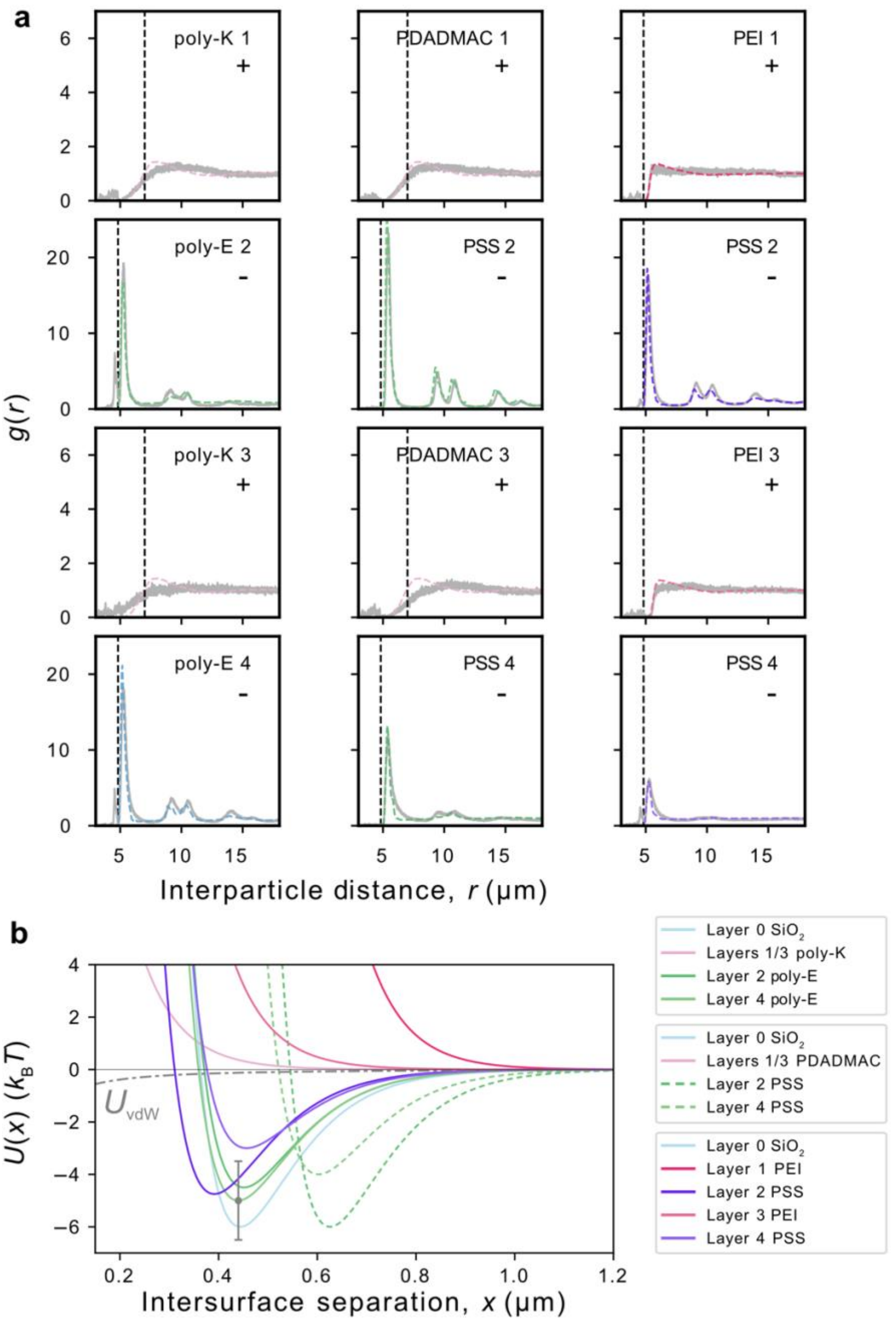

Fig. S23. Matching the experimental radial probability distribution functions $g(r)$s with those from BD simulations for the polyelectrolyte coating experiments (shown in Fig. 3). (**a**) Overlays of experimental and simulated data (grey and dashed coloured lines respectively). The plot inset identifies the polymer coating type in the experiment and the charge of the polymer coating. Each individual experiment is presented column-wise, proceeding from to bottom. Black dashed vertical line indicates the nominal particle diameter. Note that a particle size distribution with an augmented particle diameter was used to fit the data corresponding to layers 1 and 3 of particles coated with poly-K and PDADMAC, as discussed further in Section S4.7. (**b**) Corresponding inferred pair interaction potentials $U(x)$. The vertical error bar denotes an estimated uncertainty of $\pm 1.5\ k_{\mathrm{B}}T$ in the overall process, arising from particle size uncertainties and drift in experimental conditions over time that affect cluster properties. The same set of curves presented in the main text figure includes a horizontal error bar on the intersurface separation x. This error bar captures the overall uncertainty in measured interparticle separation (arising from the particle size dispersion and localisation error, see Section S1.6), and is intended to facilitate comparison with a calculated theoretical pair potential where the particle diameter is fixed.

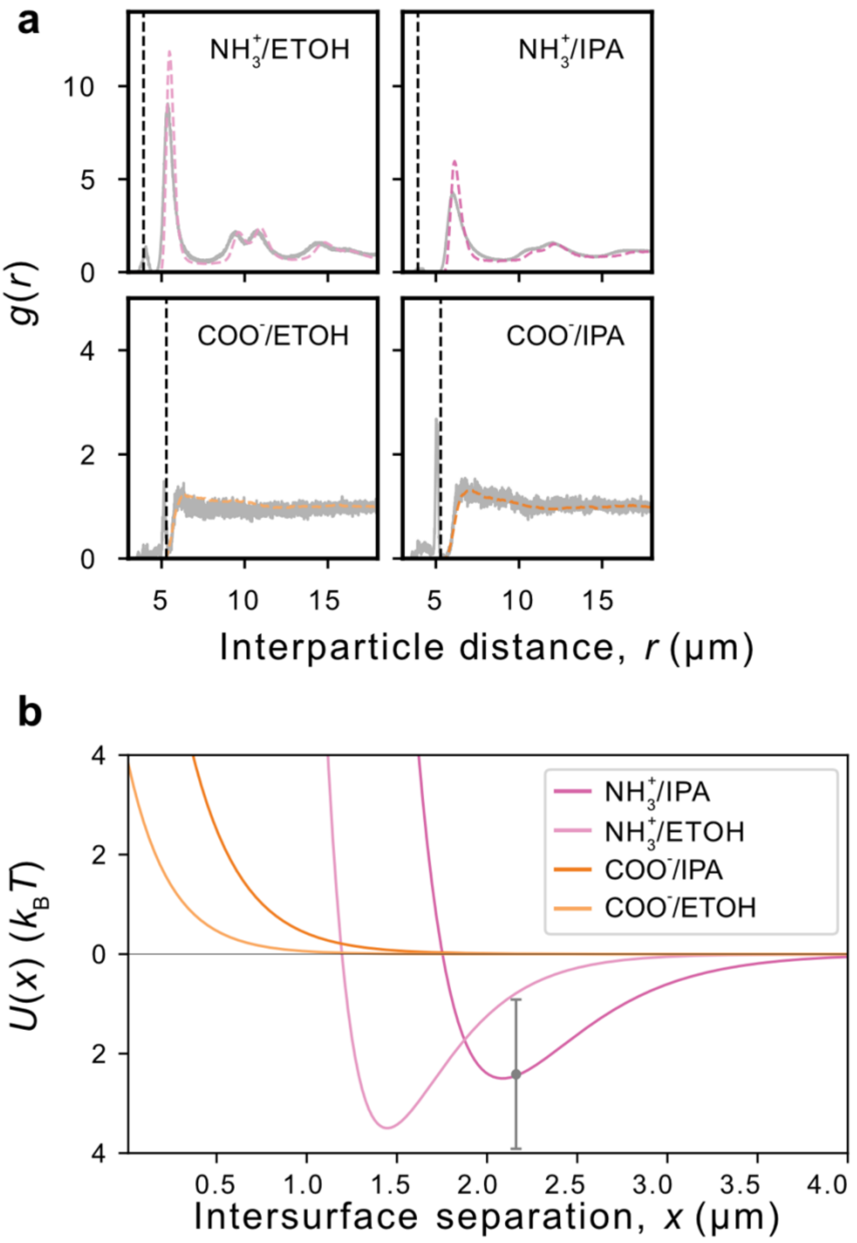

Fig. S24. Matching the experimental radial probability distribution functions $g(r)$s with those from BD simulations for experiments in alcohols (shown in Fig. 5c). (**a**) Overlays of experimental and simulated data (grey and dashed coloured lines respectively). The plot inset the identifies particle type and the solvent in the experiment. The black dashed vertical line indicates the nominal particle diameter. (**b**) Corresponding inferred pair interaction potentials $U(x)$. The vertical error bar denotes an estimated uncertainty of $\pm 1.5\ k_B T$ in the overall process, arising from particle size uncertainties and drift in experimental conditions over time that affect cluster properties. The same set of curves presented in the main text figure includes a horizontal error bar on the intersurface separation x. This error bar captures the overall uncertainty in measured interparticle separation (arising from the particle size dispersion and localisation error, see Section S1.6), and is intended to facilitate comparison with a calculated theoretical pair potential where the particle diameter is fixed.

S5.2 Overview of all experimental snapshots and corresponding digitized images

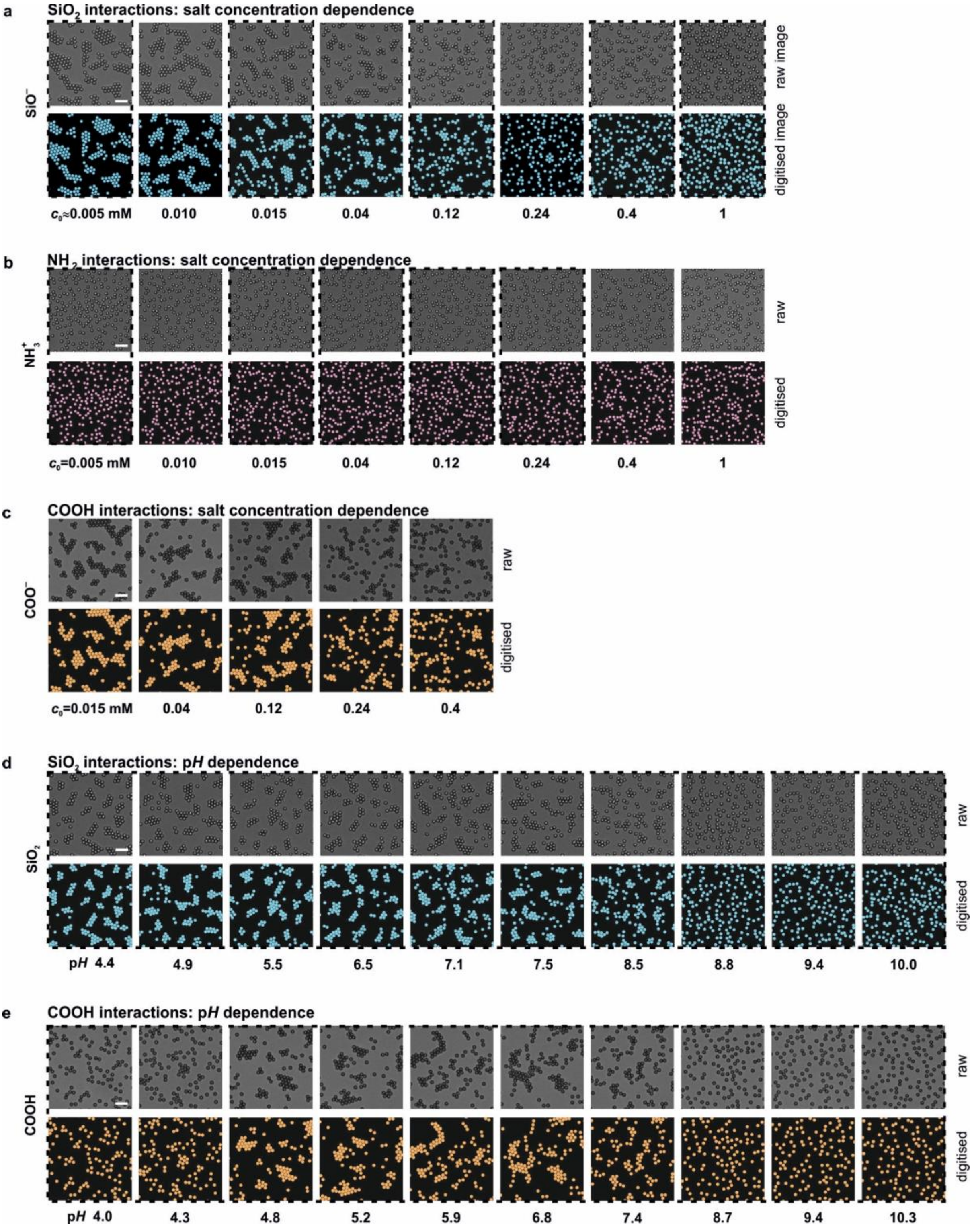

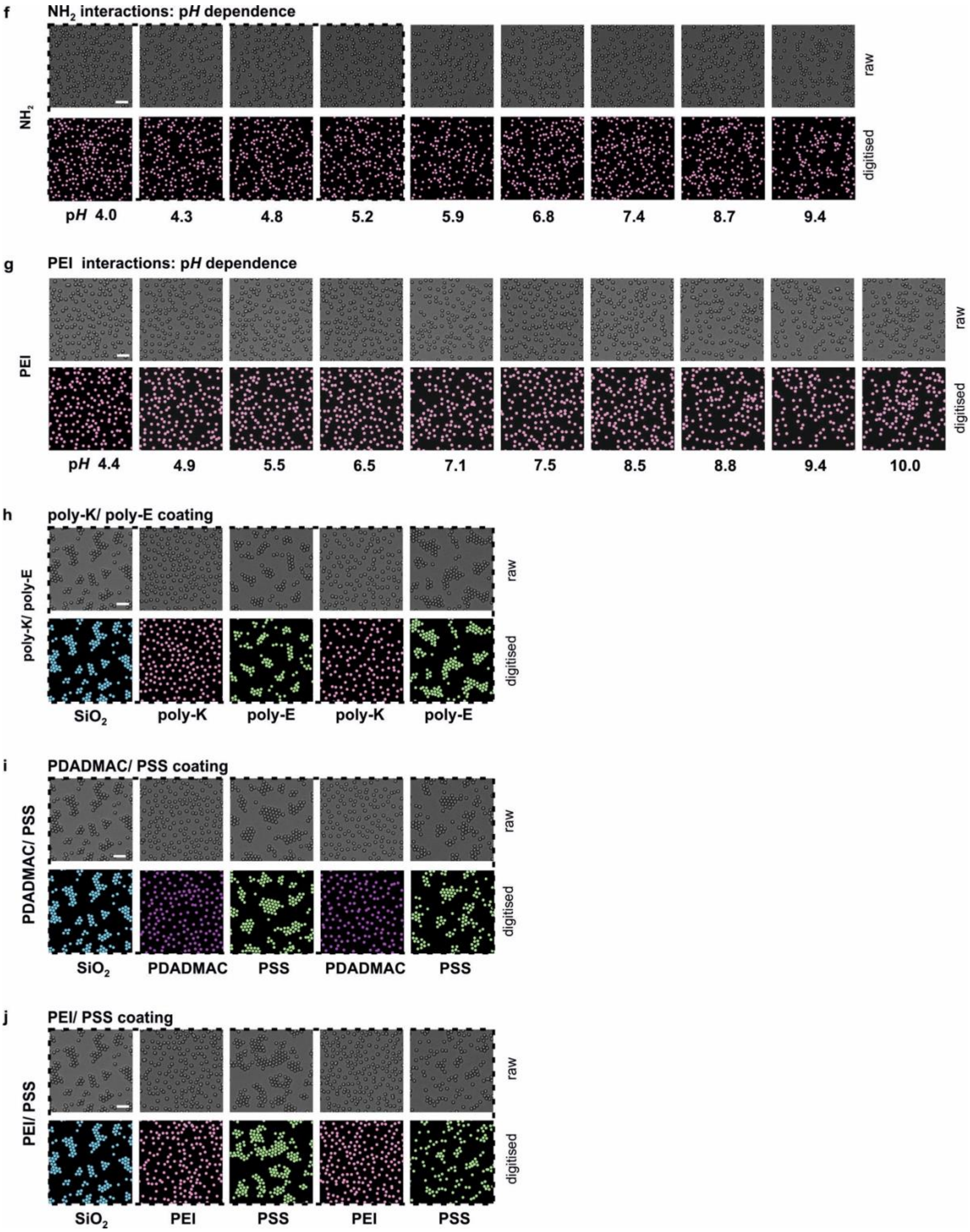

f
NH_2 interactions: pH dependence
NH_2
raw
digitised
pH 4.0
4.3
4.8
5.2
5.9
6.8
7.4
8.7
9.4
g
PEI interactions: pH dependence
PEI
raw
digitised
pH 4.4
4.9
5.5
6.5
7.1
7.5
8.5
8.8
9.4
10.0
h
poly-K/ poly-E coating
poly-K/ poly-E
raw
digitised
SiO_2
poly-K
poly-E
poly-K
poly-E
i
PDADMAC/ PSS coating
PDADMAC/ PSS
raw
digitised
SiO_2
PDADMAC
PSS
PDADMAC
PSS
j
PEI/ PSS coating
PEI/ PSS
raw
digitised
SiO_2
PEI
PSS
PEI
PSS

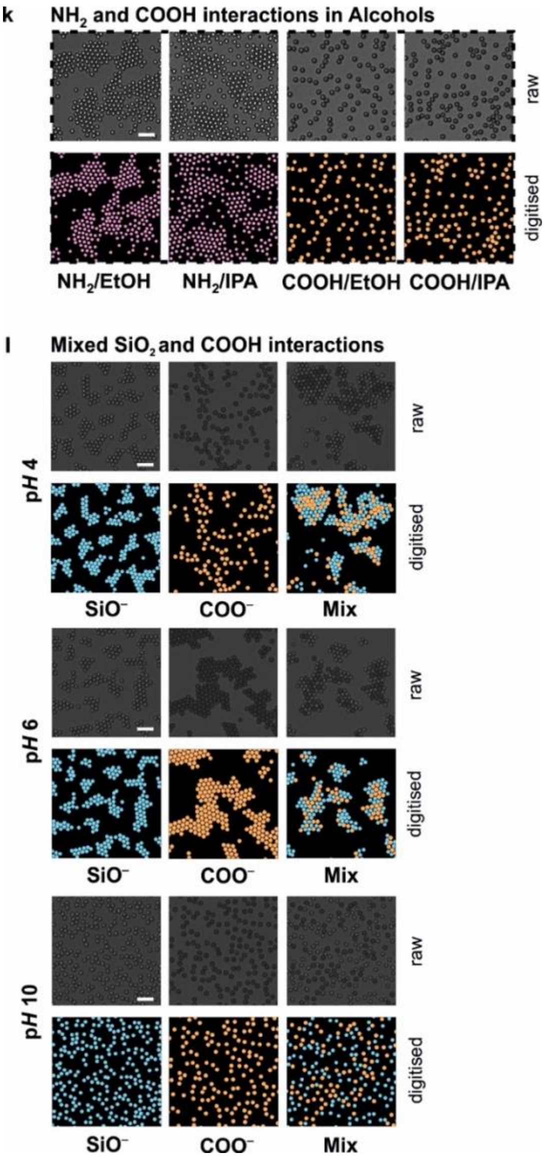

Fig. S25. Overview of all experimental snapshots and corresponding digitized images. Highlighted experiments (black dashed line) are cases accompanied by BD simulation results. Scale bar 20 µm.

S6 Supplementary Tables

Table S1. Parameter values used in PB-interfacial solvation model calculations of $U_{tot}(x)$

Calculation parameters							Experimental parameters		
**FIG. #**	**Particle**	**Diameter, $2R$ (μm)**	**Solvent (ε: dielectric constant)**	**c_0 (mM)**	**$p = \mathrm{p}K - \mathrm{p}H$**	**Γ (nm^{-2})**	**c_0 (mM)**	**pH**	**nominal pK**
1 (d)	SiO_2	4.82	H_2O ($\varepsilon = 78.5$)	0.005	0.030	1	0.005	6.4	2-11
			″	0.016	-0.525		0.015	5.5	″
			″	0.12	-0.950		0.12	5.4	″
			″	1.012	-1.500		1	5.4	″
	NH_2	3.92	″	0.04	3.500	0.2	0.005	6.4	9.5
			″	0.1	″		0.04	5.6	″
			″	0.2	″		0.12	5.6	″
			″	0.3	″		0.24	5.4	″
2 (c)	COOH	5.29	″	0.25	1.000 ○	0.1	0.25	4	4.5
			″	″	0.500 ○		0.26	4.3	″
			″	″	-0.500 ○		0.25	5.2	″
			″	″	-1.500 ○		0.25	5.9	″
			″	″	-2.500 ○		0.25	7.4	″
			″	″	-5.000 ○		0.25	10.3	″
	NH_2	3.92	″	0.31	5.000 ○	0.2	0.25	4	9.5
			″	0.18	″ □		0.25	4	″
5 (d)	NH_2	3.92	IPA ($\varepsilon = 20$)	0.075	-0.265	0.2	0.0002	8.1	< 7
	COOH	5.29	EtOH ($\varepsilon = 20$)	0.0004	3.500	0.1	0.0004	7.3	≳ 10
			IPA ($\varepsilon = 20$)	0.0002	4.500		0.0003	8.1	″

* additional parameters used in SiO_2 calculations: p_1=-5, f_1=0.005 (see Section S3.3)
** coloured symbols refer to calculated curves in Fig. 2c

Table S2. BD simulation parameter values used to model experiments that examine the salt concentration dependence of cluster formation for SiO_2 particles in water

			Simulation parameters						Expt. Parameters		
**Particle**	$2R$ (μm)	**Solvent**	w (k_BT)	A (k_BT)	B (k_BT)	κ_1 (μm^{-1})	κ_2 (μm^{-1})	A_H (k_BT)	c_0 (mM)	pH	ζ (mV)
			7	7433.859	-6399.154	7.355	6.988	-	0.005	6.4	-40
			6	32580.3	-25848.1	13.158	12.5	-	0.015	5.5	-50
**SiO_2**	4.82	H_2O	5	623224	-422741	36.0344	34.2327	-	0.12	5.4	-42
			3	1073860	-690988	65.6248	62.3436	-	0.4	5.4	-40
			0	100	0	104.645	-	0.6	1	5.4	-26

Values for parameters in experiments are presented on the right for convenience.

Table S3. BD simulation parameter values used to model experiments that examine the salt concentration dependence of interparticle interactions for NH2-SiO_2 particles in water

			Simulation parameters							Expt. parameters		
**Particle**	**$2R$ (μm)**	**Solvent**	**c_0 (mM)***	**w (k_BT)**	**A (k_BT)**	**B (k_BT)**	**κ_1 (μm^{-1})**	**κ_2 (μm^{-1})**	**A_H (k_BT)**	**c_0 (mM)**	**pH**	**ζ (mV)**
**NH_2-SiO_2**	3.92	H_2O	0.03	0	10000	0	17.4	-	-	0.005	6.4	+57
			0.04	〃	30000	〃	20.5427	-	-	0.015	5.5	+65
			0.10	〃	40000	〃	33.222	-	-	0.04	5.6	+84
			0.18	〃	50000	〃	44.377	-	-	0.12	5.4	+84
			0.30	〃	60000	〃	57.165	-	-	0.24	5.4	+57

* denotes values that are slightly different from the corresponding experimentally measured values as discussed in the text, see Section S3.3.

Table S4. BD simulation parameter values used to model experiments that examine the pH dependence of cluster formation for SiO_2 particles in water

			Simulation parameters						Expt. parameters		
Particle	$2R$ (μm)	Solvent	w (k_BT)	A (k_BT)	B (k_BT)	κ_1 (μm^{-1})	κ_2 (μm^{-1})	A_H (k_BT)	c_0 (mM)	pH	ζ (mV)
**SiO_2**	4.82	H_2O	1.75	688093	-441309	36.0344	34.2327	-	0.12	10	-110
			1.5	573411	-367757	″	″	-	0.11	9.4	-100
			1.5	573411	-367757	″	″	-	0.11	8.8	-99
			5	635884	-430895	″	″	-	0.12	8.5	-96
			5.5	152267	-111354	″	″	-	0.11	7.5	-83
			5.5	249525	-178029	″	″	-	0.11	7.1	-80
			6	162493	-118964	″	″	-	0.12	6.5	-80
			6	162493	-118964	″	″	-	0.11	5.5	-69
			7	142646	-105929	″	″	-	0.11	4.9	-50
			7.5	711699	-489383	″	″	-	0.12	4.4	-23

Table S5. Alternative set of parameters for the experiments varying pH for SiO_2 particles in water

			**Simulation parameters**						**Expt. parameters**		
**Particle**	$2R$ (µm)	**Solvent**	w (k_BT)	A (k_BT)	B (k_BT)	κ_1 (µm^{-1})	κ_2 (µm^{-1})	A_H (k_BT)	c_0 (mM)	pH	ζ (mV)
**SiO_2**	4.82	H_2O	1.5	688093	-441309	36.0344	34.2327	-	0.12	10	-110
			1.75	573411	-367757	″	34.2327	-	0.11	9.4	-100
			1.75	573411	-367757	″	34.2327	-	0.11	8.8	-99
			5	444640	-258066	″	33.512	-	0.12	8.5	-96
			5.5	392688	-346379	″	35.314	-	0.11	7.5	-83
			5.5	249525	-178029	″	34.2327	-	0.11	7.1	-80
			6	162493	-118964	″	34.2327	-	0.12	6.5	-80
			6	162493	-118964	″	34.2327	-	0.11	5.5	-69
			7	142646	-105929	″	34.2327	-	0.11	4.9	-50
			7.5	118616	-20216	″	27.386	-	0.12	4.4	-23

The parameters capture the increase of A increasing with increasing pH, retaining the same functional form of the pair interaction potentials in Table S4. Here, the value of κ_2 is adjusted to achieve a similar $U(x)$ profile.

Table S6. BD simulation parameter values used to model experiments that examine the pH dependence of cluster formation for COOH particles in water

			Simulation parameters						Expt. parameters		
Particle	$2R$ (μm)	Solvent	w ($k_B T$)	A ($k_B T$)	B ($k_B T$)	κ_1 (μm^{-1})	κ_2 (μm^{-1})	A_H ($k_B T$)	c_0 (mM)	pH	ζ (mV)
**COOH**	5.29	H_2O	0	500000	0	52.0111	49.4106	-	0.25	10.3	-67
			0	500000	0	〃	〃	-	0.24	9.4	-67
			0	500000	0	〃	〃	-	0.24	8.7	-81
			6	1667860	-1086860	〃	〃	-	0.25	7.4	-84
			6.5	1177800	-784111	〃	〃	-	0.25	6.8	-86
			6.5	347454	-245874	〃	〃	-	0.25	5.9	-81
			7	743406	-508316	〃	〃	-	0.25	5.2	-78
			7	743406	-508316	〃	〃	-	0.25	4.8	-65
			5	1859830	-1194430	〃	〃	-	0.26	4.3	-33
			3.5	1301880	-836103	〃	〃	-	0.25	4.0	-18

Table S7. Alternative set of parameters for the experiments varying the pH for COOH particles in water

			Simulation parameters						Expt. parameters		
**Particle**	$2R$ (μm)	**Solvent**	w (k_BT)	A (k_BT)	B (k_BT)	κ_1 (μm^{-1})	κ_2 (μm^{-1})	A_H (k_BT)	c_0 (mM)	pH	ζ (mV)
**COOH**	5.29	H_2O	0	500000	0	52.0111	49.4106	-	0.25	10.3	-67
			0	500000	0	〃	49.4106	-	0.24	9.4	-67
			0	500000	0	〃	49.4106	-	0.24	8.7	-81
			6	427943	-101001	〃	43.169	-	0.25	7.4	-84
			6.5	380792	-122764	〃	44.7	-	0.25	6.8	-86
			6.5	347454	-245874	〃	49.4106	-	0.25	5.9	-81
			7	261545	-97934	〃	45.25	-	0.25	5.2	-78
			7	261545	-97934	〃	45.25	-	0.25	4.8	-65
			5	201716	-9417	〃	33.8	-	0.26	4.3	-33
			3.5	141201	-6592	〃	33.8	-	0.25	4	-18

The parameters capture the increase of A increasing with increasing pH, retaining the same functional form of the pair interaction potentials in Table S6. Here, the value of κ_2 is adjusted to achieve a similar $U(x)$ profile.

Table S8. BD simulation parameter values used to model experiments that examine the p*H* dependence of interparticle interactions for NH2-SiO_2 particles in water

			Simulation parameters						Expt. parameters		
Particle	$2R$ (μm)	Solvent	w (k_BT)	A (k_BT)	B (k_BT)	κ_1 (μm^{-1})	κ_2 (μm^{-1})	A_H (k_BT)	c_0 (mM)	pH	ζ (mV)
**NH_2**	3.92	H_2O	0	25	0	52.0111	-	-	0.25	5.2	+92
			〃	1000	〃	〃	-	-	0.25	4.8	+93
			〃	5000	〃	〃	-	-	0.26	4.3	+90
			〃	50000	〃	〃	-	-	0.25	4	+94

Table S9. BD simulation parameter values used to model experiments that examine the dependence of cluster formation on the charge of the polypeptide coating layer (poly-K/poly-E) displayed in Fig. 3c

			Simulation parameters						Expt. parameters		
**Particle**	$2R$ (μm)	**Solvent**	w (k_BT)	A (k_BT)	B (k_BT)	κ_1 (μm^{-1})	κ_2 (μm^{-1})	A_H (k_BT)	c_0 (mM)	pH	ζ (mV)
**SiO_2**	4.82	H_2O	6	32580	-25848	12.7401	12.1031	-	0.02	5.5	-50
**poly-K**	*6.5		0	100	0	〃	〃	-	〃	〃	+56
**poly-E**	4.82		4.5	26845	-21198	〃	〃	-	〃	〃	-39
**poly-K**	*6.5		0	100	0	〃	〃	-	〃	〃	+52
**poly-E**	4.82		5	25759	-20490	〃	〃	-	〃	〃	-31

* augmented average particle diameter. See Section S4.7 for details.

Table S10. BD simulation parameter values used to model experiments that examine the dependence of cluster formation on the charge of the polyelectrolyte coating layer (PDADMAC/PSS) displayed in Fig. 3c

Particle	$2R$ (μm)	Solvent	Simulation parameters w (k_BT)	A (k_BT)	B (k_BT)	κ_1 (μm^{-1})	κ_2 (μm^{-1})	A_H (k_BT)	Expt. parameters c_0 (mM)	pH	ζ (mV)
**SiO_2**	4.82	H_2O	6	32580	-25848	12.7401	12.1031	-	0.02	5.5	-50
**PDADMAC**	*6.5		0	100	0	〃	〃	-	〃	〃	+39
**PSS**	4.82		6	329989	-233183	〃	〃	-	〃	〃	-67
**PDADMAC**	*6.5		0	100	0	〃	〃	-	〃	〃	+62
**PSS**	4.82		4	163116	-117001	〃	〃	-	〃	〃	-62

* augmented average particle diameter. See Section S4.7 for details.

Table S11. BD simulation parameter values used to model experiments that examine the dependence of cluster formation on the charge of the polyelectrolyte coating layer (PEI/PSS)

			Simulation parameters						Expt. parameters		
**Particle**	$2R$ (µm)	**Solvent**	w (k_BT)	A (k_BT)	B (k_BT)	κ_1 (µm^{-1})	κ_2 (µm^{-1})	A_H (k_BT)	c_0 (mM)	pH	ζ (mV)
**SiO_2**	4.82	H_2O	6	32580	-25848	12.7401	12.1031	-	0.02	5.5	-50
**PEI**	4.82		0	1000	0	〃	〃	-	〃	〃	+71
**PSS**	4.82		4.75	13201	-10830	〃	〃	-	〃	〃	-37
**PEI**	4.82		0	35000	0	〃	〃	-	〃	〃	+43
**PSS**	4.82		3	19199	-15107	〃	〃	-	〃	〃	-78

Data not presented in main manuscript.

Table S12. BD simulation parameter values used to model experiments that examine the dependence of cluster formation on the sign of the charge of the particle in ethanol and isopropanol

			Simulation parameters						Expt. parameters		
Particle	$2R$ (μm)	Solvent	w (k_BT)	A (k_BT)	B (k_BT)	κ_1 (μm^{-1})	κ_2 (μm^{-1})	A_H (k_BT)	c_0 (mM)	pH	ζ (mV)
NH_2	3.92	ethanol	3.5	24179.2	-18953.4	4.0766	3.8728	-	0.0004	7.3	+66
		isopropanol	2.5	29115.9	-22234.9	3.077	2.923	-	0.0002	8.1	+55
COOH	5.29	ethanol	0	4	0	4.3	-	-	0.0004	7.3	-49
		isopropanol	0	15	0	3.6	-	-	0.0003	8.1	-22

S7 References

1 Kokot, G., Bespalova, M. I. & Krishnan, M. Measured electrical charge of SiO2 in polar and nonpolar media. *Journal of Chemical Physics* **145** (2016).

2 Masterton, W. L., Bolocofsky, D. & Lee, T. P. Ionic radii from scaled particle theory of the salt effect. *The Journal of Physical Chemistry* **75**, 2809-2815 (1971).

3 Parthasarathy, R. Rapid, accurate particle tracking by calculation of radial symmetry centers. *Nature Methods* **9**, 724-726 (2012).

4 Stein, S. C. & Thiart, J. TrackNTrace: A simple and extendable open-source framework for developing single-molecule localization and tracking algorithms. *Scientific Reports* **6**, 37947 (2016).

5 Thompson, A. P. *et al.* LAMMPS - a flexible simulation tool for particle-based materials modeling at the atomic, meso, and continuum scales. *Computer Physics Communications* **271**, 108171 (2022).

6 Kubincová, A., Hünenberger, P. H. & Krishnan, M. Interfacial solvation can explain attraction between like-charged objects in aqueous solution. *Journal of Chemical Physics* **152**, 104713 (2020).

7 Behjatian, A., Walker-Gibbons, R., Schekochihin, A. A. & Krishnan, M. Nonmonotonic Pair Potentials in the Interaction of Like-Charged Objects in Solution. *Langmuir* **38**, 786-800 (2022).

8 Hamaker, H. C. The London—van der Waals attraction between spherical particles. *Physica* **4**, 1058-1072 (1937).

9 Valmacco, V. *et al.* Dispersion forces acting between silica particles across water: influence of nanoscale roughness. *Nanoscale Horizons* **1**, 325-330 (2016).

10 Ackler, H. D., French, R. H. & Chiang, Y.-M. Comparisons of Hamaker Constants for Ceramic Systems with Intervening Vacuum or Water: From Force Laws and Physical Properties. *Journal of Colloid and Interface Science* **179**, 460-469 (1996).

11 Abraham, M. J. *et al.* GROMACS: High performance molecular simulations through multi-level parallelism from laptops to supercomputers. *SoftwareX* **1-2**, 19-25 (2015).

12 Walker-Gibbons, R., Kubincová, A., Hünenberger, P. H. & Krishnan, M. The Role of Surface Chemistry in the Orientational Behavior of Water at an Interface. *The Journal of Physical Chemistry B* **126**, 4697-4710 (2022).

13 Vanommeslaeghe, K. *et al.* CHARMM general force field: A force field for drug-like molecules compatible with the CHARMM all-atom additive biological force fields. *Journal of Computational Chemistry* **31**, 671-690 (2009).

14 Yeh, I.-C. & Wallqvist, A. On the proper calculation of electrostatic interactions in solid-supported bilayer systems. *The Journal of Chemical Physics* **134**, 055109 (2011).

15 Ninham, B. W. & Parsegian, V. A. Electrostatic potential between surfaces bearing ionizable groups in ionic equilibrium with physiologic saline solution. *Journal of Theoretical Biology* **31**, 405-428 (1971).

16 Chen, Y. X. *et al.* Electrolytes induce long-range orientational order and free energy changes in the H-bond network of bulk water. *Science Advances* **2**, e1501891 (2016).

17 Ong, S. W., Zhao, X. L. & Eisenthal, K. B. Polarization of water molecules at a charged interface - 2nd harmonic studies of the silica water interface. *Chemical Physics Letters* **191**, 327-335 (1992).

18 Ye, S., Nihonyanagi, S. & Uosaki, K. Sum frequency generation (SFG) study of the pH-dependent water structure on a fused quartz surface modified by an

octadecyltrichlorosilane (OTS) monolayer. *Physical Chemistry Chemical Physics* **3**, 3463-3469 (2001).
19 Bonthuis, D. J., Gekle, S. & Netz, R. R. Profile of the Static Permittivity Tensor of Water at Interfaces: Consequences for Capacitance, Hydration Interaction and Ion Adsorption. *Langmuir* **28**, 7679-7694 (2012).
20 Myalitsin, A., Urashirna, S. H., Nihonyanagi, S., Yamaguchi, S. & Tahara, T. Water Structure at the Buried Silica/Aqueous Interface Studied by Heterodyne-Detected Vibrational Sum-Frequency Generation. *Journal of Physical Chemistry C* **120**, 9357-9363 (2016).
21 Israelachvili, J. & Wennerstrom, H. Role of hydration and water structure in biological and colloidal interactions. *Nature* **379**, 219-225 (1996).
22 Remsing, R. C., Baer, M. D., Schenter, G. K., Mundy, C. J. & Weeks, J. D. The Role of Broken Symmetry in Solvation of a Spherical Cavity in Classical and Quantum Water Models. *The Journal of Physical Chemistry Letters* **5**, 2767-2774 (2014).
23 Cendagorta, J. R. & Ichiye, T. The Surface Potential of the Water–Vapor Interface from Classical Simulations. *The Journal of Physical Chemistry B* **119**, 9114-9122 (2015).
24 Duignan, T. T., Baer, M. D., Schenter, G. K. & Mundy, C. J. Electrostatic solvation free energies of charged hard spheres using molecular dynamics with density functional theory interactions. *The Journal of Chemical Physics* **147**, 161716 (2017).
25 Randles, J. E. B. Structure at the free surface of water and aqueous electrolyte solutions. *Physics and Chemistry of Liquids* **7**, 107-179 (1977).
26 Li, P., Huang, J., Hu, Y. & Chen, S. Establishment of the Potential of Zero Charge of Metals in Aqueous Solutions: Different Faces of Water Revealed by Ab Initio Molecular Dynamics Simulations. *The Journal of Physical Chemistry C* **125**, 3972-3979 (2021).
27 Biesheuvel, P. M. Electrostatic free energy of interacting ionizable double layers. *Journal of Colloid and Interface Science* **275**, 514-522 (2004).
28 Overbeek, J. T. G. The role of energy and entropy in the electrical double layer. *Colloids and Surfaces* **51**, 61-75 (1990).
29 Krishnan, M. Electrostatic free energy for a confined nanoscale object in a fluid. *Journal of Chemical Physics* **138**, 114906 (2013).
30 Krishnan, M. A simple model for electrical charge in globular macromolecules and linear polyelectrolytes in solution. *The Journal of chemical physics* **146**, 205101-205101 (2017).
31 Verwey, E. J. W. & Overbeek, J. T. G. *Theory of the stability of lyophobic colloids*. (Elsevier, 1948).
32 Parsegian, V. A., Rand, R. P. & Fuller, N. L. Direct osmotic-stress measurements of hydration and electrostatic double-layer forces between bilayers of double-chained ammonium acetate surfactants. *Journal of Physical Chemistry* **95**, 4777-4782 (1991).
33 Schlaich, A., dos Santos, A. P. & Netz, R. R. Simulations of Nanoseparated Charged Surfaces Reveal Charge-Induced Water Reorientation and Nonadditivity of Hydration and Mean-Field Electrostatic Repulsion. *Langmuir* **35**, 551-560 (2019).
34 Darlington, A. M. & Gibbs-Davis, J. M. Bimodal or Trimodal? The Influence of Starting pH on Site Identity and Distribution at the Low Salt Aqueous/Silica Interface. *Journal of Physical Chemistry C* **119**, 16560-16567 (2015).
35 Macias-Romero, C., Nahalka, I., Okur, H. I. & Roke, S. Optical imaging of surface chemistry and dynamics in confinement. *Science* **357**, 784-787 (2017).
36 Grunwald, E. & Berkowitz, B. J. The Measurement and Correlation of Acid Dissociation Constants for Carboxylic Acids in the System Ethanol-Water. Activity

Coefficients and Empirical Activity Functions. *Journal of the American Chemical Society* **73**, 4939-4944 (1951).
37 Belskii, V. E., Kudryavtseva, L. A., Derstuganova, K. A., Teitelbaum, A. B. & Ivanov, B. E. Basicity of Aliphatic-Amines in Ethanol. *Bulletin of the Academy of Sciences of the USSR Division of Chemical Science* **30**, 736-738 (1981).
38 Brunner, M., Dobnikar, J., von Grunberg, H. H. & Bechinger, C. Direct measurement of three-body interactions amongst charged colloids. *Physical Review Letters* **92**, 078301 (2004).
39 del Castillo, G. F. D., Hailes, R. L. N. & Dahlin, A. Large Changes in Protonation of Weak Polyelectrolyte Brushes with Salt Concentration-Implications for Protein Immobilization. *Journal of Physical Chemistry Letters* **11**, 5212-5218 (2020).
40 Isom, D. G., Castaneda, C. A., Cannon, B. R. & Garcia-Moreno, B. E. Large shifts in pK(a) values of lysine residues buried inside a protein. *Proceedings of the National Academy of Sciences of the United States of America* **108**, 5260-5265 (2011).
41 Dong, H. T., Du, H. B., Wickramasinghe, S. R. & Qian, X. H. The Effects of Chemical Substitution and Polymerization on the pK(a) Values of Sulfonic Acids. *Journal of Physical Chemistry B* **113**, 14094-14101 (2009).
42 Franzmann, T. M. *et al.* Phase separation of a yeast prion protein promotes cellular fitness. *Science* **359**, 47-54 (2018).
43 Lund, M. & Jonsson, B. A mesoscopic model for protein-protein interactions in solution. *Biophysical Journal* **85**, 2940-2947 (2003).
44 Nott, T. J. *et al.* Phase Transition of a Disordered Nuage Protein Generates Environmentally Responsive Membraneless Organelles. *Molecular Cell* **57**, 936-947 (2015).
45 Radak, B. K. *et al.* Constant-pH Molecular Dynamics Simulations for Large Biomolecular Systems. *Journal of Chemical Theory and Computation* **13**, 5933-5944 (2017).
46 Kim, S. *et al.* Complexation and coacervation of like-charged polyelectrolytes inspired by mussels. *Proceedings of the National Academy of Sciences of the United States of America* **113**, E847-E853 (2016).
47 Baumgartl, J. R., Arauz-Lara, J. L. & Bechinger, C. Like-charge attraction in confinement: myth or truth? *Soft Matter* **2**, 631 (2006).
48 Polin, M., Grier, D. G. & Han, Y. Colloidal electrostatic interactions near a conducting surface. *Physical Review E* **76** (2007).
49 Yücel, H. & Okumuşoğlu, N. T. A new tracking algorithm for multiple colloidal particles close to contact. *Journal of Physics: Condensed Matter* **29**, 465101 (2017).
